# Polymorphism of MHC class IIB in an acheilognathid species, *Rhodeus sinensis* shaped by historical selection and recombination

**DOI:** 10.1186/s12863-019-0775-3

**Published:** 2019-09-13

**Authors:** Hyung-Bae Jeon, Hari Won, Ho Young Suk

**Affiliations:** 1 Department of Life Sciences, Yeungnam University, 280 Daehak-ro, Gyeongsan, Gyeongsangbuk-do 38541 South Korea; 2 Department of Biology, Concordia University, 7141 Sherbrooke W, Montreal, Quebec H4B 1R6 Canada

**Keywords:** *Rhodeus sinensis*, Major histocompatibility complex, Trans-species polymorphism, Balancing selection, Bitterlings, Intronless

## Abstract

**Background:**

*Rhodeus sinensis* is a bitterling species occurring throughout the numerous freshwater systems on the East Asia. Here, we analyzed the diversity of the MHC class IIB (*DAB*) genes from this species, which may offer meaningful insights into evolutionary processes in this species as well as other bitterlings.

**Results:**

Using cDNA and gDNA samples from 50 individuals, we discovered classical 140 allelic sequences that could be allocated into either *DAB1* (*Rhsi-DAB1*) or *DAB3* (*Rhsi-DAB3*). *DAB* sequences completely lacking the intron, but identical or similar to *Rhsi-DAB1*, were also discovered from our gDNA samples, and this intron loss likely originated from the retrotransposition events of processed mDNA. The β1 domain was the most polymorphic in both *Rhsi-DAB1* and *-DAB3*. Putative peptide biding residues (PBRs) in *Rhsi-DAB1*, but not in *Rhsi-DAB3*, exhibited a significant *d*_N_/*d*_S_, presumably indicating that different selection pressures have acted on those two *DAB*s. Recombination between different alleles seemed to have contributed to the increase of diversity in *Rhsi-DAB*s. Upon phylogenetic analysis, *Rhsi-DAB1* and *-DAB3* formed independent clusters. Several alleles from other species of Cypriniformes were embedded in the clade of *Rhsi-DAB1*, whereas *Rhsi-DAB3* clustered with alleles from the wider range of taxa (Cyprinodontiformes), indicating that these two *Rhsi-DAB*s have taken different historical paths.

**Conclusions:**

A great deal of MHC class IIB allelic diversity was found in *R. sinensis*, and gene duplication, selection and recombination may have contributed to this diversity. Based on our data, it is presumed that such historical processes have commonly or differently acted on the polymorphism of *Rhsi-DAB1* and *-DAB3*.

**Electronic supplementary material:**

The online version of this article (10.1186/s12863-019-0775-3) contains supplementary material, which is available to authorized users.

## Background

The major histocompatibility complex (MHC) is a set of genes encoding cell membrane glycoproteins responsible for the initiation of adaptive immune response by displaying antigenic peptide to T lymphocytes in vertebrates [1]. MHC genes are classified into class I and II; class II genes are only expressed on professional antigen-presenting cells (e.g., dendritic cells, macrophages, B lymphocytes), whereas class I genes are expressed on all nucleated cells [1, 2]. A peptide fragment loaded onto an MHC class II molecule is derived from antigens endocytosed, digested within lysosomes and presented to the specific receptors on the surface of CD4^+^ helper T cells [1, 2]. MHC class II molecules are heterodimers consisting of two noncovalently associated homogeneous α (α1 and α2 domains) and β chains (β1 and β2 domains; [1, 2]). A peptide fragment loaded onto an MHC class I molecule is derived from cytosolic proteins of infected cells, and is presented to specific receptors on the surface of CD8^+^ cytotoxic T cells [1, 2]. MHC class I molecules are also heterodimers composed of three α domains and β_2_-microglobulin [1, 2].

Classical MHC class II genes are known to be among the most variable in vertebrate genomes [3–5]. The greatest polymorphism can be found in the β1 domain, where peptide-binding residues (PBRs) are located [3, 4, 6]. In contrast, the level of polymorphism in the α1 domain is relatively low, with a few exceptions [7, 8], even though this domain also plays a role in binding with the antigenic peptide. Three major evolutionary forces are known to contribute to the enormous levels of polymorphism observed in the β1 domain [9]: (i) negative frequency-dependent selection [10–12], (ii) heterozygote advantage [13, 14] and (iii) preferences for MHC dissimilar mates [15–17]. Another well-known evolutionary signature of MHC genes is trans-species polymorphism (TSP), which refers to genetic variants whose origin predates speciation, resulting in the occurrence of shared or similar alleles between different, but related, taxa [18–22]. The existence of TSP also means that there must be common alleles to secure survival or high adaptability even in different species under a specific environment [23]. To learn if TSP exist in a family or order and to infer the related evolutionary factors, however, information about a very certain phylogenetic structure among species in the taxa should be available.

Since the first analysis was attempted in carp [24], MHC genes have been characterized in a wide variety of teleost species [3, 25–28]. Teleost MHC class II can be divided into three major groups, namely, *DA*, *DB* and *DE*, based on their sequence features and phylogenetic clustering patterns [28]. Classical MHC class II genes are only found in *DA* (*DAA*: MHC IIα chain; *DAB*: MHC IIβ chain), whereas *DB* and *DE* generally comprise non-classical MHC genes [28, 29]. Therefore, MHC class II genes found in *DA* show tremendous polymorphisms among individuals, and conservative residues that are thought to form hydrogen bonds with antigen peptides [28]. These features do not perfectly appear in MHC class II genes belonging to DB and DE [28].

The primary aim of this study was to identify the signature of evolutionary forces that have acted on the MHC class IIB (*DAB*) sequences of Korean *Rhodeus sinensis*, one of the most widely distributed bitterling species (Acheilognathidae). Analyzing MHC sequences can offer meaningful insights into evolutionary processes in this or other bitterling species. First, because bitterlings spawn on freshwater mussels, which serve as intermediate host of many infectious organisms in freshwater ecosystems, response to pathogens and immunity may have played an important role in evolutionary processes in this species [30–32]. Second, bitterlings are a representative fish group in which the diversity and evolutionary patterns of *DAB* genes have not been properly characterized. In fact, only a partial investigation has been conducted in *R. ocellatus* [33] and *Pseudorhodeus tanago* [34]. Third, the population sizes of several bitterling species have been declining or at the blink of extinction, due to the introduction of exotic species, climate change and devastation of many natural habitats [35–37], which serves as a good opportunity to investigate how size changes in the population and the resulting genetic drift affects MHC allelic diversity. Finally, *R. sinensis* is found in a variety of rivers with very different environmental features [38–40], providing an excellent opportunity to study the differences in selection pressures acting on MHC genes.

This study consisted of three stages. First, the nearly-complete sequences of *DAB* genes were identified from 50 individuals of *R. sinensis* collected from five different drainages, and the structural and functional characteristics were examined. Second, individual and inter-locus variabilities were examined to discover a signature of evolutionary processes acting on *DAB* diversities in this species. Finally, phylogenetic analyses were conducted to infer the evolutionary history of *DAB* genes in this species upon comparison with other vertebrates.

## Results

### Structure and diversity of MHC class IIB

Among primers used (Table 1), only a single pair, SP-F1 and TM-R1, successfully amplified all 50 individual cDNA samples. This primer pair was designed to anneal the sequences of signal peptide (exon 1) and transmembrane region (exon 6). The intronic sequences linking the six exons were identified from gDNA isolated from 20 Nakdong River samples. The amplification with the SP-F1 and TM-R1 yielded 293 sequences, and a total of 140 novel *DAB* alleles of *Rhodeus sinensis* were detected. Based on the blasting search and comparison with *DAB* sequences of other cypriniform species, the alleles were allocated into either *DAB1* (*Rhsi-DAB1*; *N* = 104) or *DAB3* (*Rhsi-DAB3*; *N* = 36; Additional file 8: Figure S1 and Additional file 9: Figure S2). All allelic sequences identified in this study were deposited to NCBI GenBank with the accession numbers of MG989278 to MG989423.

**Table 1:** PCR primers used for the amplification of DAB sequences in *Rhodeus sinensis*. Data comprise primer name, direction (D), sequence, priming position (Position) and reference (Ref)

Name	D	Sequence (5′ - 3′)	Position	Ref
FishIIBEx2-1F	F	CTGATGCTGTCTGCTTTCACTGGA	SP	1
FIBE-F1	F	CTGATGCTGTCTGCTTTCAC	SP	P
ROSI-R1	R	CGGGATCCCAGTCTACGATG	TM	P
ROSI-R2	R	GATCCCAGTCTACGATGATG	TM	P
ROSI-R3	R	GAGTGTATTCTAGGTGAGAGTGG	exon3 3′ end	P
ROSI-R4	R	CAGGTGCGAGTGAATCTGGT	exon3 middle	P
ROSI-R5	R	CTGAGCTTGACCTTTGGTGC	exon3 5′ end	P
Rooc-Fw	F	CCCATAGTTGACATGATGTCATCTG	intron1	2
Rooc-Rev	R	CATGTGTGACAGGAGGATCAG	intron2	2
DAB1-Rev374	R	CAAGAGTTTCCCGTGTGACAG	intron2	2
DAB1-Fw18	F	AGTTGACATGATGCCATCTGA	intron1	2
DAB-Ex1-Fw	F	CATCCATACTGATGCTGTCTGC	SP	2
Rhod-GSP2	R	CCAGTCTCCGTTAGGCATCTCC	exon3	2
SP-F1	F	CTGATGCTGTCKRCWTTYACYGGA	SP	P
TM-R1	R	TGGTACCAGGATCCTCCC	TM	P
TM-R2	R	TGGKACCAGGATYCTCCC	TM	P
MHC2-TMr	R	TCAGTTTGGTACCAGGATCCTCC	TM	P
Exon-3R	R	TGAGCTTGACCTCTGGTGCCAC	exon3 5′ end	P
THMHCI-3R	R	GCCAGCGTGATCCACCATAC	TM	P

(Ref) 1: Ottová et al., 2007 [41]; 2: Agbali et al., 2010 [33]; P: present study

The sequence of exon 2 was confirmed to be β1 domain region with PBRs through cDNA and gDNA sequencing (Additional file 8: Figure S1 and Additional file 9: Figure S2). Several conserved residues were found and may be related to the function of β1 domain region based on the comparison with mammalian classical *DRB* structure [42]. For example, H81 and N82 are predicted to form hydrogen bonds with antigen peptides (Additional file 8: Figure S1 and Additional file 9: Figure S2). N38, S39 and T40 seem to be responsible for *N*-linked glycosylation, and two conserved cysteine residues, 11C and 76C were thought to form disulfide bridges (Additional file 8: Figure S1 and Additional file 9: Figure S2). In addition, N30 and N59 are residues that are found without exception in all jawed vertebrates, and G46 and Y47 are known to be ray-finned fish specific ([28]; Additional file 8: Figure S1 and Additional file 9: Figure S2). Rhsi-DAB1*04:02 and Rhsi-DAB3*06:01–03 showed a single codon insertion and deletion, respectively, in exon 2 (Additional file 8: Figure S1 and Additional file 9: Figure S2). From exon 3 to 6, overall amino acid sequences were highly conserved with no length variation (Additional file 10: Figure S3, Additional file 11: Figure S4, Additional file 12: Figure S5 and Additional file 13: Figure S6). Exon 3 contained a conserved amino acid motif (from 49 to 65) that seemed to be responsible for binding to CD4 molecule (Additional file 10: Figure S3 and Additional file 11: Figure S4). Two conserved cystein residues (C23 and C29) were predicted to form disulfide bridges in this domain (Additional file 10: Figure S3 and Additional file 11: Figure S4).

In our gDNA analysis, MHC class IIB sequences lacking introns (single exon gene, SEG) were discovered from all 20 individuals collected in the Nakdong River. These sequences were similar or completely identical (Rhsi-DAB1*01:09, −DAB1*03:03, −DAB1*03:04, −DAB1*03:09, −DAB1*03:10, −DAB1*03:16, −DAB1*05:03, −DAB1*05:15 and -DAB1*07:01) to the *Rhsi-DAB1* alleles obtained from the cDNA samples. No SEGs similar or identical to *Rhsi-DAB3* were found.

### Signature of recombination

**Fig. 1:**
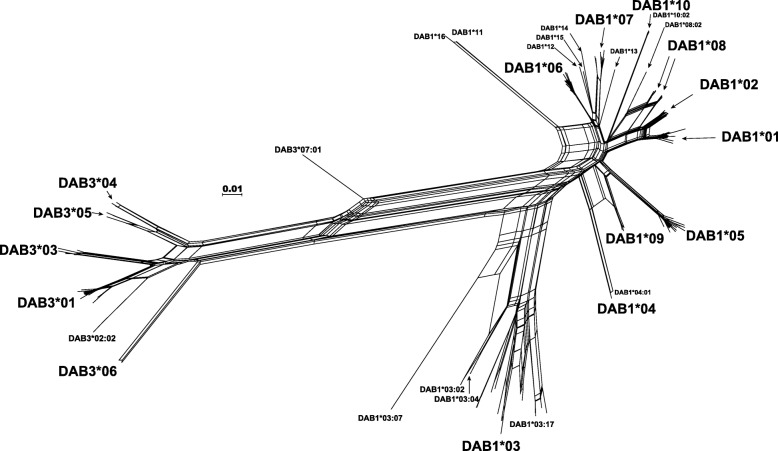
Phylogenetic relationship among the β1 domain sequences of *Rhsi-DAB1* and -*DAB3* reconstructed using Neighbour-Net analysis based on Jukes-Cantor distance model

The result of RDP analysis showed that five *Rhsi-DAB* alleles likely occurred by recombination events in β1 domain region (Table 2). All seven algorithms used in this analysis supported that Rhsi-DAB1*04:01 and -DAB3*07:01 were formed from the recombination between two different alleles (Table 2). Rhsi-DAB1*03:07, −DAB1*03:17 and -DAB1*10:02 could be considered as the recombinant, but not supported by all algorithms. The signature of recombinantion in Rhsi-DAB1*04:01 and -DAB3*07:01 could also be viewed in the network tree analysis, because they did not form clusters with the same allelic groups but instead were located in the middle of the recombination origins (Fig. 1).

**Table 2:** Five putative recombinants detected using seven different testing algorithms (R: RDP, C: CHIMAERA, G: GENECONV, S: SISCAN, B: BOOTSCAN, T: 3SEQ, M: MAXCHI) implemented in RDP4. Significance was indicated by ‘+’. ‘Unknown’ means that a definite parent allele was not estimated, but alleles with the highest probability were indicated in parentheses

Recombinant	Breakpoints		Parent alleles		Algorithm						
	Begin	End			R	G	B	M	C	S	T
DAB3*07:01	1	144	DAB1*09:01	DAB3*01:07	+	+	+	+	+	+	+
DAB1*04:01	232	276	DAB1*01:13	Unknown (DAB1*03:06)	+	+	+	+	+	+	+
DAB1*03:07	225	274	DAB1*03:06	Unknown (DAB1*05:04)	+	+	–	+	+	–	+
DAB1*03:17	165	213	DAB1*03:02	DAB1*03:13	+	–	–	–	–	–	–
DAB1*10:02	82	172	DAB1*09:01	DAB1*03:05	+	–	–	+	+	–	+

### Signature of positive selection

**Fig. 2:**
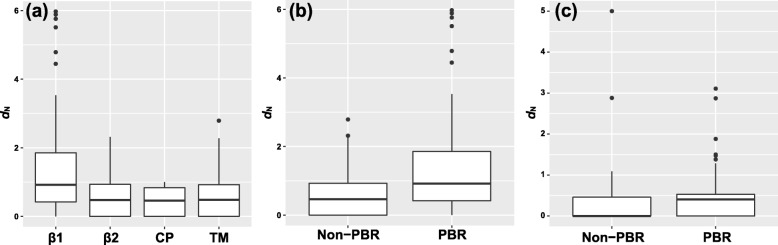
The comparison of nonsynonymous substitution rate (*d*_N_) among major exonal domains (**a**), between non-PBR and PBR in *Rhsi-DAB1* (**b**) and between non-PBR and PBR in *Rhsi-DAB3* (**c**)

The non-synonymous substitution level (*d*_N_/*d*_S_) of β1 domain was significantly high in both *Rhsi-DAB1* (*Z* = 2.967, *P* = 0.037) and *-DAB3* (*Z* = 1.290, *P* = 0.045), whereas no significance was found in other exonal regions (*Rhsi-DAB1*: *Z* = − 2.443, *P* = 1.000; *Rhsi-DAB3*: *Z* = − 1.911, *P* = 1.000; Fig. 2 (a)). Two positive selection models, *M2a* and *M8*, were likely to fit the data significantly better than nearly neutral (*M1a*) and β distribution (*M7*), respectively, in both *Rhsi-DAB1* (Additional file 2: Table S2 and Additional file 3: Table S3) and *Rhsi-DAB3* (Additional file 4: Table S4 and Additional file 5: Table S5). The average *d*_N_/*d*_S_ level of PBRs was specifically higher than that of other codons (non-PBRs) in β1 domain region of *Rhsi-DAB1* (Fig. 2 (b)), though no such a pattern was observed in *Rhsi-DAB3* (Table 3; Fig. 2(c)). The BEB analysis in CODEML revealed that twenty-three codons in *Rhsi-DAB1* β1 (Additional file 2: Table S2 and Additional file 3: Table S3) and fifteen codons in *Rhsi-DAB3* β1 (Additional file 4: Table S4 and Additional file 5: Table S5) showed the signature of positive selection (Table 4; Additional file 6: Table S6). Thirteen putative PBRs were included among those codons in *Rhsi-DAB1* (Additional file 14: Figure S7 and Additional file 15: Figure S8). In *Rhsi-DAB3*, however, only five putative PBRs were included in these fifteen codons (Additional file 16: Figure S9 and Additional file 17: Figure S10). Four different codon-based maximum likelihood tests yielded slightly different results. For example, SLAC and FEL indicated three and ten codons showing the signature of positive selection, respectively, in *Rhsi-DAB1* β1 (Table 4), while no codon was indicated in *Rhsi-DAB3* β1 (Additional file 7: Table S7). MEME detected the signature of positive selection from 23 and three codons in *Rhsi-DAB1* β1 and in *Rhsi-DAB3* β1, respectively (Table 4). FUBAR detected the signature of positive selection from 17 and 10 codons in *Rhsi-DAB1* β1 and in *Rhsi-DAB3* β1, respectively (Table 4).

**Table 3:** The signature of positive selection in β1 domain regions of two Rhsi-DABs. Data comprise the number of residues (Codon), ratio of nonsynonymous vs synonymous substitution (*d*_N_/*d*_S_) and the significance of *Z* test (*P*)

Region	DAB1 β1			DAB3 β1		
	Codon	*d*_N_/*d*_S_	*P*	Codon	*d*_N_/*d*_S_	*P*
PBR	19	2.929	**0.001**	19	1.940	0.123
Non-PBR	72	1.091	0.395	72	0.798	1.000
Total	91	1.533	**0.037**	91	1.034	**0.045**

Statistical significance (< 0.05) was highlighted with bold

**Table 4:** Identification of the codons showing the signature of positive select based on six different models implemented RDP and CODEML. The codons predicted to be PBR were highlighted by red

	5	7	8	9	12	17	22	25	27	28	29	30	33	34	35	36	37	38	41	44	50	52	53	
Rhsi-DAB1																								
SLAC		+																						
FEL	+	+											+		+								+	
MEME	+	+			+		+		+		+				+	+	+	+					+	
FUBAR	+	+		+				+		+			+		+								+	
M2a	+	+		+				+	+	+				+	+								+	
M8	+	+		+				+	+	+				+	+							+	+	
Rhsi-DAB3																								
SLAC																								
FEL																								
MEME				+		+		+																
FUBAR				+				+											+					
M2a								+											+					
M8								+											+				+	
	54	57	58	60	61	63	64	65	67	68	71	72	74	75	77	78	82	83	84	85	86	88	90	91
Rhsi-DAB1																								
SLAC													+					+						
FEL							+		+				+	+				+						
MEME		+					+		+	+			+	+	+			+	+	+		+	+	
FUBAR		+		+			+		+		+		+	+	+			+						
M2a			+	+			+		+		+			+	+		+	+						
M8			+	+			+		+		+	+	+		+		+	+		+		+		
Rhsi-DAB3																								
SLAC																								
FEL																								
MEME																								
FUBAR	+		+			+				+	+		+					+						
M2a			+	+	+	+	+	+		+	+		+					+		+		+		
M8			+	+	+	+	+	+		+	+		+					+		+		+		

### Phylogenetic analysis

**Fig. 3:**
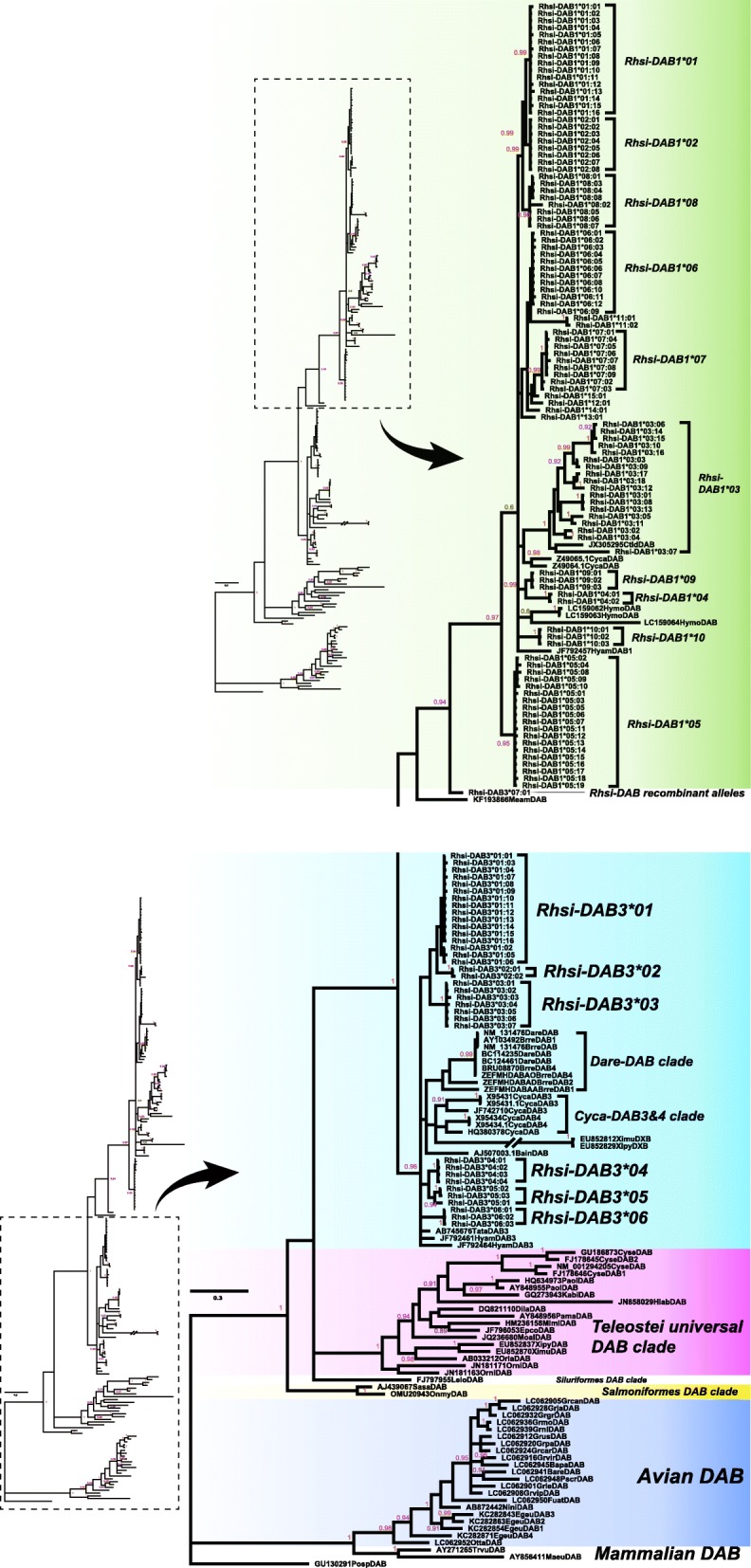
Phylogenetic placement of *Rhsi-DAB* β1 domain sequences among the MHC class IIB alleles of major telosts and vertebrates. A total of 44 vertebrate species were used as outgroup. Bayesian inference tree was reconstructed under GTR + I + G model

Upon NJ tree analysis based on the β1 domain of *DABs*, a total of 16 and seven allelic groups were identified in *Rhsi-DAB1* and in *Rhsi-DAB3*, respectively. The allelic IDs were determined based on this NJ tree clustering (Additional file 18: Figure S11). In BI tree, however, some of the allelic groups identified in NJ tree failed to form a clear monophyletic cluster (Fig. 3). Like NJ tree, *Rhsi-DAB1* and *-DAB3* alleles formed completely independent clusters in the BI tree (Fig. 3). Within the *Rhsi-DAB1* allelic groups, Rhsi-DAB1*05 was found to form a sister to other groups (Fig. 3). Several alleles from other Cypriniformes species, such as *Hymo-DAB, Hyam DAB1, Ctid-DAB* and *Cyca-DAB*, were embedded in the clade of *Rhsi-DAB1* allelic groups (Fig. 3). *Rhsi-DAB3* allelic groups clustered with alleles from the wider range of taxa (Cyprinodontiformes) including *Dare-DAB1, Dare-DAB2, Dare-DAB4, Cyca-DAB3, Cyca-DAB4, Ximu-DXB, Xipy-DXB, Tata-DAB3* and *Hyam DAB3* (Fig. 3). *Rhsi-DAB* alleles did not cluster with the alleles from other teleost orders, such as Salmoniformes, Siluriformes, Perciformes, Pleuronectiformes and Syngnathiformes as well as non-teleost vertebrates (Fig. 3).

## Discussion

Our analysis of MHC class IIB variation in *Rhodeus sinensis* revealed very large numbers of *DAB* alleles, which was only from 50 individuals. Characterization of *DAB* was attempted in two other bitterling species, *R. ocellatus* [33] and *Pseudorhodeus tanago* [34], as mentioned in Introduction, and only 17 [33] and 16 [34] sequence variants were identified, respectively. However, direct comparison of diversity with these species are not likely to be possible, since *Pseudorhodeus tanago* was listed as critically endangered species [34] and the MHC class IIB of *R. ocellatus* was analyzed simply because of the need to gather information on MHC allele diversity in behavioral experiments [33]. Our study aimed to analyze nearly complete *DAB* sequences, and all 140 alleles characterized included a signal peptide, two extra-cellular domains (β1 and β2), a connecting peptide, a trans-membrane region and a cytoplasmic domain. Upon comparison with other teleost or vertebrate species at the codon level, major residues predicted to be responsible for the processes in adaptive immune response were completely identified, indicating that all of the *Rhsi-DAB* sequences could be regarded as functional and classical alleles.

One of the surprising observations from this study was that SEGs (MHC class IIB lacking the introns) were discovered from gDNA samples. Intron loss has been reported across many vertebral genes (e.g., *hsp70* in zebrafish [43], the histone gene family in vertebrates [44], claudin and olfactory receptor genes in teleosts [45]). Intron loss in MHC class IIB was reported in two teleost species, *Gasterosteus aculeatus* and *Tetraodon nigroviridis* [45]. It has been proposed that intron loss could be mediated by random or homologous recombination of ancestral genome with the DNA fragment reverse-transcribed from processed mRNA [46–48]. Considering that partial intron loss was not observed in our study, these SEGs likely originated from the insertion of processed cDNA into a nonhomologous genome location (retrotransposition event; see [45]). Nine variants of SEGs were found to be identical or similar to *Rhsi-DAB1*, while no SEG similar to *Rhsi-DAB3* was found, indicating that this event has probably occurred since the divergence between *DAB1* and *DAB3* were completed. Given that intron loss is more common in highly expressed genes, such as housekeeping genes [46–48], the reason why intron loss was observed only in *Rhsi-DAB1* can be deduced from the difference in expression level. However, it is premature to evaluate the difference in expression level without comparing mRNA levels. It remains to assure that intron loss is prevalent in all *R. sinensis* populations, becaue all of the gDNA samples used were obtained from the Nakdong River. It is thus necessary to investigate the existence of SEG from other drainages in future study.

The phylogenetic separation between *DAB1* and *DAB3* was evident in our BI tree reconstructed using the β1 domain as shown in a study of the Cypriniformes species [49]. Multiple loci may exist within *Rhsi-DAB1*, given that more than three alleles were frequently found in a single individual sample. Assumption based only on the BI tree revealed that Rhsi-DAB1*05 is the most prominent locus candidate, and the remaining allelic groups were likely divided into five; (1) Rhsi-DAB1*01, −*02 and -*08; (2) Rhsi-DAB1*06, −*07, −*11, −*12, −*13, −*14 and -*15; (3) Rhsi-DAB1*03; (4) Rhsi-DAB1*04 and -*09; (5) Rhsi-DAB1*10. No individuals with all six locus-candidates were observed, and the number of *Rhsi-DAB1* loci possessed by each individual appears to be different, given that the individuals showed considerably different composition of locus-candidates. Although its diversity was not as high as *Rhsi-DAB1*, there were roughly three locus-candidates in *Rhsi-DAB3*, if speculating based solely on the BI tree. In contrast to the enormous diversity observed in our results, no evidence of gene duplication was reported in bitterling species to dates [17, 33]. Although gene duplication of *DABs* may be thought of as a species-specific evolutionary event that occurred in *R. sinensis*, it may be wise to suspend judgment until tests using various primers are conducted. It has often been found that slightly different primer sets, which were designed for almost the same region, exhibited amplification bias yielding different sequences or different number of alleles.

In both *Rhsi-DAB1* and -*DAB3*, the β1 domain region was the most polymorphic, and the signature of positive selection was clearly detected. The amino acid sequences of the remaining regions were highly conservative with no clear sign of positive selection. In the β1 domain region, the polymorphism level of *Rhsi-DAB3* was less than that of *Rhsi-DAB1*, which was also observed in other studies of Cypriniformes species [50, 51]. In addition, *Rhsi-DAB1*, but not *Rhsi-DAB3*, showed a large and significant *d*_N_/*d*_S_, when considering only PBRs. Presumably, different types of selection pressures have acted on *Rhsi-DAB1* and -*DAB3* (see also [52, 53]). Our results suggest that while *Rhsi-DAB1* is reponsible for the binding to a wide range of pathogenic peptides, *Rhsi-DAB3* may be associated with detection of some specialized antigenic peptides. If so, highly variable *Rhsi-DAB1* might have been specialized to interact with many different types of pathogens, which has probably led to the specific pathogen-mediated selections (see also [54, 55]). This explanation is highly plausible, considering that a slight difference in amino acid sequence among MHC class IIB alleles may be associated with adaptation to different pathogens (e.g., [56–59]). If the pathogenic fauna and allelic variation at the population level are studied, it will be possible to examine whether the allelic diversity of *Rhsi-DAB1* is the result of adaptation to different types of pathogens.

Recombination between different alleles seemed to have at least partially contributed to the increase or maintenance of diversity in *Rhsi-DABs*. Several other studies also reported examples of MHC class IIB alleles originating from recombination [3, 60–62]. In this study, some of the recombination events occurred between *Rhsi-DAB1* and -*DAB3*. Since *Rhsi-DAB1* and -*DAB3* were expected to differ in terms of their diversity and fuctional range, as mentioned above, further studies should be conducted to determine if the alleles originating from interlocus recombinations have resulted in adaptive benefits in their habitats.

The existence of the phylogenetic lineages containing alleles of *Rhsi-DAB1*, *Hyam-DAB1, Hymo-DAB* and *Ctid-DAB* probably suggests that the occurrence of *Rhsi-DAB1* alleles should predate the ages of diversification into the present species in Cypriniformes. Specifically, the sister relationship between Rhsi-DAB1*03 and *Ctid-DAB* showed that the Rhsi-DAB1*03 allelic group likely have existed before the divergence of Leuciscidae and Acheilognathidae that could be estimated to be around 66 million years ago (MYA) when speculating based on the divergence time between the representative genera of those two families, *Ctenopharyngodon* and *Rhodeus* [63, 64]. *Rhsi-DAB3* formed a cluster with Cyprinodontiformes, which probably means that the sequence structure of *Rhsi-DAB3* has been maintained as more conserved rather than that of *Rhsi-DAB1*. Previous studies estimated that Cyprinodontiformes diverged from other teleosts at around 229.9 MYA [63, 64]. It thus can be predicted that the strength of selection pressure acting on *DAB3* might definitely be weaker than that acting on *DAB1*, considering that the habitat range of Cyprinodontiformes is much wider than Cypriniformes.

Our analysis revealed the evolutionary processes that have at least partially contributed to the formation of *DAB* diversity in *R. sinensis*. What needs to be revealed or confirmed in the future can be summarized as follows. First, what adaptive importance does the alleles identified here have? Second, did the alleles were born before or after the birth of this species? These questions can also be resolved at least in part through the investigation of allelic frequency among *R. sinensis* populations inhabiting different environments or direct comparison among species of the same genus (i.e., *Rhodeus*) and with other major genus species (i.e., *Tanakia* or *Acheilognathus*) in Acheilognathidae. Finally, are the alleles found here products of the same gene or other (duplicated or somewhat distant) genes? Genomic analysis, which has become highly advanced and inexpensive in recent years, will provide a close answer to this question.

## Conclusion

Using cDNA and gDNA samples from 50 individuals of *R. sinensis*, a great deal of MHC class IIB allelic diversity was found, and gene duplication, selection and recombination may have contributed to this diversity. A total of 140 allelic sequences could be allocated into two different loci, *Rhsi-DAB1* and *-DAB3*. Numerous variants of MHC IIB lacking the introns were found from our gDNA samples and these sequences appeared to be historically derived from the retrotransposition events of processed mRNA. Upon robust phylogenetic analysis, *Rhsi-DAB1* and *-DAB3* formed completely independent clusters. Based on our data, it is presumed that such historical processes have commonly or differently acted on the polymorphism of *Rhsi-DAB1* and *-DAB3*.

## Methods

### Sampling

Ten individuals of *Rhodeus sinensis* were collected each from five different rivers (Han, Hyeongsan, Mangyeong, Nakdong and Tamjin) on the Korean Peninsula for the extraction RNA samples. Twenty individuals were additionally collected from the Nakdong River and used for the analysis of genomic sequence structure.

### Extraction of RNA and DNA

RNA was isolated from the brain tissue of each individual using TRIzol (Invitrogen, Carlsbad, CA, USA) reagent according to the manufacturer’s protocol. Before tissue removal for the RNA extraction, each individual fish was euthanized with MS-222 (250 mg/L; Sigma-Aldrich, St. Louis, MO, USA). Brain tissues were used was since they were the samples that should also be analyzed to investigate the expression of non-immune genes. Complementary DNA (cDNA) was synthesized using RNA (800–2000 ng) extracted, oligo-dT and GoScript™ Reverse Transcriptase (Promega, Madison, WI, USA). Genomic DNA (gDNA) was extracted from muscle using a DNeasy Blood and Tissue kit in accordance with the manufacturer’s protocol (Qiagen, Dusseldorf, Germany). All individuals used for DNA and RNA extraction are housed in the Department of Life Science at Yeungnam University as a 95% ethanol sample. Our sampling and experimental procedure were approved by the Yeungnam University Institutional Animal Care and Use Committee (Protocol # 2015013). The concentration of genetic samples was measured using MaestroNano (Maestrogen, Hsinchu City, Taiwan).

### Primers design, PCR, cloning and sequencing

The universal primers designed for cyprinids in previous studies [33, 41] were used to obtain the draft *DAB* sequences of *R. sinensis* (Table 1). Specific primers were also designed based on the draft sequences obtained (Table 1). Each 50 μL mixture for PCR amplification contained 50–100 ng of DNA (cDNA or gDNA), 1 × PCR buffer, 3 mM MgCl_2_, 0.25 mM of each forward and reverse primer, 0.2 mM dNTP and 0.25 unit of *Taq* DNA polymerase (Solgent, Daejeon, South Korea). PCR amplification was performed using GenePro (Bioer, Hangzhou, China) under the following program setup: 94 °C for 10 min, 35 cycles of 40s at 94 °C, 45 s at 52–64 °C (depending on the primers) and 50s at 72 °C, and 72 °C for 10 min. The amplified products were ligated into the pGEM-T Easy vector (Promega) and transformed into *E. coli* DH5α. Ten to sixteen white colonies were selected from each individual for the amplification by SP6 and T7 primer set (*T*_a_ = 56 °C). Being successfully identified on a 2% agarose gel, the PCR products were purified and sent for the commercial sequencing to Macrogen Inc. (Seoul, South Korea).

The nucleotide sequences obtained from cDNA or gDNA samples were regarded as a valid allelic sequence only when identified in at least two separated clones and two different individuals to avoid the possibility of artifacts. To examine the possibility of cross-over contamination, a negative control tube containing purified water instead of a DNA sample was placed in every amplification sample set. All data used in the analyses included only those with no amplification reaction at the negative control. Analyses of cDNA and gDNA samples were performed in a completely separated state in time and space. The sequences identified as valid were aligned using CLUSTALX [65] implemented in GENEIOUS v.9.1.8 [66]. The *DAB* alleles were named following the nomenclature (locus*allelic group:protein sequence) [67].

### Tests of recombination

RDP v.4.5 (Recombination Detection Program; [68, 69]) was used to identify the signature of gene recombination in β1 domain region between different allelic sequences based on seven different algorithms including RDP [68], Chimaera [70], Geneconv [71], SiScan [72], Bootscan [69], 3Seq [73] and Maxchi [74]. For the visualization of the recombination events, phylogenetic network of *DAB* alleles was reconstructed using Neighbour-Net analysis based on Jukes-Cantor distance model implemented in SplitsTree4 [75].

### Tests of positive selection

The putative PBRs in β1 domain region were identified based on the comparison with the sequences characterized in the previous studies [42, 76, 77]. The ratio of non-synonymous (*d*_N_) and synonymous (*d*_S_) substitutions (*ω*) was estimated by Nei-Gojobori method [78] with 2000 bootstrap replicates and modified under Juke Cantor corrections, which was used to determine the strength of historical selection pressure acting on *DAB* sequences.

The signature of positive selection was detected for each codon in the *DAB* allelic sequences using HyPhy package [79] implemented in DataMonkey web server (http://www.datamonkey.org/), where four different codon-based maximum likelihood tests, namely, SLAC (Singel Likelihood Ancestry Counting), FEL (Fixed Effects Likelihood), MEME (Mixed Effects Model of Evolution) and FUBAR (Fast Unconstrained Bayesian AppRoximation) were used. For this purpose, phylogenetic relationship was reconstructed under the default setting in DataMonkey.

Each codon was also tested for the signature of positive selection using CODEML implemented in PAMLX package [80, 81]. For this test, Bayesian posterior probability (BPP) was calculated based on the Bayes empirical Bayes (BEB) method, and the codon was regarded as showing the significant signature of positive selection when the BPP was greater than 95%. Maximum likelihood (ML) phylogenetic tree was reconstructed using rapid bootstrap analyses (1000 replicates) implemented in RAxML-GUI v.1.5 [82], which was used for the input resource in CODEML. The likelihood ratio tests (LRT) were carried out to compare codon-based models, for example, between *M*1a (nearly neutral) and *M*2a (positive selection), between *M*7 (beta distribution) and *M*8 (beta distribution and positive selection), and between *M*0 (one-ratio) and *M*3 (discrete).

### Phylogenetic structure

Neighbor-joining (NJ) tree analysis [83] was performed using the MEGA v.7 [84] under 1000 bootstrapping to check whether *DAB* alleles were divided into evolutionary lineages. To detect TSP across vertebrate, Bayesian inference (BI) phylogenetic analysis was performed using MrBayes v.3.2.3 [85] under the following options: four heated chains, 40,000,000 generations and sampling tree at each 1000 generation. GTR + I + G model was selected as the best fit for the BI analysis using jModelTest v.2.0 [86] under Akaike Information Criterion (AIC; [87]). The first 25% generations on each run was discarded as burn-in. FigTree v.1.4.2 [88] was used to visualize the consensus phylogenetic trees and posterior probabilities on the nodes. A total of 44 vertebrate species were used as outgroup taxa (Additional file 1: Table S1).

## Additional files

Additional file 1: **Table S1.** The information of 44 vertebrate species used as outgroup in the BI tree phylogenetic analyses. (XLSX 12 kb)

Additional file 2: **Table S2.** Summary of models that were used to discover the signature of selection across codons of *Rhsi-DAB1* β1 domain. Data were from six models and comprise the logarithm of the likelihood (ln*L*), codons showing the signature of positive selection and number of parameters estimated in the model. (XLSX 10 kb)

Additional file 3: **Table S3.** The likelihood ratio tests (LRT) performed to verify the signature of positive selection in each nested codon-based model comparison for *Rhsi-DAB1* β1 domain. (XLSX 9 kb)

Additional file 4: **Table S4.** Summary of models that were used to discover the signature of selection across codons of *Rhsi-DAB1* β3 domain. Data were from six models and comprise the logarithm of the likelihood (ln*L*), codons showing the signature of positive selection and number of parameters estimated in the model. (XLSX 10 kb)

Additional file 5: **Table S5.** The likelihood ratio tests (LRT) performed to verify the signature of positive selection in each nested codon-based model comparison for *Rhsi-DAB3* β1 domain. (XLSX 9 kb)

Additional file 6: **Table S6.** The likelihood ratio tests (LRT) performed to verify the signature of positive selection in each nested codon-based model comparison for *Rhsi-DAB1* β1 domain. (XLSX 11 kb)

Additional file 7: **Table S7.** The likelihood ratio tests (LRT) performed to verify the signature of positive selection in each nested codon-based model comparison for *Rhsi-DAB3* β1 domain. (XLSX 11 kb)

Additional file 8: **Figure S1.** Amino acid sequences of *Rhsi-DAB1* β1 domain aligned using CLUSTALX. Dashes were added to represent gaps made in alignment procedure. Various color shading represent the functional regions; black: peptide-backbone interacting residues predicted based on mammalian studies, red: positions 81H and 82 N, yellow: cysteines predicted to form a disulfide bridge, cyan: conserved N-glycosylation motifs, green: highly conserved residues among jawed vertebrates, pink: peptide binding region predicted based on human DRB study, and orange: ray-finned specific residues. (DOCX 1440 kb)

Additional file 9: **Figure S2.** Amino acid sequences of *Rhsi-DAB3* β1 domain aligned using CLUSTALX. Dashes were added to represent gaps made in alignment procedure. Various color shading represent the functional regions; black: peptide-backbone interacting residues predicted based on mammalian studies, red: positions 81H and 82 N, yellow: cysteines predicted to form a disulfide bridge, cyan: conserved N-glycosylation motifs, green: highly conserved residues among jawed vertebrates, pink: peptide binding region predicted based on human DRB study, and orange: ray-finned specific residues. (DOCX 513 kb)

Additional file 10: **Figure S3.** Amino acid sequences of *Rhsi-DAB1* β2 domain aligned using CLUSTALX. Specific functional regions with conserved sequences were highlighted by color shadings; pink: residues binding to CD4, and yellow: cysteines predicted to form a disulfide bridge. (DOCX 1270 kb)

Additional file 11: **Figure S4.** Amino acid sequences of *Rhsi-DAB3* β2 domain aligned using CLUSTALX. Specific functional regions with conserved sequences were highlighted by color shadings; pink: residues binding to CD4, and yellow: cysteines predicted to form a disulfide bridge. (DOCX 446 kb)

Additional file 12: **Figure S5.** Amino acid sequences of *Rhsi-DAB1* CP, TM and CY domain regions aligned using CLUSTALX. (DOCX 846 kb)

Additional file 13: **Figure S6.** Amino acid sequences of *Rhsi-DAB3* CP, TM and CY domain regions aligned using CLUSTALX. (DOCX 467 kb)

Additional file 14: **Figure S7.** The positive selection signatures in the codons of *Rhsi-DAB1* β1 region were represented based on the mean *ω* weighted by the posterior probabilities under the models, *M2a* (a) and *M8* (b). (DOCX 78 kb)

Additional file 15: **Figure S8.** The signature of positive selection was tested with the posterior probability for each codon in *Rhsi-DAB1* β1 region under the models, *M2a* (a) and *M8* (b). (DOCX 130 kb)

Additional file 16: **Figure S9.** The positive selection signatures in the codons of *Rhsi-DAB3* β1 region were represented based on the mean *ω* weighted by the posterior probabilities under the models, *M2a* (a) and *M8* (b). (DOCX 75 kb)

Additional file 17: **Figure S10.** The signature of positive selection was tested with the posterior probability for each codon in *Rhsi-DAB3* β1 region under the models, *M2a* (a) and *M8* (b). (DOCX 131 kb)

Additional file 18: **Figure S11.** Phylogenetic relationship among *Rhsi*-DAB β1 alleles reconstructed based on NJ tree analysis. (DOCX 696 kb)

Additional file 19: Supplementary data. All sequences (in fasta format) reported in this study. (DOCX 25 kb)

## Data Availability

All sequences reported in this study can be retrieved from NCBI GenBank (accession numbers: MG989278 to MG989423) or ‘Additional file 19: Supplementary data’ in this article.

## Abbreviations

BI: Bayesian inference
cDNA: Complementary DNA
gDNA: Genomic DNA
LRT: Likelihood ratio test
MHC: Major histocompatibility complex
ML: Maximum likelihood
MYA: Million years ago
NJ: Neighbour-joining
PBR: Peptide binding residue
SEG: Single exon gene
TSP: Trans-species polymorphism
