# Supplementary figures and images for: Polymorphism of MHC class IIB in an acheilognathid species, Rhodeus sinensis shaped by historical selection and recombination
Source: BMC Genet. 2019 Sep 13;20:74. doi: 10.1186/s12863-019-0775-3 (PMC6743125; doi:10.1186/s12863-019-0775-3)

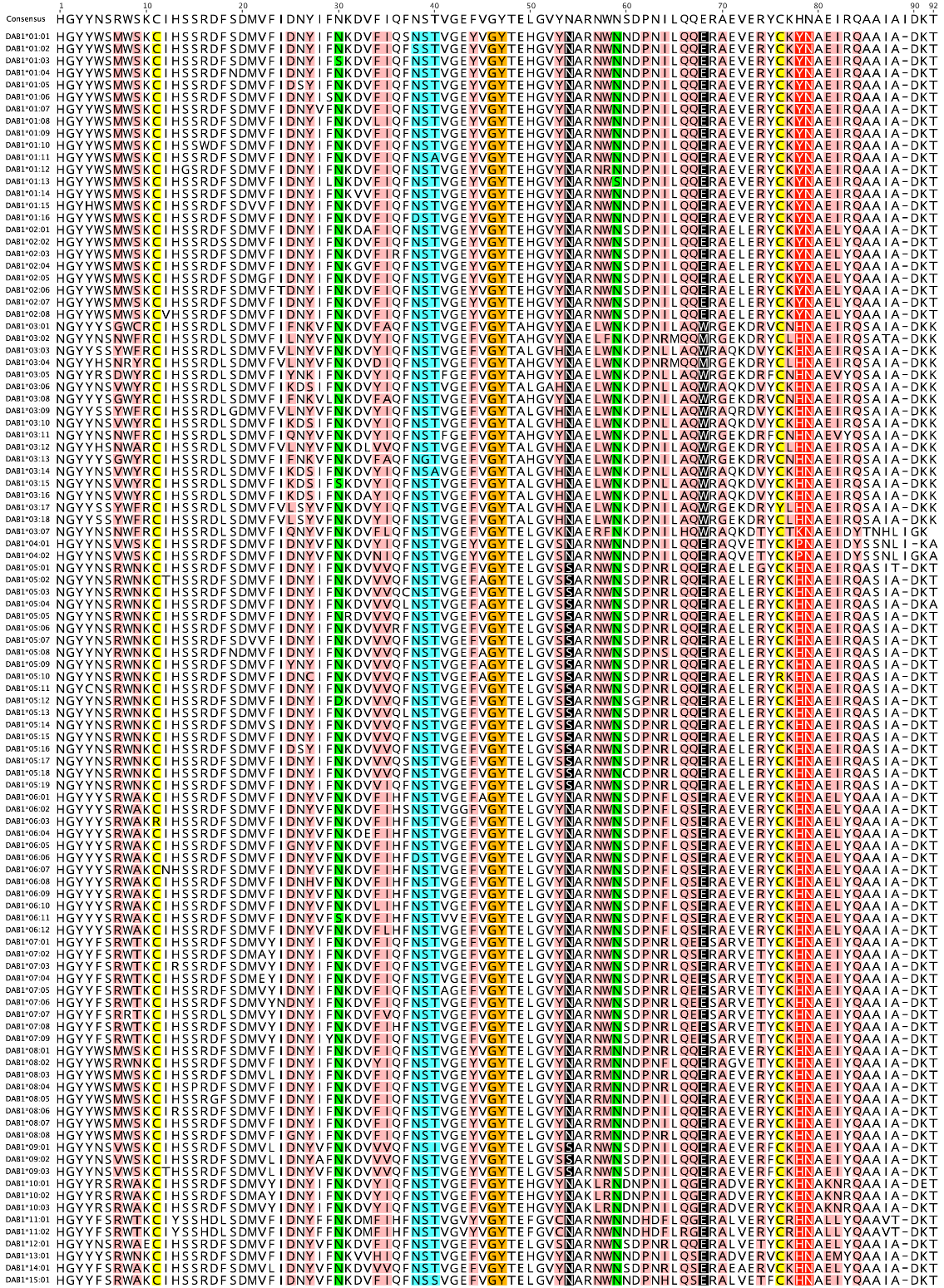

Supplement: Supplementary file 8 — Figure S1. Amino acid sequences of Rhsi-DAB1 β1 domain aligned using CLUSTALX. Dashes were added to represent gaps made in alignment procedure. Various color shading represent the functional regions; black: peptide-backbone interacting residues predicted based on mammalian studies, red: positions 81H and 82 N, yellow: cysteines predicted to form a disulfide bridge, cyan: conserved N-glycosylation motifs, green: highly conserved residues among jawed vertebrates, pink: peptide binding region predicted based on human DRB study, and orange: ray-finned specific residues. (DOCX 1440 kb) [file 12863_2019_775_MOESM8_ESM.docx]

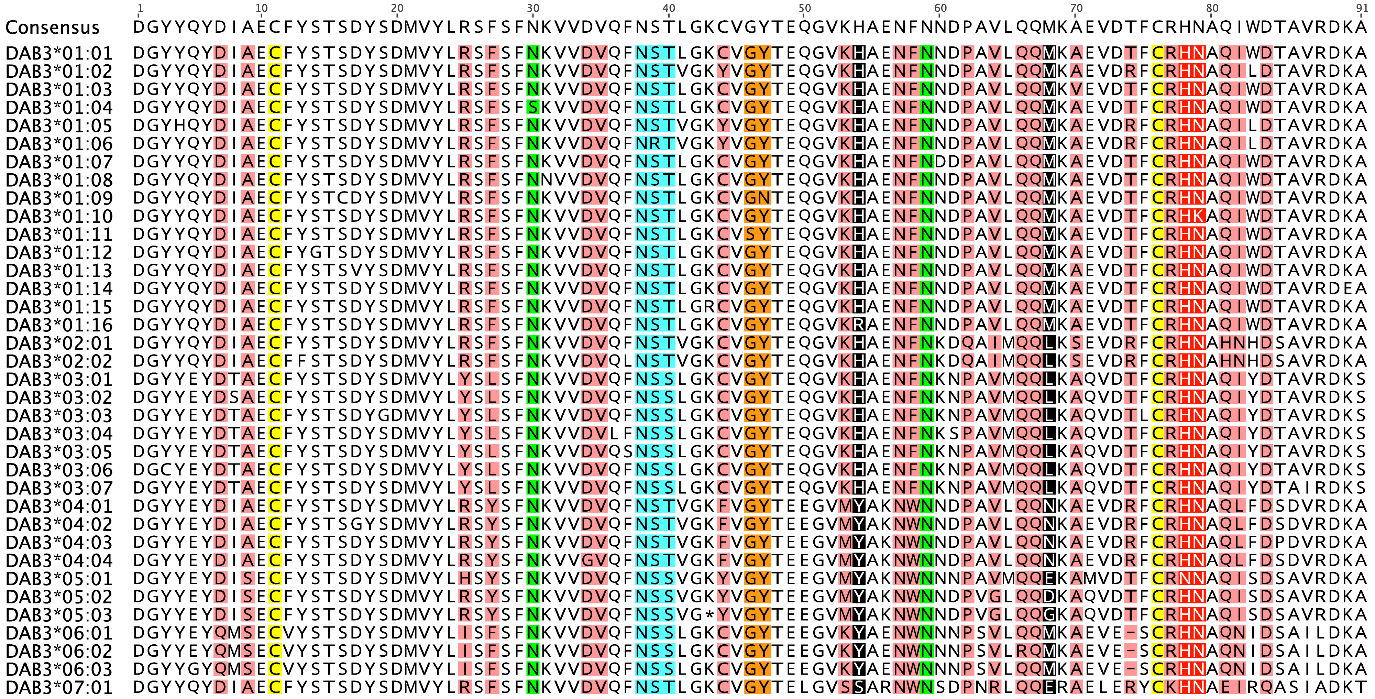

Supplement: Supplementary file 9 — Figure S2. Amino acid sequences of Rhsi-DAB3 β1 domain aligned using CLUSTALX. Dashes were added to represent gaps made in alignment procedure. Various color shading represent the functional regions; black: peptide-backbone interacting residues predicted based on mammalian studies, red: positions 81H and 82 N, yellow: cysteines predicted to form a disulfide bridge, cyan: conserved N-glycosylation motifs, green: highly conserved residues among jawed vertebrates, pink: peptide binding region predicted based on human DRB study, and orange: ray-finned specific residues. (DOCX 513 kb) [file 12863_2019_775_MOESM9_ESM.docx]

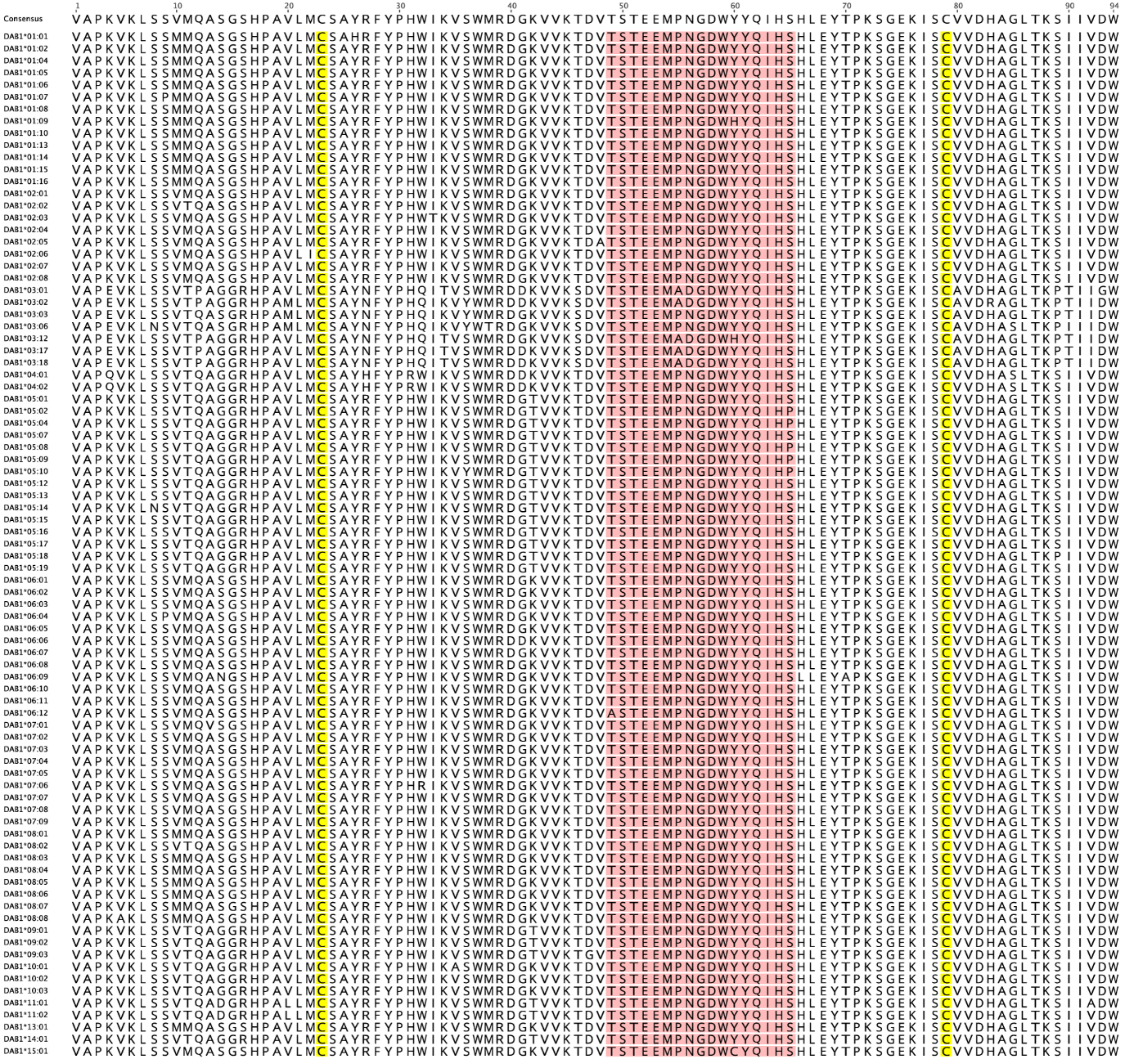

Supplement: Supplementary file 10 — Figure S3. Amino acid sequences of Rhsi-DAB1 β2 domain aligned using CLUSTALX. Specific functional regions with conserved sequences were highlighted by color shadings; pink: residues binding to CD4, and yellow: cysteines predicted to form a disulfide bridge. (DOCX 1270 kb) [file 12863_2019_775_MOESM10_ESM.docx]

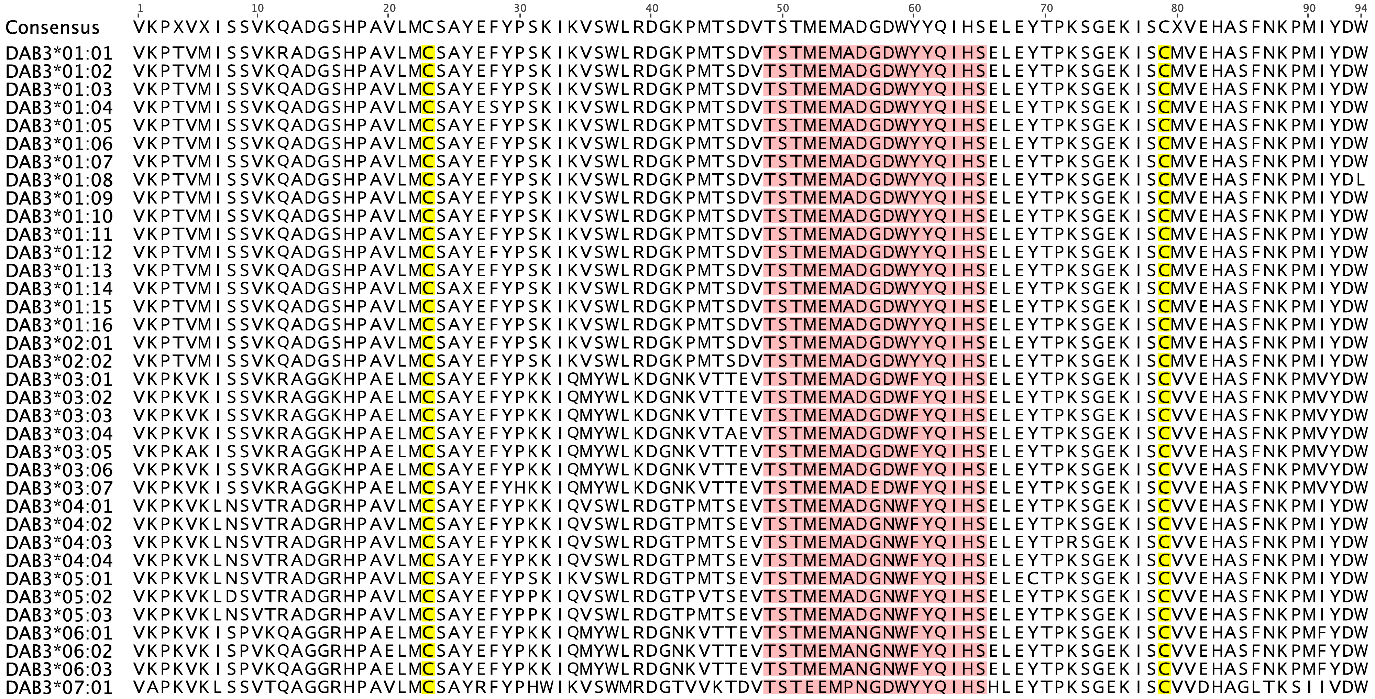

Supplement: Supplementary file 11 — Figure S4. Amino acid sequences of Rhsi-DAB3 β2 domain aligned using CLUSTALX. Specific functional regions with conserved sequences were highlighted by color shadings; pink: residues binding to CD4, and yellow: cysteines predicted to form a disulfide bridge. (DOCX 446 kb) [file 12863_2019_775_MOESM11_ESM.docx]

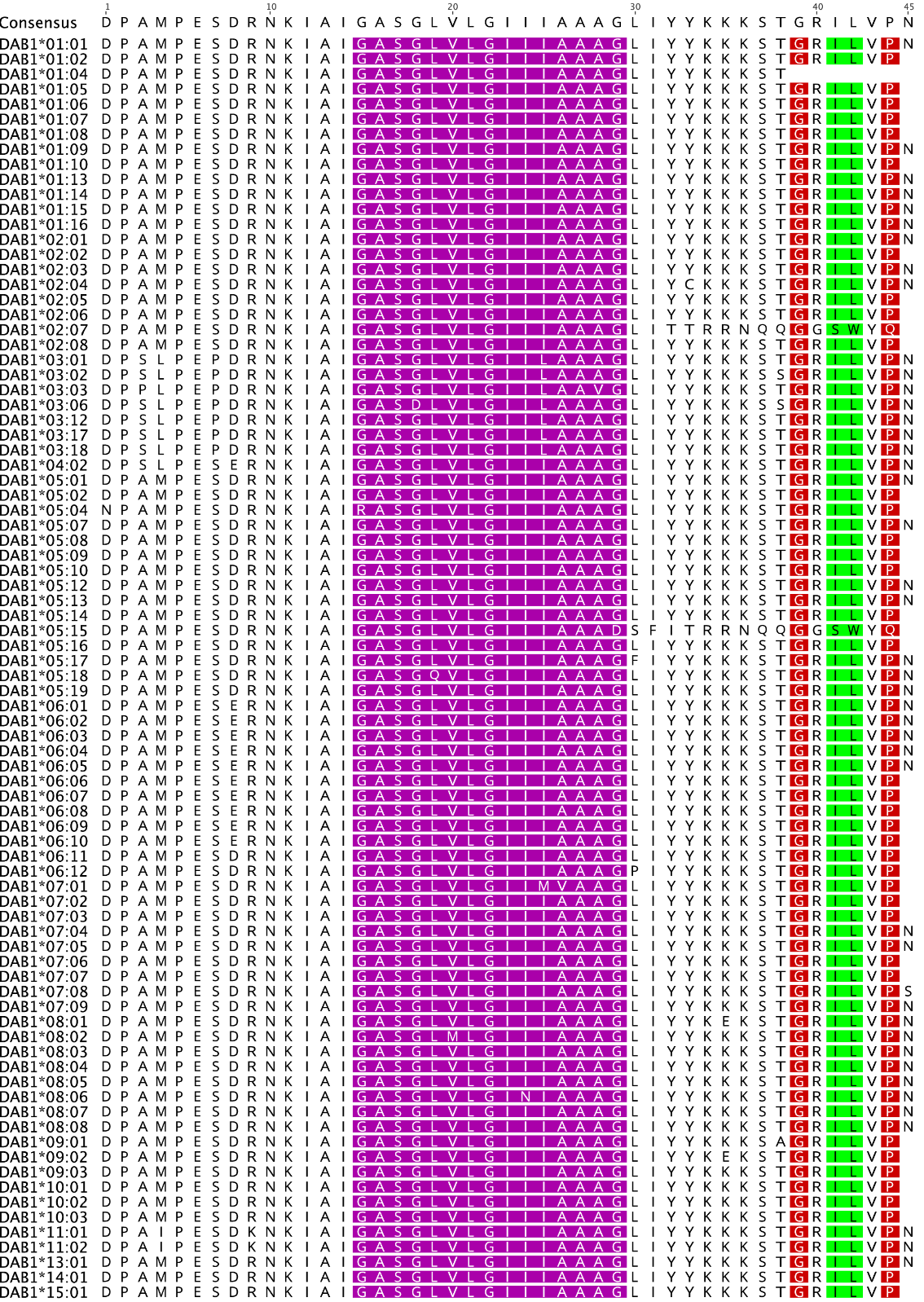

Supplement: Supplementary file 12 — Figure S5. Amino acid sequences of Rhsi-DAB1 CP, TM and CY domain regions aligned using CLUSTALX. (DOCX 846 kb) [file 12863_2019_775_MOESM12_ESM.docx]

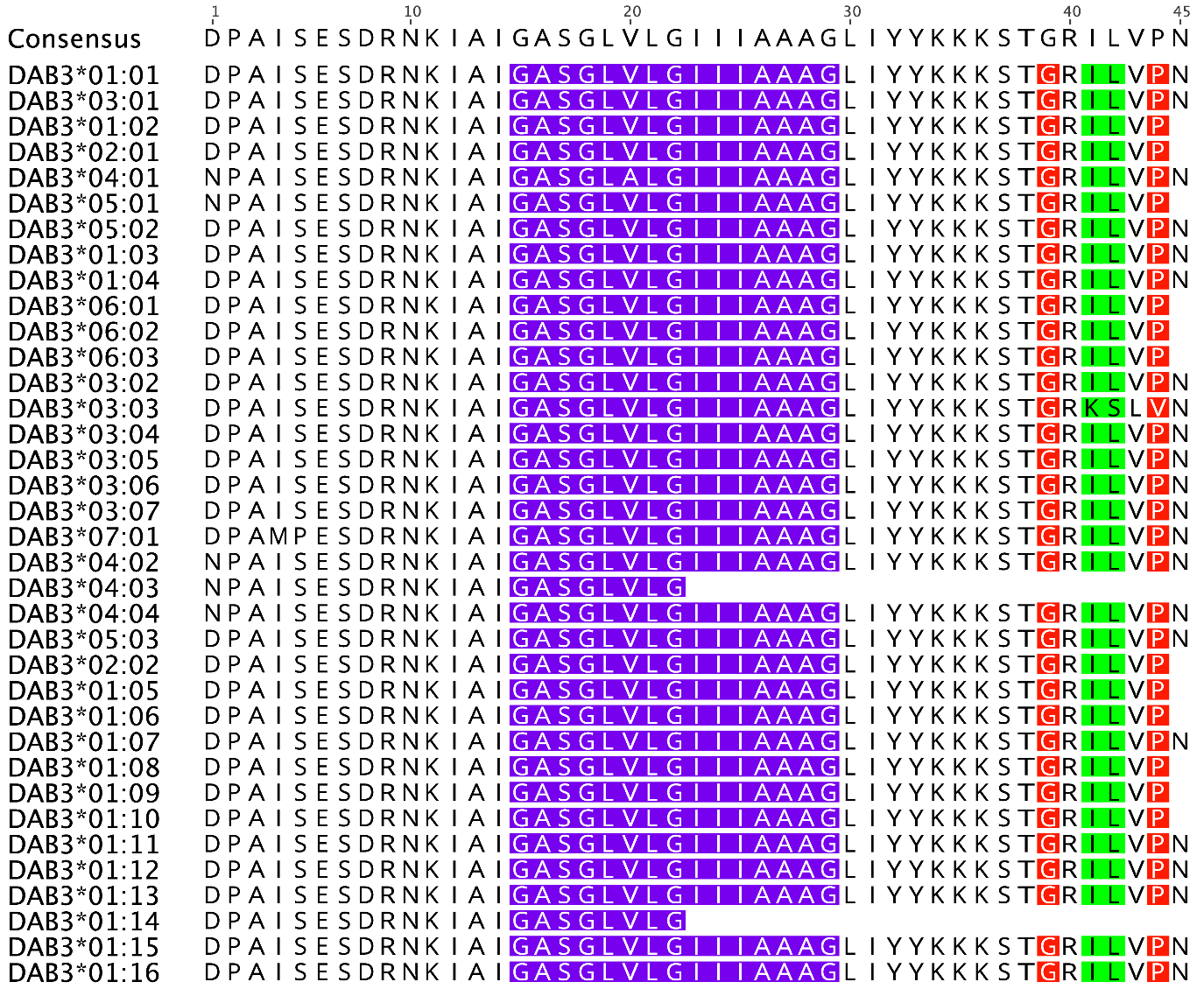

Supplement: Supplementary file 13 — Figure S6. Amino acid sequences of Rhsi-DAB3 CP, TM and CY domain regions aligned using CLUSTALX. (DOCX 467 kb) [file 12863_2019_775_MOESM13_ESM.docx]

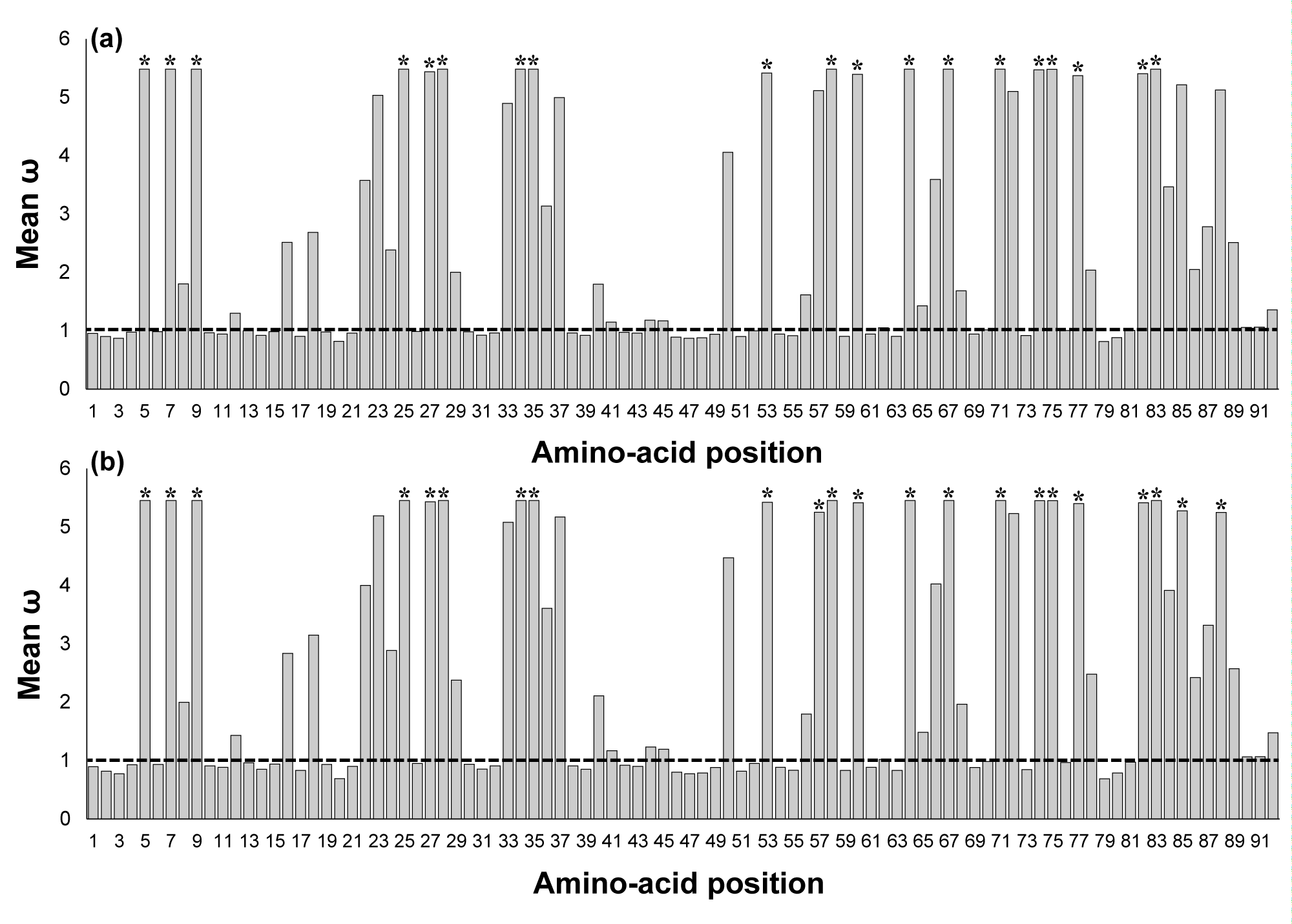

Supplement: Supplementary file 14 — Figure S7. The positive selection signatures in the codons of Rhsi-DAB1 β1 region were represented based on the mean ω weighted by the posterior probabilities under the models, M2a (a) and M8 (b). (DOCX 78 kb) [file 12863_2019_775_MOESM14_ESM.docx]

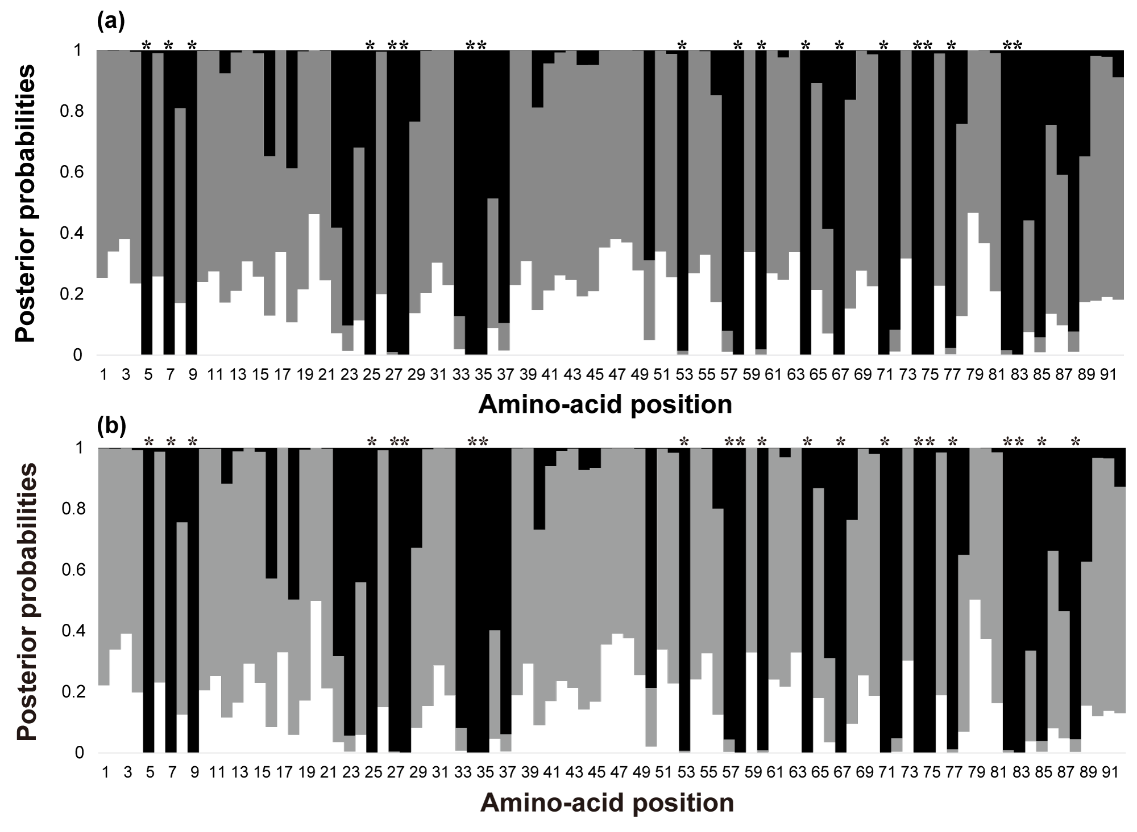

Supplement: Supplementary file 15 — Figure S8. The signature of positive selection was tested with the posterior probability for each codon in Rhsi-DAB1 β1 region under the models, M2a (a) and M8 (b). (DOCX 130 kb) [file 12863_2019_775_MOESM15_ESM.docx]

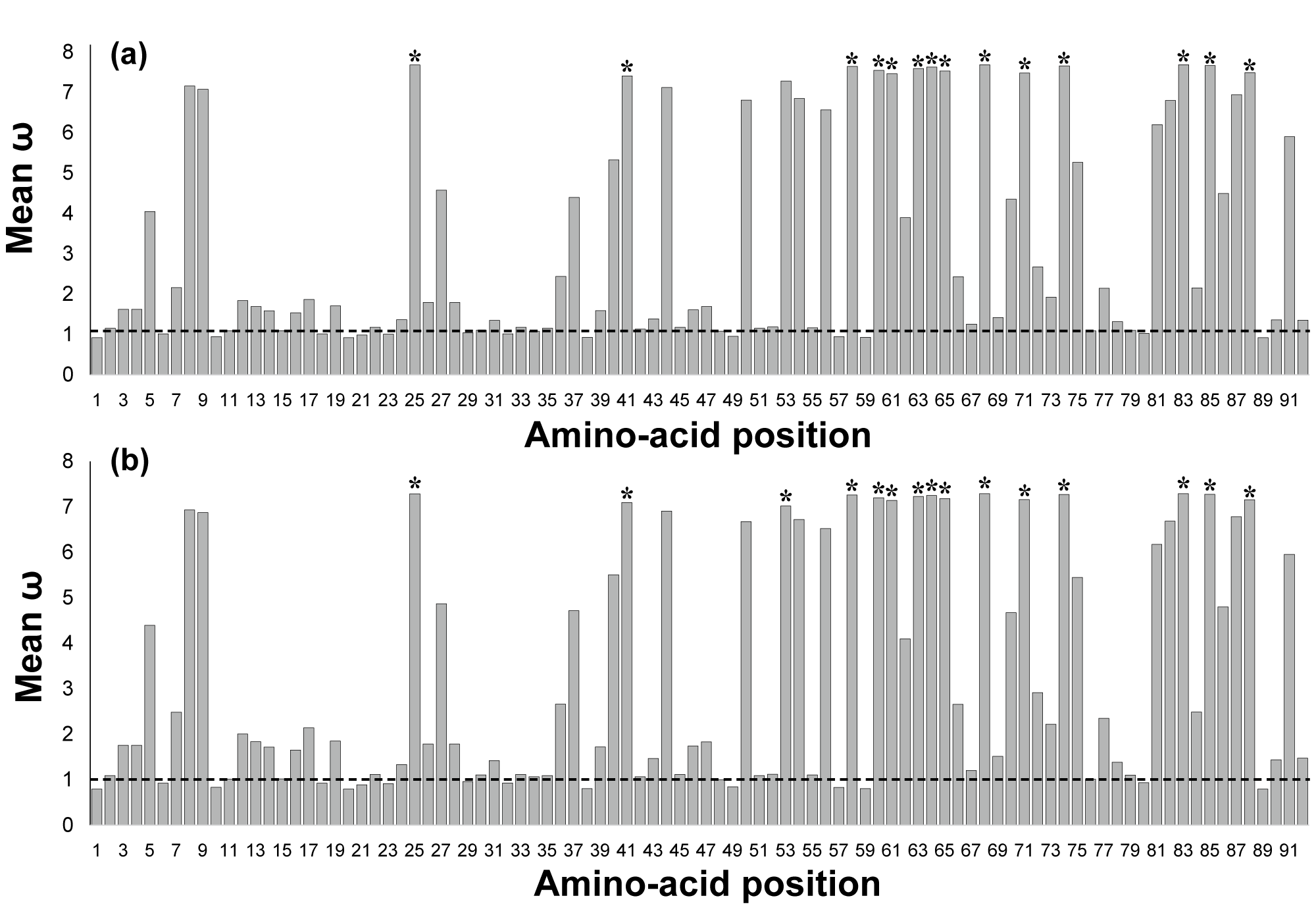

Supplement: Supplementary file 16 — Figure S9. The positive selection signatures in the codons of Rhsi-DAB3 β1 region were represented based on the mean ω weighted by the posterior probabilities under the models, M2a (a) and M8 (b). (DOCX 75 kb) [file 12863_2019_775_MOESM16_ESM.docx]

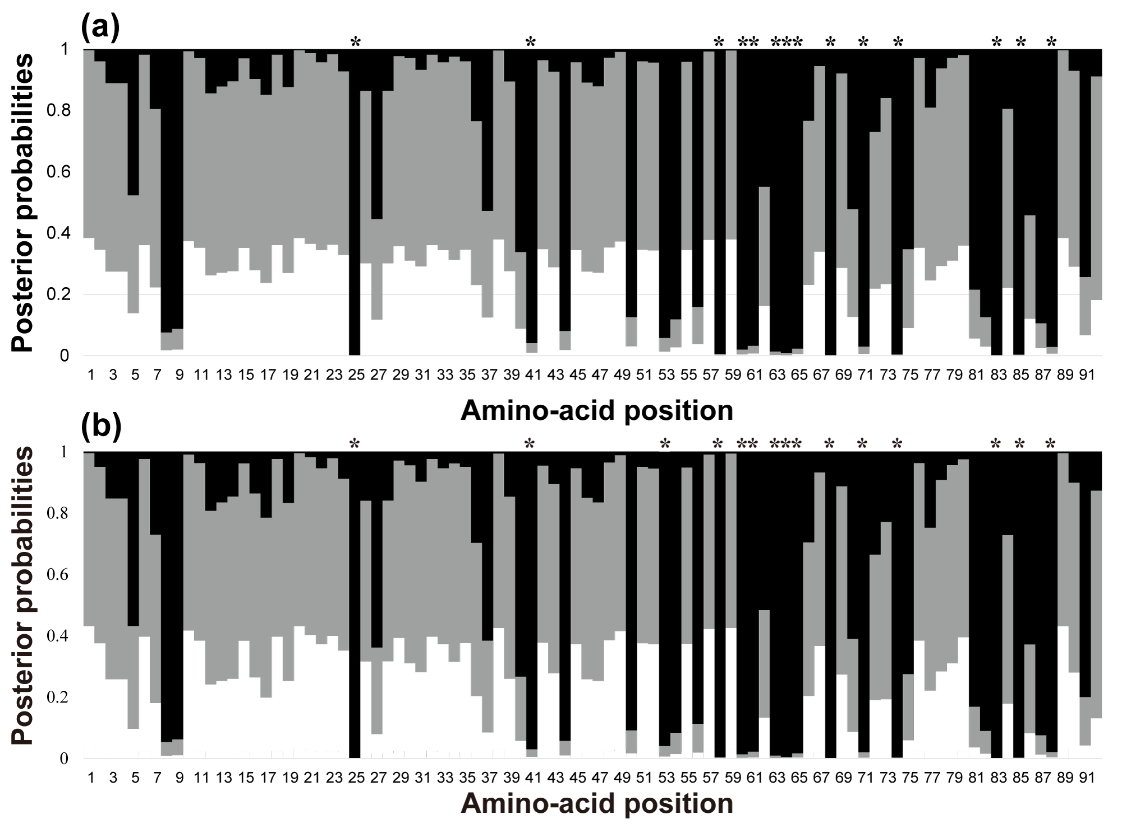

Supplement: Supplementary file 17 — Figure S10. The signature of positive selection was tested with the posterior probability for each codon in Rhsi-DAB3 β1 region under the models, M2a (a) and M8 (b). (DOCX 131 kb) [file 12863_2019_775_MOESM17_ESM.docx]

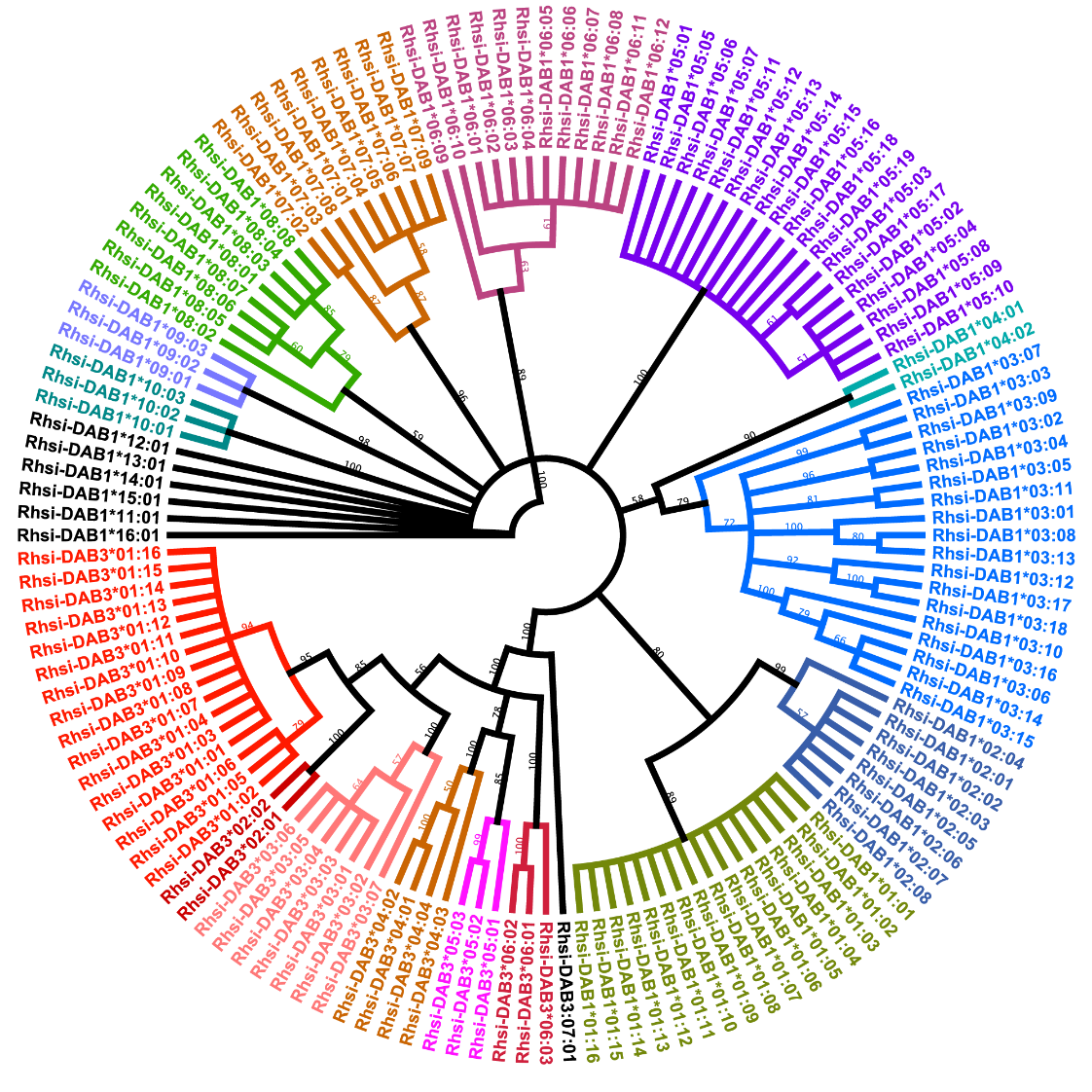

Supplement: Supplementary file 18 — Figure S11. Phylogenetic relationship among Rhsi-DAB β1 alleles reconstructed based on NJ tree analysis. (DOCX 696 kb) [file 12863_2019_775_MOESM18_ESM.docx]
