# Supplementary material for: Polymorphism of MHC class IIB in an acheilognathid species, Rhodeus sinensis shaped by historical selection and recombination
Source: BMC Genet. 2019 Sep 13;20:74. doi: 10.1186/s12863-019-0775-3 (PMC6743125; doi:10.1186/s12863-019-0775-3)
Supplement: Supplementary file 19 — Supplementary data. All sequences (in fasta format) reported in this study. (DOCX 25 kb) [file 12863_2019_775_MOESM19_ESM.docx]

**Supplementary data.** All sequences (in fasta format) reported in this study

>Rhsi-DAB1*01:01

CTGATGCTGTCTGCTTTCACTGGAGCA-----------------------------------------------------------------------------------------------------------------------------------------------------------------------------------------------------------------------GCTCATGGATACTACTGGTCTATGTGGTCTAAATGCATCCACAGCTCCCGGGATTTCAGCGACATGGTGTTCATTGATAACTATATCTTCAATAAAGATGTGTTCATACAGTCCAACAGCACTGTGGGAGAGTATGTGGGGTACACTGAACATGGAGTATATAATGCACGGAATTGGAACAACGATCCCAACATTCTGCAGCAAGAGAGAGCTGAGGTGGAGAGATACTGCAAATATAATGCTGAAATCAGACAGGCAGCTATCGCC---GATAAAACA------------------------------------------------------------------------------------------------------------------------------------------------------------------------------------------------------------------------------------------------------------------------------------------------------------------------------------------------------------------------------------------------GTGGCACCAAAGGTCAAGCTCAGTTCAATGATGCAGGCCAGCGGCAGTCATCCTGCTGTACTGATGTGCAGCGCTCACCGCTTCTACCCACACTGGATCAAAGTGTCCTGGATGAGGGATGGTAAAGTTGTGAAGACTGACGTGACCTCAACTGAGGAGATGCCTAACGGAGACTGGTACTACCAGATTCACTCGCACCTGGAATACACTCCCAAATCTGGAGAGAAGATCTCCTGTGTGGTGGATCACGCCGGCTTAACTAAATCCATCATCGTAGACTGG-----------------------------------------------------------------------------------------------------------------------------------------------------------------------GATCCCGCTATGCCAGAGTCTGACAGGAATAAAATCGCCATCGGAGCGTCTGGTCTGGTGCTGGGGATCATCATAGCAGCTGCTGGACTCATTTATTACAAGAAGAAATCAACAGGGAGGATCCTGGTACCAAACTGA

>Rhsi-DAB1*01:02

CTGATGCTGTCGGCTTTCACTGGAGCA-----------------------------------------------------------------------------------------------------------------------------------------------------------------------------------------------------------------------GCTCATGGATACTACTGGTCTATGTGGTCTAAATGCATCCACAGCTCCCGGGATTTCAGCGACATGGTGTTCATTGATAACTATATCTTCAATAAAGATGTGTTCATACAGTTCAGCAGCACTGTGGGAGAGTATGTGGGGTACACTGAACATGGAGTATATAATGCACGGAATTGGAACAACGATCCCAACATTCTGCAGCAAGAGAGAGCTGAGGTGGAGAGATACTGCAAATATAATGCTGAAATCAGACAGGCAGCTATCGCT---GATAAAACA------------------------------------------------------------------------------------------------------------------------------------------------------------------------------------------------------------------------------------------------------------------------------------------------------------------------------------------------------------------------------------------------GTGGCACCAAAGGTCAAGCTCAGTTCAATGATGCAGGCCAGCGGCAGTCATCCTGCTGTACTGATGTGCAGCGCTTACCGCTTCTACCCACACTGGATCAAAGTGTCCTGGATGAGGGATGGTAAAGTTGTGAAGACTGACGTGACCTCAACTGAGGAGATGCCTAACGGAGACTGGTACTACCAGATTCACTCGCACCTGGAATACACTCCCAAATCTGGAGAGAAGATCTCCTGTGTGGTGGATCACGCCGGCTTAACTAAATCCATCATCGTAGACTGG-----------------------------------------------------------------------------------------------------------------------------------------------------------------------GATCCCGCTATGCCAGAGTCTGACAGGAATAAAATCGCCATCGGAGCGTCTGGTCTGGTGCTGGGGATCATCATAGCAGCTGCTGGACTCATTTATTACAAGAAGAAATCAACAGGGAGGATCCTGGTACCAAA

>Rhsi-DAB1*01:03

CTGATGCTGTCTGCTTTCACTGGAGCA-----------------------------------------------------------------------------------------------------------------------------------------------------------------------------------------------------------------------GCTCATGGATACTACTGGTCTATGTGGTCTAAATGCATCCACAGCTCCCGGGATTTCAGCGACATGGTGTTCATTGATAACTATATCTTCAGTAAAGATGTGTTCATACAGTTCAACAGCACTGTGGGAGAGTATGTGGGGTACACTGAACATGGAGTATATAATGCACGGAATTGGAACAACGATCCCAACATTCTGCAGCAAGAGAGAGCTGAGGTGGAGAGATACTGCAAATATAATGCTGAAATCAGACAGGCAGCTATCGCT---GATAAAACA------------------------------------------------------------------------------------------------------------------------------------------------------------------------------------------------------------------------------------------------------------------------------------------------------------------------------------------------------------------------------------------------GTGGCACCAGAGGTCAAGCTCAA

>Rhsi-DAB1*01:04

CTGATGCTGTCTGCTTTCACTGGAGCA-----------------------------------------------------------------------------------------------------------------------------------------------------------------------------------------------------------------------GCTCATGGATACTACTGGTCTATGTGGTCTAAATGCATCCACAGCTCCCGGGATTTCAACGACATGGTGTTCATTGATAACTATATCTTCAATAAAGATGTGTTCATACAGTTCAACAGCACTGTGGGAGAGTATGTGGGGTACACTGAACATGGAGTATATAATGCACGGAATTGGAACAACGATCCCAACATTCTGCAGCAAGAGAGAGCTGAGGTGGAGAGATACTGCAAATATAATGCTGAAATCAGACAGGCAGCTATCGCT---GATAAAACA------------------------------------------------------------------------------------------------------------------------------------------------------------------------------------------------------------------------------------------------------------------------------------------------------------------------------------------------------------------------------------------------GTGGCACCAAAGGTCAAGCTCAGTTCAATGATGCAGGCCAGCGGCAGTCATCCTGCTGTACTGATGTGCAGCGCTTACCGCTTCTACCCACACTGGATCAAAGTGTCCTGGATGAGGGATGGTAAAGTTGTGAAGACTGACGTGACCTCAACTGAGGAGATGCCTAACGGAGACTGGTACTACCAGATTCACTCGCACCTGGAATACACTCCCAAATCTGGAGAGAAGATCTCCTGTGTGGTGGATCACGCCGGCTTAACTAAATCCATCATCGTAGACTGG-----------------------------------------------------------------------------------------------------------------------------------------------------------------------GATCCCGCTATGCCAGAGTCTGACAGGAATAAAATCGCCATCGGAGCGTCTGGTCTGGTGCTGGGGATCATCATAGCAGCTGCTGGACTCATTTATTACAAGAAGAAATCAACA

>Rhsi-DAB1*01:05

CTGATGCTGTCGGCATTCACCGGAGCA-----------------------------------------------------------------------------------------------------------------------------------------------------------------------------------------------------------------------GCTCATGGATACTACTGGTCTATGTGGTCTAAATGCATCCACAGCTCCCGAGATTTCAGCGACATGGTGTTCATTGATAGCTATATCTTCAATAAAGATGTGTTCATACAGTTCAACAGCACTGTGGGAGAGTATGTGGGGTACACTGAACATGGAGTATATAATGCACGGAATTGGAACAACGATCCCAACATTCTGCAGCAAGAGAGAGCTGAGGTGGAGAGATACTGCAAATATAATGCTGAAATCAGACAGGCAGCTATCGCT---GATAAAACA------------------------------------------------------------------------------------------------------------------------------------------------------------------------------------------------------------------------------------------------------------------------------------------------------------------------------------------------------------------------------------------------GTGGCACCAAAGGTCAAGCTCAGTTCAATGATGCAGGCCAGCGGCAGTCATCCTGCTGTACTGATGTGCAGCGCTTACCGCTTCTACCCACACTGGATCAAAGTGTCCTGGATGAGGGATGGTAAAGTTGTGAAGACTGACGTGACCTCAACTGAGGAGATGCCTAACGGAGACTGGTACTACCAGATTCACTCGCACCTGGAATACACTCCCAAATCTGGAGAGAAGATCTCCTGTGTGGTGGATCACGCCGGCTTAACTAAATCCATCATCGTAGACTGG-----------------------------------------------------------------------------------------------------------------------------------------------------------------------GATCCCGCTATGCCAGAGTCTGACAGGAATAAAATCGCCATCGGAGCGTCTGGTCTGGTGCTGGGGATCATCATAGCAGCTGCTGGACTCATTTATTACAAGAAGAAATCAACAGGGAGGATCCTGGTACCAAA

>Rhsi-DAB1*01:06

CTGATGCTGTCTGCATTTACCGGAGCA-----------------------------------------------------------------------------------------------------------------------------------------------------------------------------------------------------------------------GCTCATGGATACTACTGGTCTATGTGGTCTAAATGCATCCACAGCTCCCGGGATTTCAGCGACATGGTGTTCATTGATAACTATATCTCCAATAAAGATGTGTTCATACAGTTCAACAGCACTGTGGGAGAGTATGTGGGGTACACTGAACATGGAGTATATAATGCACGGAATTGGAACAACGATCCCAACATTCTGCAGCAAGAGAGAGCTGAGGTGGAGAGATACTGCAAATATAATGCTGAAATCAGACAGGCAGCTATCGCT---GATAAAACA------------------------------------------------------------------------------------------------------------------------------------------------------------------------------------------------------------------------------------------------------------------------------------------------------------------------------------------------------------------------------------------------GTGGCACCAAAGGTCAAGCTCAGTTCAATGATGCAGGCCAGCGGCAGTCATCCTGCTGTACTGATGTGCAGCGCTTACCGCTTCTACCCACACTGGATCAAAGTGTCCTGGATGAGGGATGGTAAAGTTGTGAAGACTGACGTGACCTCAACTGAGGAGATGCCTAACGGAGACTGGTACTACCAGATTCACTCGCACCTGGAATACACTCCCAAATCTGGAGAGAAGATCTCCTGTGTGGTGGATCACGCCGGCTTAACTAAATCCATCATCGTAGACTGG-----------------------------------------------------------------------------------------------------------------------------------------------------------------------GATCCCGCTATGCCAGAGTCTGACAGGAATAAAATCGCCATCGGAGCGTCTGGTCTGGTGCTGGGGATCATCATAGCAGCTGCTGGACTCATTTATTACAAGAAGAAATCAACAGGGAGGATCCTGGTACCAAA

>Rhsi-DAB1*01:07

CTGATGCTGTCTGCATTCACTGGAGCA-----------------------------------------------------------------------------------------------------------------------------------------------------------------------------------------------------------------------GCTCATGGATACTACTGGTCTATGTGGTCTAAATGCATCCACAGCTCCCGGGATTTCAGCGACATGGTGTTCATTGATAACTATGTCTTCAATAAAGATGTGTTCATACAGTTCAACAGCACTGTGGGAGAGTATGTGGGATACACTGAACATGGAGTATATAATGCACGGAATTGGAACAACGATCCCAACATTCTGCAGCAAGAGAGAGCTGAGGTGGAGAGATACTGCAAATATAATGCTGAAATCAGACAGGCAGCTATCGCT---GATAAAACA------------------------------------------------------------------------------------------------------------------------------------------------------------------------------------------------------------------------------------------------------------------------------------------------------------------------------------------------------------------------------------------------GTGGCACCAAAGGTCAAGCTCAGTCCAATGATGCAGGCCAGCGGCAGTCATCCTGCTGTACTGATGTGCAGCGCTTACCGCTTCTACCCACACTGGATCAAAGTGTCCTGGATGAGGGATGGTAAAGTTGTGAAGACTGACGTGACCTCAACTGAGGAGATGCCTAACGGAGACTGGTACTACCAGATTCACTCGCACCTGGAATACACTCCCAAATCTGGAGAGAAGATCTCCTGTGTGGTGGATCACGCCGGCTTAACTAAATCCATCATCGTAGACTGG-----------------------------------------------------------------------------------------------------------------------------------------------------------------------GATCCCGCTATGCCAGAGTCTGACAGGAATAAAATCGCCATCGGAGCGTCTGGTCTGGTGCTGGGGATCATCATAGCAGCTGCTGGACTCATTTATTACAAGAAGAAATCAACAGGGAGGATCCTGGTACCAAA

>Rhsi-DAB1*01:08

CTGATGCTGTCTGCATTTACTGGAGCA-----------------------------------------------------------------------------------------------------------------------------------------------------------------------------------------------------------------------GCTCATGGATACTACTGGTCTATGTGGTCTAAATGCATCCACAGCTCCCGGGATTTCAGCGACATGGTGTTCATTGATAACTATATCTTCAATAAAGATGTGCTCATACAGTTCAACAGCACTGTGGGAGAGTATGTGGGGTACACTGAACATGGAGTATATAATGCACGGAATTGGAACAACGATCCCAACATTCTGCAGCAAGAGAGAGCTGAGGTGGAGAGATACTGCAAATATAATGCTGAAATCAGACAGGCAGCTATCGCT---GATAAAACA------------------------------------------------------------------------------------------------------------------------------------------------------------------------------------------------------------------------------------------------------------------------------------------------------------------------------------------------------------------------------------------------GTGGCACCAAAGGTCAAGCTCAGTTCAATGATGCAGGCCAGCGGCAGTCATCCTGCTGTACTGATGTGCAGCGCTTACCGCTTCTACCCACACTGGATCAAAGTGTCCTGGATGAGGGATGGTAAAGTTGTGAAGACTGACGTGACCTCAACTGAGGAGATGCCTAACGGAGACTGGTACTACCAGATTCACTCGCACCTGGAATACACTCCCAAATCTGGAGAGAAGATCTCCTGTGTGGTGGATCACGCCGGCTTAACTAAATCCATCATCGTAGACTGG-----------------------------------------------------------------------------------------------------------------------------------------------------------------------GATCCCGCTATGCCAGAGTCTGACAGGAATAAAATCGCCATCGGAGCGTCTGGTCTGGTGCTGGGGATCATCATAGCAGCTGCTGGACTCATTTATTACAAGAAGAAATCAACAGGGAGGATCCTGGTACCAAA

>Rhsi-DAB1*01:09

CTGATGCTGTCTGCTTTCACTGGAGCA-----------------------------------------------------------------------------------------------------------------------------------------------------------------------------------------------------------------------GCTCATGGATACTACTGGTCTATGTGGTCTAAATGCATCCACAGCTCCCGGGATTTCAGTGACATGGTGTTCATTGATAACTATATCTTCAATAAAGATGTGTTCATACAGTTCAACAGCACTGTGGGAGAGTATGTGGGGTACACTGAACATGGAGTATATAATGCACGGAATTGGAACAACGATCCCAACATTCTGCAGCAAGAGAGAGCTGAGGTGGAGAGATACTGCAAATATAATGCTGAAATCAGACAGGCAGCTATCGCT---GATAAAACA------------------------------------------------------------------------------------------------------------------------------------------------------------------------------------------------------------------------------------------------------------------------------------------------------------------------------------------------------------------------------------------------GTGGCACCAAAGGTCAAGCTCAGTTCAATGATGCAGGCCAGCGGCAGTCATCCTGCTGTACTGATGTGCAGCGCTTACCGCTTCTACCCACACTGGATCAAAGTGTCCTGGATGAGGGATGGTAAAGTTGTGAAGACTGACGTGACCTCAACTGAGGAGATGCCTAACGGAGACTGGCACTACCAAATTCACTCGCACCTGGAATACACTCCCAAATCTGGAGAGAAGATCTCCTGTGTGGTGGATCACGCCGGCTTAACTAAATCCATCATCGTAGACTGG-----------------------------------------------------------------------------------------------------------------------------------------------------------------------GATCCCGCTATGCCAGAGCCTGACAGGAATAAAATCGCCATCGGAGCGTCTGGTCTGGTGCTGGGGATCATCATAGTAGCTGCTGGACTCATTTATTACAAGAAGAAATCAACAGGGAGGATCCTGGTACCAAACTGA

>Rhsi-DAB1*01:10

CTGATGCTGTCTGCTTTTACTGGAGCA-----------------------------------------------------------------------------------------------------------------------------------------------------------------------------------------------------------------------GCTCATGGATACTACTGGTCTATGTGGTCTAAATGCATCCACAGCTCCTGGGATTTCAGCGACATGGTGTTCATTGATAACTATATCTTCAATAAAGATGTGTTCATACAGTTCAACAGCACTGTGGGAGAGTATGTGGGGTACACTGAACATGGAGTATATAATGCACGGAATTGGAACAACGATCCCAACATTCTGCAGCAAGAGAGAGCTGAGGTGGAGAGATACTGCAAATATAATGCTGAAATCAGACAGGCAGCTATCGCT---GATAAAACA------------------------------------------------------------------------------------------------------------------------------------------------------------------------------------------------------------------------------------------------------------------------------------------------------------------------------------------------------------------------------------------------GTGGCACCAAAGGTCAAGCTCAGTTCAATGATGCAGGCCAGCGGCAGTCATCCTGCTGTACTGATGTGCAGCGCTTACCGCTTCTACCCACACTGGATCAAAGTGTCCTGGATGAGGGATGGTAAAGTTGTGAAGACTGACGTGACCTCAACTGAGGAGATGCCTAACGGAGACTGGTACTACCAGATTCACTCGCACCTGGAATACACTCCCAAATCTGGAGAGAAGATCTCCTGTGTGGTGGATCACGCCGGCTTAACTAAATCCATCATCGTAGACTGG-----------------------------------------------------------------------------------------------------------------------------------------------------------------------GATCCCGCTATGCCAGAGTCTGACAGGAATAAAATCGCCATCGGAGCGTCTGGTCTGGTGCTGGGGATCATCATAGCAGCTGCTGGACTCATTTATTACAAGAAGAAATCAACAGGGAGGATCCTGGTACCAAA

>Rhsi-DAB1*01:11

CTGATGCTGTCTGCTTTCACTGGAGCA-----------------------------------------------------------------------------------------------------------------------------------------------------------------------------------------------------------------------GCTCATGGATACTACTGGTCTATGTGGTCTAAATGCATCCACAGCTCCCGGGATTTCAGCGACATGGTGTTCATTGATAACTATATCTTCAATAAAGATGTGTTCATACAGTTCAACAGCGCTGTGGGAGAGTATGTGGGGTACACTGAACATGGAGTATATAATGCACGGAATTGGAACAACGATCCCAACATTCTGCAGCAAGAGAGAGCTGAGGTGGAGAGATACTGCAAATATAATGCTGAAATCAGACAGGCAGCTATCGCT---GATAAAACA------------------------------------------------------------------------------------------------------------------------------------------------------------------------------------------------------------------------------------------------------------------------------------------------------------------------------------------------------------------------------------------------GTGGCACCAAAGGTCAAGCTCAGTTCAATGATGCAGGCCAGCGGCAGTCATCCTGCTGTACTGATGTGCAGCGCTTACCGCTTCTACCCACACTGGATCAAAGTGTCCTGGATGAGGGATGGTAAAGTTGTGAAGACTGACGTGACCTCAACTGAGGAGATGCCTAACGGAGACTGGA

>Rhsi-DAB1*01:12

CTGATGCTGTCTGCTTTCACTGGAGCA-----------------------------------------------------------------------------------------------------------------------------------------------------------------------------------------------------------------------GCTCATGGATACTACTGGTCTATGTGGTCTAAATGCATCCACGGCTCCCGGGATTTCAGCGACATGGTGTTCATTGATAACTATATCTTCAATAAAGATGTGTTCATACAGTTCAACAGCACTGTGGGAGAGTATGTGGGGTACACTGAACATGGAGTATATAATGCACGGAATCGGAACAACGATCCCAACATTCTGCAGCAAGAGAGAGCTGAGGTGGAGAGATACTGCAAATATAATGCTGAAATCAGACAGGCAGCTATCGCT---GATAAAACA------------------------------------------------------------------------------------------------------------------------------------------------------------------------------------------------------------------------------------------------------------------------------------------------------------------------------------------------------------------------------------------------GTGGCACCAGAGGTCAAGCTCAA

>Rhsi-DAB1*01:13

CTGATGCTGTCTGCTTTCACTGGAGCA-----------------------------------------------------------------------------------------------------------------------------------------------------------------------------------------------------------------------GCTCATGGATACTACTGGTCTATGTGGTCTAAATGCATCCACAGCTCCCGGGATTTCAGCGACATGGTGTTCATTGATAACTATATCCTCAATAAAGATGTGTTCATACAGTTCAACAGCACTGTGGGAGAGTATGTGGGGTATACTGAACATGGAGTATATAATGCACGGAATTGGAGCAACGACCCCAACATTCTGCAGCAAGAGAGAGCTGAGGTGGAGAGATACTGCAAATATAATGCTGAAATCAGACAGGCAGCTATCGCT---GATAAAACA------------------------------------------------------------------------------------------------------------------------------------------------------------------------------------------------------------------------------------------------------------------------------------------------------------------------------------------------------------------------------------------------GTGGCACCAAAGGTCAAGCTCAGTTCAATGATGCAGGCCAGCGGCAGTCATCCTGCTGTACTGATGTGCAGCGCTTACCGCTTCTACCCACACTGGATCAAAGTGTCCTGGATGAGGGATGGTAAAGTTGTGAAGACTGACGTGACCTCAACTGAGGAGATGCCTAACGGAGACTGGTACTACCAGATTCACTCGCACCTGGAATACACTCCCAAATCTGGAGAGAAGATCTCCTGTGTGGTGGATCACGCCGGCTTAACTAAATCCATCATCGTAGACTGG-----------------------------------------------------------------------------------------------------------------------------------------------------------------------GATCCCGCTATGCCAGAGTCTGACAGGAATAAAATCGCCATCGGAGCGTCTGGTCTGGTGCTGGGGATCATCATAGCAGCTGCTGGACTCATTTATTACAAGAAGAAATCAACAGGGAGGATCCTGGTACCAAACTGA

>Rhsi-DAB1*01:14

CTGATGCTGTCTGCTTTCACTGGAGCA-----------------------------------------------------------------------------------------------------------------------------------------------------------------------------------------------------------------------GCTCATGGATACTACTGGTCTATGTGGTCTAAATGCATCCACAGCTCCCGGGATTTCAGCGACATGGTGTTCATTGATAACTATATCTTCAATAAAGTTGTGTTCATACAGTTCAACAGCACTGTGGGAGAGTATGTGGGGTACACTGAACATGGAGTATATAATGCACGGAATTGGAACAACGATCCCAACATTCTGCAGCAAGAGAGAGCTGAGGTGGAGAGATACTGCAAATATAATGCTGAAATCAGACAGGCAGCTATCGCT---GATAAAACA------------------------------------------------------------------------------------------------------------------------------------------------------------------------------------------------------------------------------------------------------------------------------------------------------------------------------------------------------------------------------------------------GTGGCACCAAAGGTCAAGCTCAGTTCAATGATGCAGGCCAGCGGCAGTCATCCTGCTGTACTGATGTGCAGCGCTTACCGCTTCTACCCACACTGGATCAAAGTGTCCTGGATGAGGGATGGTAAAGTTGTGAAGACTGACGTGACCTCAACTGAGGAGATGCCTAACGGAGACTGGTACTACCAGATTCACTCGCACCTGGAATACACTCCCAAATCTGGAGAGAAGATCTCCTGTGTGGTGGATCACGCCGGCTTAACTAAATCCATCATCGTAGACTGG-----------------------------------------------------------------------------------------------------------------------------------------------------------------------GATCCCGCTATGCCAGAGTCTGACAGGAATAAAATCGCCATCGGAGCGTCTGGTCTGGTGCTGGGGATCATCATAGCAGCTGCTGGACTCATTTATTACAAGAAGAAATCAACAGGGAGGATCCTGGTACCAAACTGA

>Rhsi-DAB1*01:15

CTGATGCTGTCTGCTTTCACTGGAGCA-----------------------------------------------------------------------------------------------------------------------------------------------------------------------------------------------------------------------GCTCATGGATACCACTGGTCTATGTGGTCTAAATGCATCCACAGCTCCCGGGATTTCAGCGACGTGGTGTTCATTGATAACTATATCTTCAATAAAGATGTGTTCATACAGTTCAACAGCACTGTGGGAGAGTATGTGGGGTACACTGAACATGGAGTATATAATGCACGGAATTGGAACAACGATCCCAACATTCTGCAGCAAGAGAGAGCTGAGGTGGAGAGATACTGCAAATATAATGCTGAAATCAGACAGGCAGCTATCGCT---GATAAAACA------------------------------------------------------------------------------------------------------------------------------------------------------------------------------------------------------------------------------------------------------------------------------------------------------------------------------------------------------------------------------------------------GTGGCACCAAAGGTCAAGCTCAGTTCAATGATGCAGGCCAGCGGCAGTCATCCTGCTGTACTGATGTGCAGCGCTTACCGCTTCTACCCACACTGGATCAAAGTGTCCTGGATGAGGGATGGTAAAGTTGTGAAGACTGACGTGACCTCAACTGAGGAGATGCCTAACGGAGACTGGTACTACCAGATTCACTCGCACCTGGAATACACTCCCAAATCTGGAGAGAAGATCTCCTGTGTGGTGGATCACGCCGGCTTAACTAAATCCATCATCGTAGACTGG-----------------------------------------------------------------------------------------------------------------------------------------------------------------------GATCCCGCTATGCCAGAGTCTGACAGGAATAAAATCGCCATCGGAGCGTCTGGTCTGGTGCTGGGGATCATCATAGCAGCTGCTGGACTCATTTATTACAAGAAGAAATCAACAGGGAGGATCCTGGTACCAAACTGA

>Rhsi-DAB1*01:16

CTGATGCTGTCTGCTTTCACTGGAGCA-----------------------------------------------------------------------------------------------------------------------------------------------------------------------------------------------------------------------GCTCATGGATACTACTGGTCTATGTGGTCTAAATGCATCCACAGCTCCCGGGATTTCAGCGACATGGTGTTCATTGATAACTATATCTTCAATAAAGATGTGTTCATACAGTTCGACAGCACTGTGGGAGAGTATGTGGGGTACACTGAACATGGAGTATATAATGCACGGAATTGGAACAACGATCCCAACATTCTGCAGCAAGAGAGAGCTGAGGTGGAGAGATACTGCAAATATAATGCTGAAATCAGACAGGCAGCTATCGCT---GATAAAACA------------------------------------------------------------------------------------------------------------------------------------------------------------------------------------------------------------------------------------------------------------------------------------------------------------------------------------------------------------------------------------------------GTGGCACCAAAGGTCAAGCTCAGTTCAATGATGCAGGCCAGCGGCAGTCATCCTGCTGTACTGATGTGCAGCGCTTACCGCTTCTACCCACACTGGATCAAAGTGTCCTGGATGAGGGATGGTAAAGTTGTGAAGACTGACGTGACCTCAACTGAGGAGATGCCTAACGGAGACTGGTACTACCAGATTCACTCGCACCTGGAATACACTCCCAAATCTGGAGAGAAGATCTCCTGTGTGGTGGATCACGCCGGCTTAACTAAATCCATCATCGTAGACTGG-----------------------------------------------------------------------------------------------------------------------------------------------------------------------GATCCCGCTATGCCAGAGTCTGACAGGAATAAAATCGCCATCGGAGCGTCTGGTCTGGTGCTGGGGATCATCATAGCAGCTGCTGGACTCATTTATTACAAGAAGAAATCAACAGGGAGGATCCTGGTACCAAACTGA

>Rhsi-DAB1*02:01

CTGATGCTGTCTGCTTTCACCGGAGCA-----------------------------------------------------------------------------------------------------------------------------------------------------------------------------------------------------------------------GCTCATGGATACTACTGGTCTATGTGGTCTAAATGCATCCACAGCTCCCGGGATTTCAGCGACATGGTGTTCATTGATAACTATATCTTCAATAAAGATGCGTTCATACAGTTCAACAGCACTGTGGGAGAGTATGTGGGGTACACTGAACATGGAGTATATAATGCACGGAATTGGAACAGCGATCCCAACATTCTGCAGCAAGAGAGAGCTGAGTTGGAGAGATACTGCAAATATAATGCTGAACTCTATCAGGCAGCTATCGCT---GATAAAACA------------------------------------------------------------------------------------------------------------------------------------------------------------------------------------------------------------------------------------------------------------------------------------------------------------------------------------------------------------------------------------------------GTGGCACCAAAGGTCAAGCTCAGTTCAGTGATGCAGGCCAGCGGCAGTCATCCTGCTGTACTGATGTGCAGCGCTTACCGCTTCTACCCACACTGGATCAAAGTGTCCTGGATGAGGGATGGTAAAGTTGTGAAGACTGACGTGACCTCAACTGAGGAGATGCCTAACGGAGACTGGTACTATCAGATTCACTCGCACCTGGAATACACTCCCAAATCTGGAGAGAAGATCTCCTGTGTGGTGGATCACGCCGGCTTAACTAAATCCATCATCGTAGACTGG-----------------------------------------------------------------------------------------------------------------------------------------------------------------------GATCCCGCTATGCCAGAGTCTGACAGGAATAAAATCGCCATCGGAGCGTCTGGTCTGGTGCTGGGGATCATCATAGCAGCTGCTGGACTCATTTACTACAAGAAGAAATCAACAGGGAGGATCCTGGTACCAAATC

>Rhsi-DAB1*02:02

CTGATGCTGTCTGCTTTCACTGGAGCA-----------------------------------------------------------------------------------------------------------------------------------------------------------------------------------------------------------------------GCTCATGGATACTACTGGTCTATGTGGTCTAAATGCATCCACAGCTCCCGGGATTCCAGCGACATGGTGTTCATTGATAACTATATCTTCAATAAAGATGTGTTCATACAGTTCAACAGCACTGTGGGAGAGTATGTGGGGTACACTGAACATGGAGTATATAATGCACGGAATTGGAACAGCGATCCCAACATTCTGCAGCAAGAGAGAGCTGAGTTGGAGAGATACTGCAAATATAATGCTGAACTCTATCAGGCAGCTATCGCT---GATAAAACA------------------------------------------------------------------------------------------------------------------------------------------------------------------------------------------------------------------------------------------------------------------------------------------------------------------------------------------------------------------------------------------------GTGGCACCAAAGGTCAAGCTCAGTTCAGTGACGCAGGCCAGCGGCAGTCATCCTGCTGTACTGATGTGCAGCGCTTACCGCTTCTACCCACACTGGATCAAAGTGTCCTGGATGAGGGATGGTAAAGTTGTGAAGACTGACGTGACCTCAACTGAGGAGATGCCTAACGGAGACTGGTACTACCAGATTCACTCGCACCTGGAATACACTCCCAAATCTGGAGAGAAGATCTCCTGTGTGGTGGATCACGCCGGCTTAACTAAATCCATCATCGTAGACTGG-----------------------------------------------------------------------------------------------------------------------------------------------------------------------GATCCCGCTATGCCAGAGCCTGACAGGAATAAAATCGCCATCGGAGCGTCTGGTCTGGTGCTGGGGATCATCATAGCAGCTGCTGGACTCATTTATTACAAGAAGAAATCAACAGGGAGGATCCTGGTACCAAACTGA

>Rhsi-DAB1*02:03

CTGATGCTGTCTGCTTTTACTGGAGCA-----------------------------------------------------------------------------------------------------------------------------------------------------------------------------------------------------------------------GCTCATGGATACTACTGGTCTATGTGGTCTAAATGCATCCACAGCTCCCGGGATTTCAGCGACATGGTGTTCATTGATAACTATATCTTCAATAAAGATGTGTTCATACGGTTCAACAGCACTGTGGGAGAGTATGTGGGGTACACTGAACATGGAGTATATAATGCACGGAATTGGAACAGCGATCCCAACATTCTGCAGCAAGAGAGAGCTGAGTTGGAGAGATACTGCAAATATAATGCTGAACTCTATCAGGCAGCTATCGCT---GATAAAACA------------------------------------------------------------------------------------------------------------------------------------------------------------------------------------------------------------------------------------------------------------------------------------------------------------------------------------------------------------------------------------------------GTGGCACCAAAGGTCAAGCTCAGTTCAGTGATGCAGGCCAGCGGCAGTCATCCTGCTGTACTGATGTGCAGCGCTTACCGCTTCTACCCACACTGGACCAAAGTGTCCTGGATGAGGGATGGTAAAGTTGTGAAGACTGACGTGACCTCAACTGAGGAGATGCCTAACGGAGACTGGTACTATCAGATTCACTCGCACCTGGAATACACTCCCAAATCTGGAGAGAAGATCTCCTGTGTGGTGGATCACGCCGGCTTAACTAAATCCATCATCGTAGACTGG-----------------------------------------------------------------------------------------------------------------------------------------------------------------------GATCCCGCTATGCCAGAGTCTGACAGGAATAAAATCGCCATCGGAGCGTCTGGTCTGGTGCTGGGGATCATCATAGCAGCTGCTGGACTCATTTACTACAAGAAGAAATCAACAGGGAGGATCCTGGTACCAAATC

>Rhsi-DAB1*02:04

CTGATGCTGTCTGCTTTCACTGGAGCA-----------------------------------------------------------------------------------------------------------------------------------------------------------------------------------------------------------------------GCTCATGGATACTACTGGTCTATGTGGTCTAAATGCATCCACAGCTCCCGGGATTTCAGCGACATGGTGTTCATTGATAACTATATCTTCAATAAAGGTGTGTTCATACAGTTCAACAGCACTGTGGGAGAGTATGTGGGGTACACTGAACATGGAGTATATAATGCACGGAATTGGAACAGCGATCCCAACATTCTGCAGCAAGAGAGAGCTGAGTTGGAGAGATACTGCAAATATAATGCTGAACTCTATCAGGCAGCTATCGCT---GATAAAACA------------------------------------------------------------------------------------------------------------------------------------------------------------------------------------------------------------------------------------------------------------------------------------------------------------------------------------------------------------------------------------------------GTGGCACCAAAGGTCAAGCTCAGTTCAGTGATGCAGGCCAGCGGCAGTCATCCTGCTGTACTGATGTGCAGCGCTTACCGCTTCTACCCACACTGGATCAAAGTGTCCTGGATGAGGGATGGTAAAGTTGTGAAGACTGACGTGACCTCAACTGAGGAGATGCCTAACGGAGACTGGTACTATCAGATTCACTCGCACCTGGAATACACTCCCAAATCTGGAGAGAAGATCTCCTGTGTGGTGGATCACGCCGGCTTAACTAAATCCATCATCGTAGACTGG-----------------------------------------------------------------------------------------------------------------------------------------------------------------------GATCCCGCTATGCCAGAGTCTGACAGGAATAAAATCGCCATCGGAGCGTCTGGTCTGGTGCTGGGGATCATCATAGCAGCTGCTGGACTCATTTACTGCAAGAAGAAATCAACAGGGAGGATCCTGGTACCAAACTGA

>Rhsi-DAB1*02:05

CTGATGCTGTCTGCTTTCACTGGAGCA---------------------------------------------------------------------------------------------------------------------------------------------------------------------------------GGTAATACAATCAAG-----------------------ACTCATGGATACTACTGGTCTATGTGGTCTAAATGCATCCACAGCTCCCGGGATTTCAGCGACATGGGGTTCATTGATAACTATATCTTCAATAAAGATGTGTTCATACAGTTCAACAGCACTGTGGGAGAGTATGTGGGGTACACTGAACATGGAGTATATAATGCACGGAATTGGAACAGCGATCCCAACATTCTGCAGCAAGAGAGAGCTGAGTTGGAGAGATACTGCAAATATAATGCTGAACTCTATCAGGCAGCTATCGCT---GATAAAACA------------------------------------------------------------------------------------------------------------------------------------------------------------------------------------------------------------------------------------------------------------------------------------------------------------------------------------------------------------------------------------------------GTGGCACCAAAGGTCAAGCTCAGTTCAGTGATGCAGGCCAGCGGCAGTCATCCTGCTGTACTGATGTGCAGCGCTTACCGCTTCTACCCACACTGGATCAAAGTGTCCTGGATGAGGGATGGTAAAGTTGTGAAGACTGACGCGACCTCAACTGAGGAGATGCCTAACGGAGACTGGTACTATCAGATTCACTCGCACCTGGAATACACTCCCAAATCTGGAGAGAAGATCTCCTGTGTGGTGGATCACGCCGGCTTAACTAAATCCATCATCGTAGACTGG-----------------------------------------------------------------------------------------------------------------------------------------------------------------------GATCCCGCTATGCCAGAGTCTGACAGGAATAAAATCGCCATCGGAGCGTCTGGTCTGGTGCTGGGGATCATCATAGCAGCTGCTGGACTCATTTACTACAAGAAGAAATCAACAGGGAGGATCCTGGTACCAA

>Rhsi-DAB1*02:06

CTGATGCTGTCTGCATTTACTGGAGCA-----------------------------------------------------------------------------------------------------------------------------------------------------------------------------------------------------------------------GCTCATGGATACTACTGGTCTATGTGGTCTAAATGCATCCACAGCTCCCGGGATTTCAGCGACATGGTGTTCACTGATAACTATATCTTCAATAAAGATGTGTTCATACAGTTCAACAGCACTGTGGGAGAGTATGTGGGGTACACTGAACATGGAGTATATAATGCACGGAATTGGAACAGCGATCCCAACATTCTGCAGCAAGAGAGAGCTGAGTTGGAGAGATACTGCAAATATAATGCTGAACTCTATCAGGCAGCTATCGCT---GATAAAACA------------------------------------------------------------------------------------------------------------------------------------------------------------------------------------------------------------------------------------------------------------------------------------------------------------------------------------------------------------------------------------------------GTGGCACCAAAGGTCAAGCTCAGTTCAGTGATGCAGGCCAGCGGCAGTCATCCTGCTGTACTGATATGCAGCGCTTACCGCTTCTACCCACACTGGATCAAAGTGTCCTGGATGAGGGATGGTAAAGTTGTGAAGACTGACGTGACCTCAACTGAGGAGATGCCTAACGGAGACTGGTACTATCAGATTCACTCGCACCTGGAATACACTCCCAAATCTGGAGAGAAGATCTCCTGTGTGGTGGATCACGCCGGCTTAACTAAATCCATCATCGTAGACTGG-----------------------------------------------------------------------------------------------------------------------------------------------------------------------GATCCCGCTATGCCAGAGTCTGACAGGAATAAAATCGCCATCGGAGCGTCTGGTCTGGTGCTGGGGATCATCATAGCAGCTGCTGGACTCATTTACTACAAGAAGAAATCAACAGGGAGGATCCTGGTACCAA

>Rhsi-DAB1*02:07

CTGATGCTGTCTGCTTTTACTGGAACA-----------------------------------------------------------------------------------------------------------------------------------------------------------------------------------------------------------------------GCTCATGGATACTACTGGTCTATGTGGTCTAAATGCATCCACAGCTCCCGGGACTTCAGCGACATGGTGTTCATTGATAACTATATCTTCAATAAAGATGTGTTCATACAGTTCAACAGCACTGTGGGAGAGTATGTGGGGTACACTGAACATGGAGTATATAATGCACGGAATTGGAACAGCGATCCCAACATTCTGCAGCAAGAGAGAGCTGAGTTGGAGAGATACTGCAAATATAATGCTGAACTCTATCAGGCAGCTATCGCT---GATAAAACA------------------------------------------------------------------------------------------------------------------------------------------------------------------------------------------------------------------------------------------------------------------------------------------------------------------------------------------------------------------------------------------------GTGGCACCAAAGGTCAAGCTCAGTTCAGTGATGCAGGCCAGCGGCAGTCATCCTGCTGTACTGATGTGCAGCGCTTACCGCTTCTACCCACACTGGATCAAAGTGTCCTGGATGAGGGATGGTAAAGTCGTGAAGACTGACGTGACCTCAACTGAGGAGATGCCTAACGGAGACTGGTACTATCAGATTCACTCGCACCTGGAATACACTCCCAAATCTGGAGAGAAGATCTCCTGTGTGGTGGATCACGCCGGCTTAACTAAATCCATCATCGTAGACTGG-----------------------------------------------------------------------------------------------------------------------------------------------------------------------GATCCCGCTATGCCAGAGTCTGACAGGAATAAAATCGCCATCGGAGCGTCTGGTCTGGTGCTGGGGATCATCATAGCAGCTGCTGGACTCATT-ACTACAAGAAGAAATCAACAGGGAGGATCCTGGTACCAA

>Rhsi-DAB1*02:08

CTGATGCTGTCGACTTTCACCGGAGCA-----------------------------------------------------------------------------------------------------------------------------------------------------------------------------------------------------------------------GCTCATGGATACTACTGGTCTATGTGGTCTAAATGCGTCCACAGCTCCCGGGATTTCAGCGACATGGTGTTCATTGATAACTATATCTTCAATAAAGATGTGTTCATACAGTTCAACAGCACTGTGGGAGAGTATGTGGGGTACACTGAACATGGAGTATATAATGCACGGAATTGGAACAGCGATCCCAACATTCTGCAGCAAGAGAGAGCTGAGTTGGAGAGATACTGCAAATATAATGCTGAACTCTATCAGGCAGCTATCGCT---GATAAAACA------------------------------------------------------------------------------------------------------------------------------------------------------------------------------------------------------------------------------------------------------------------------------------------------------------------------------------------------------------------------------------------------GTGGCACCAAAGGTCAAGCTCAGTTCAGTGATGCAGGCCAGCGGCAGTCATCCTGCTGTACTGATGTGCAGCGCTTACCGCTTCTACCCACACTGGATCAAAGTGTCCTGGATGAGGGATGGTAAAGTTGTGAAGACTGACGTGACCTCAACTGAGGAGATGCCTAACGGAGACTGGTACTATCAGATTCACTCGCACCTGGAATACACTCCCAAATCTGGAGAGAAGATCTCCTGTGTGGTGGATCACGCCGGCTTAACTAAATCCATCATCGTAGACTGG-----------------------------------------------------------------------------------------------------------------------------------------------------------------------GATCCCGCTATGCCAGAGTCTGACAGGAATAAAATCGCCATCGGAGCGTCTGGTCTGGTGCTGGGGATCATCATAGCAGCTGCTGGACTCATTTACTACAAGAAGAAATCAACAGGGAGGATCCTGGTACCAA

>Rhsi-DAB1*03:01

CTGATGCTGTATGCTTTCACTGGAGAA-----------------------------------------------------------------------------------------------------------------------------------------------------------------------------------------------------------------------GCAAATGGATATTACTATTCTGGGTGGTGTAGATGCATCCACAGCTCCCGTGATCTCAGTGACATGGTGTTCATTTTTAATAAAGTCTTCAACAAAGATGTGTTCGCACAGTTCAACAGCACTGTAGGGGAGTTTGTGGGGTACACTGCACATGGAGTATATAATGCAGAATTATGGAACAAAGATCCCAACATTCTGGCACAATGGAGAGGTGAGAAGGACAGAGTCTGCAATCATAATGCTGAAATCAGACAGTCAGCTATCGCT---GATAAAAAA------------------------------------------------------------------------------------------------------------------------------------------------------------------------------------------------------------------------------------------------------------------------------------------------------------------------------------------------------------------------------------------------GTGGCACCAGAGGTCAAGCTCAGTTCAGTGACGCCGGCTGGAGGCAGACATCCAGCTGTCTTGATGTGCAGCGCTTACAACTTCTACCCACACCAGATCACAGTTTCCTGGATGAGAGATGATAAAGTTGTGAAGTCTGATGTGACTTCAACTGAGGAGATGGCTGATGGAGACTGGTACTACCAGATCCACTCTCACCTAGAATACACTCCCAAATCTGGAGAGAAGATCTCCTGTGCCGTGGATCACGCCGGCTTAACTAAACCCACCATCATAGGCTGG-----------------------------------------------------------------------------------------------------------------------------------------------------------------------GATCCATCTCTTCCTGAGCCTGACAGGAATAAAATCGCTATAGGAGCTTCTGGTCTGGTGCTGGGAATCATCCTTGCAGCTGCTGGACTCATTTACTACAAGAAGAAATCAACAGGGAGGATCCTGGTACCAAACTGA

>Rhsi-DAB1*03:02

CTGATGCTGTCTGCTTTCACTGGAGAA-----------------------------------------------------------------------------------------------------------------------------------------------------------------------------------------------------------------------GCAAACGGATACTACAATTCTAATTGGTTTAGATGCATCCACAGCTCCCGTGATCTCAGTGACATGGTGTTCATTCTTAACTATGTCTTCAATAAAGATGTGTACATACAGTTCAACAGCACTGTAGGGGAGTTTGTGGGGTACACTGCACATGGAGTATATAATGCAGAATTATTCAACAAAGATCCCAACAGAATGCAGCAAATGAGAGGTGAGAAGGACAGATACTGCCTTCATAATGCTGAAATCAGACAGTCAGCTACTGCT---GATAAAAAA------------------------------------------------------------------------------------------------------------------------------------------------------------------------------------------------------------------------------------------------------------------------------------------------------------------------------------------------------------------------------------------------GTGGCACCAGAGGTCAAGCTCAGTTCAGTGACGCCGGCTGGAGGCAGACATCCAGCTATGTTGATGTGCAGCGCTTACAACTTCTACCCACACCAGATCAAAGTTTACTGGATGAGAGATGATAAAGTTGTGAAGTCTGATGTGACTTCAACTGAGGAGATGGCTGATGGAGACTGGTACTACCAGATCCACTCTCACCTAGAATACACTCCCAAATCTGGAGAGAAGATCTCCTGTGCCGTGGATCGCGCCGGCTTAACTAAACCCACCATCATAGACTGG-----------------------------------------------------------------------------------------------------------------------------------------------------------------------GATCCATCTCTTCCTGAGCCTGACAGGAATAAAATCGCTATAGGAGCTTCTGGTCTGGTGCTGGGAATCATCCTCGCAGCTGCTGGACTCATTTACTACAAGAAGAAATCATCAGGGAGGATCCTGGTACCAAACTGA

>Rhsi-DAB1*03:03

CTGATGCTGTCTGCTTTCACTGGAGAA-----------------------------------------------------------------------------------------------------------------------------------------------------------------------------------------------------------------------GCAAATGGATACTACAGTTCTTATTGGTTTAGATGCATCCACAGCTCCCGTGATCTCAGTGACATGGTGTTCGTTCTTAACTATGTCTTCAATAAAGATGTGTACATACAGTTCAACAGCACTGTTGGGGAATTTGTGGGGTACACTGCACTGGGAGTACATAATGCAGAATTATGGAACAAAGATCCCAACCTTCTGGCGCAATGGAGAGCTCAGAAGGACGTATACTGCAAACATAATGCTGAAATCAGACAGTCAGCTATCGCT---GATAAAAAA------------------------------------------------------------------------------------------------------------------------------------------------------------------------------------------------------------------------------------------------------------------------------------------------------------------------------------------------------------------------------------------------GTGGCACCAGAGGTCAAGCTCAGTTCAGTGACTCAGGCCAGTGGCAGACATCCAGCTATGTTGATGTGCAGCGCTTACAACTTCTACCCACACCAGATCAAAGTTTACTGGATGAGAGACGGTAAAGTTGTGAAGTCTGATGTGACTTCAACTGAGGAGATGCCTAATGGTGACTGGTACTACCAGATCCACTCTCACCTAGAATACACTCCCAAATCTGGAGAGAAGATCTCCTGTGCCGTGGATCACGCCGGCTTAACTAAACCCACCATCATAGACTGG-----------------------------------------------------------------------------------------------------------------------------------------------------------------------GATCCCCCTCTCCCTGAGCCTGACAGGAATAAAATCGCCATTGGAGCTTCTGGTCTGGTGCTGGGAATCATCCTCGCAGCTGTTGGACTCATTTACTACAAGAAGAAATCGACAGGGAGGATCCTGGTACCAAACTGA

>Rhsi-DAB1*03:04

CTGATGCTGTCTGCTTTCACTGGAGAA-----------------------------------------------------------------------------------------------------------------------------------------------------------------------------------------------------------------------GCAAATGGATACTACCATTCTAATTGGTATAGATGCATCCACAGCTCCCGTGATCTCAGTGACATGGTGTTCGTTCTTAACTATGTCTTCAATAAAGATGTGGACATACAGTTCAACAGCACTGTAGGGGAGTTTGTGGGGTACACTGCACATGGAGTATATAATGCAGAATTATGGAACAAAGATCCCAACAGAATGCAGCAAATGAGAGGTGAGAAGGACAGATACTGCCTTCATAATGCTGAAATCAGACAGTCAGCTATCGCT---GATAAAAAA------------------------------------------------------------------------------------------------------------------------------------------------------------------------------------------------------------------------------------------------------------------------------------------------------------------------------------------------------------------------------------------------GTGGCACCAGAGGTCAAGCTCAGTTCAGTGACTCAGGCCAGTGGCAGACATCCAGCTATGTTGATGTGCAGCGCTTACAACTTCTACCCACACCAGATCAAAGTTTACTGGATGAGAGACGGTAAAGTTGTGAAGTCTGATGTGACTTCAACTGAGGAGATGCCTAATGGAGACTGGTACTACCAGATCCACTCTCACCTAGAATACACTCA

>Rhsi-DAB1*03:05

CTGATGCTGTCTGCTTTCACTGGAGAAGGTAAATAAAATGGAGTATAATGCAAAATGGAAAGATATATAATGACAGTAATCTTCTTCTCATCAATTATTATTGGTAGTAGAAAT------------GACAACTCACTAATACAACGATTGACTAATGTATCAAAATCCTTCTGTTTTTGTATGGTTGACAAAATCATTTGACATGACTGTAATGCTGATTTAACATA---ATTTTTTTTTCAGCAAATGGATACTACCGTTCTGATTGGTATAGATGCATCCACAGCTCCCGTGATCTCAGTGACATGGTGTTCATTTATAATAAAATCTTCAATAAAGATGTGTACATACAGTTCAACAGCACTTTTGGGGAGTTTGTGGGGTACACTGCACATGGAGTATATAATGCAGAATTATACAACAAAGATCCCAACATTCTGGCACAAATGAGAGGTGAGAAGGACAGATACTGCCATCATAATGCTGAAGTACATCAGTCAGCTATTGCT---GATAAAAAA---GGTAAGCAGCACAAGATTCTGATCTCTCCTGTCACACATGAAACTC--ATTTTAAATGCGTACACTAGGTTCTTTTACACAACACTTTGATAATTTTATAATTATTTAAAAACTTGTGTTATGATAACACCAGGACAGTCTTTAACTCCATAACTAACATTTTAAAATGTCACAATAATAAAAACATGCTTATGCTTTAATTTTTGTTTGCCCTTGGAAATATTACAAATACACCCCCAAACACATACATACAATAATATTTATTTT----ATGTGTTTGGGA---GTGTATTTCTAATATTTAACTTTATAGTACACCTTGCATGTAATAATA---TTTATTGTAGGATGTGCCCTTAAATTGTTATTGTTATTGATTCAGTGGCACCAGAGGTCAAGCTCA

>Rhsi-DAB1*03:06

CTGATGCTGTCTGCTTTCACTGGAGAA-----------------------------------------------------------------------------------------------------------------------------------------------------------------------------------------------------------------------GCAAATGGATACTACAATTCTGTGTGGTATAGATGCATCCACAGCTCCCGTGATCTCAGTGACATGGTGTTCATCAAGGACAGTATCTTCAATAAAGATGTGTACATACAGTTCAACAGCACCGTAGGGGAGTTTGTGGGGTACACTGCGCTGGGAGCACATAATGCAGAATTATGGAACAAAGATCCCAACCTTCTGGCGCAATGGAGAGCTCAGAAGGACGTATACTGCAAACATAATGCTGAAATCAGACAGTCAGCTATCGCT---GATAAAAAA------------------------------------------------------------------------------------------------------------------------------------------------------------------------------------------------------------------------------------------------------------------------------------------------------------------------------------------------------------------------------------------------GTGGCACCAGAGGTCAAGCTCAATTCGGTGACTCAGGCCAGTGGCAGACATCCAGCTATGTTGATGTGCAGCGCTTACAACTTCTACCCACACCAGATCAAAGTTTACTGGACGAGAGACGGTAAAGTTGTGAAGTCTGATGTGACTTCAACTGAGGAGATGCCTAATGGAGACTGGTACTACCAGATCCACTCTCACCTAGAATACACTCCCAAATCTGGAGAGAAGATCTCCTGTGCCGTGGATCACGCCAGCTTAACTAAACCCATCATCATAGACTGG-----------------------------------------------------------------------------------------------------------------------------------------------------------------------GATCCATCTCTTCCTGAGCCTGACAGGAATAAAATCGCCATTGGAGCTTCTGATCTGGTGCTGGGAATCATCCTTGCAGCTGCTGGACTCATTTACTACAAGAAGAAGTCATCAGGGAGGATCCTGGTACCAAACTGA

>Rhsi-DAB1*03:07

CTGATGCTGTCTGCTTTCACTGGAGAAGGTAAATAAAATGAAGCATAATGCAAAATGAAAAGATATATAATGACAGTAACCTTATTCTCATTAACTATTATTGGTAGTAGAAGTAGTAGTAGAAAAGACAACTTACTAATATAACAACTGACTAATGTATCAAAATCCTTCTGTTTTTGTATGGTTGACAAAATCATTTGACATAACTCTAATGCTGATTTAAAATCTTTTTTTTTTTTTCAGCAAATGGATACTACAATTCTAATTGGTTCAGATGCATCCACAGCTCCCGTGATCTCAGTGACATGGTGTTCATTCAGAACTATGTCTTCAATAAAGATGTGTTCCTACAGTTCAACAGCACTGTAGGGGAGTTTGTGGGATACACTGAACTTGGAGTAAAAAATGCAGAAAGATTCAACAAAGATCCCAACATTCTGCACCAATGGAGAGCTCAGAAGGACACATACTGCACAAAGAATGCTGAAATAGATTACACCAATCATCTT---ATTGGCAAAGCAGGTAAGCAGCACAAGATTCTGATCTCTCCTGTCACACATTAAACTCTTGTTTAAAATGCTTAAACTAAGTGCTTTTACGCAACAGTTTGATAATTTTATAATTCTTTAAAAACTTT---------AAAGACAACACATTCTTTAACGTCATAACCAACATTTTAAAATTTCATAATAACAAAAACATGCTTATGTTTT-----------------------------------CTCCCAAACACATACATACAATAATATTTATTTTATATATGTGTTTGGGAGGAGTGTATTT-TAATATTTAACTTTATA-----CCTTGCATGTAATAATA---TTTATTGTAGGATGTGCCCTCAAGTTGTTCTTGTTATTGATTCAGTGGCACCAGAGGTCAAGCTCA

>Rhsi-DAB1*03:08

CTGATGCTGTCTGCTTTCACTGGAGAA-----------------------------------------------------------------------------------------------------------------------------------------------------------------------------------------------------------------------GCAAATGGATATTACTATTCTGGGTGGTATAGATGCATCCACAGCTCCCGTGATCTCAGTGACATGGTGTTCATTTTTAATAAAGTCCTCAACAAAGATGTGTTCGCACAGTTCAACAGCACTGTAGGGGAGTTTGTGGGGTACACTGCACATGGAGTATATAATGCAGAATTATGGAACAAAGATCCCAACATTCTGGCACAATGGAGAGGTGAGAAGGACAGAGTCTGCAATCATAATGCTGAAATCAGACAGTCAGCTATCGCT---GATAAAAAA------------------------------------------------------------------------------------------------------------------------------------------------------------------------------------------------------------------------------------------------------------------------------------------------------------------------------------------------------------------------------------------------GTGGCACCAGAGGTCAAGCTCAA

>Rhsi-DAB1*03:09

CTGATGCTGTCTGCTTTCACTGGAGAA-----------------------------------------------------------------------------------------------------------------------------------------------------------------------------------------------------------------------GCAAATGGATACTACAGTTCTTATTGGTTTAGATGCATCCACAGCTCCCGTGATCTCGGTGACATGGTGTTCGTTCTTAACTATGTCTTCAATAAAGATGTGTACATACAGTTCAACAGCACTGTTGGGGAATTTGTGGGGTACACTGCACTGGGAGTACATAATGCAGAATTATGGAACAAAGATCCCAACCTTCTGGCGCAATGGAGAGCTCAGAGGGACGTATACTGCAAACATAATGCTGAAATCAGACAGTCAGCTATCGCT---GATAAAAAA------------------------------------------------------------------------------------------------------------------------------------------------------------------------------------------------------------------------------------------------------------------------------------------------------------------------------------------------------------------------------------------------GTGGCACCAGAGGTCAAGCTCAGTTCAGTGACTCAGGCCAGTGGCAGACATCCAGCTATGTTGATGTGCAGCGCTTACAACTTCTACCCACACCAGATCAAAGTTTACTGGATGAGAGACGGTAAAGTTGTGAAGTCTGATGTGACTTCAACTGAGGAGATGCCTAATGGAGACTGGTACTACCAGATCCACTCTCACCTAGAATACACTCA

>Rhsi-DAB1*03:10

-----------------------------------------------------------------------------------------------------------------------------AGATTACTCACTAATACAATAAATTGACTAATGTATCAAATCCTTGTGTTTTTGTATGGTTGACAAAATCATTTGACATGACTGTAATGCTGATTTAACAATA-----TTTTTTTCAGCAAATGGATACTACAATTCTGTGTGGTATAGATGCATCCACAGCTCCCGTGATCTCAGTGACATGGTGTTCATCAAGGACAGTATCTTCAATAAAGATGTGTACATACAGTTCAACAGCACTGTAGGGGAGTTTGTGGGGTACACTGCACTGGGAGTACATAATGCAGAATTATGGAACAAAGATCCCAACCTTCTGGCGCAATGGAGAGCTCAGAAGGACGTATACTGCAAACATAATGCTGAAATCAGACAGTCAGCTATCGCT---GATAAAAAA---GGTAAGCAGCACAAGATTCTGATCTCTTCTGTCACACATGAAACTC--GTTTTAA-TGCTTACA---GGCACTTTAACACAACAGTTTGATAATTTTATAATTCTTTAAAAACTTGTGTTATGATAACACCAAGACAGTCTTTAACTCCATAACTAACATTTTAAAATGTCACAATAGCAAAAACATGCTTATGTTTTAATTTTTGTTTGCCCTTGGAAATATTAGAAATACAC-CCCAAACACATACATACAATAATACTTATTTTATATATGTGTTCGGGA---GTGTATTTCTAATATTTAACTTTATAGTACACCTTGCATGCAATAATAATATTTATTATTGGATGTACCCTCAAGATGTTATTGTTATTGATTCAGTGGCACCAGAGGTCAAGCTCA

>Rhsi-DAB1*03:11

-----------------------------------------------------------------------------------------------------------------------------AGACAACTTACTAATATAACAACTGACTAATGTATCAAAATCCTTCTGTTTTTGTATGGTTGACAAAATCATTTGACATAACTCTAATGCTGATTTAAAATC-TTTTTTTTTTTTCAGCAAATGGATACTACAATTCTAATTGGTTCAGATGCATCCACAGCTCCCGTGATCTCAGTGACATGGTGTTCATTCAGAACTATGTCTTCAATAAAGATGTGTACATACAGTTCAACAGCACTTTTGGGGAGTTTGTGGGGTACACTGCACATGGAGTATATAATGCAGAATTATGGAACAAAGATCCCAACCTTCTGGCGCAATGGAGAGGTGAGAAGGACAGATTCTGCAATCATAATGCTGAAGTATATCAGTCAGCTATCGCT---GATAAAAAA---GGTAAGCAGCACAAGATTCTGATCTCTCCTGTCACACATGAAACTC--ATTTTAAATGCGTACACTAGGTTCTTTTACACAACACTTTGATAATTTTATAATTATTTAAAAACTTGTGTTATGATAACACCAGGACAGTCTTTAACTCCATAACTAACATCTTAAAATGTCACAATAACAAAAACATGCTTATGTTTTAACTTTTGTTTGCCCTTGGAAATATTAGAAATACACCCCCAAACACATACATACAATAATATTTATTTT----ATGTGTTTGGGA---GTGTATTTCTAATATTTAACTTTATAGTACTCCTTGCATGTAATAATA---TTTATTGTAGGATGTGCCCTCAAGTTGTTCTTGTTATTGATTCAGTGGCACCAGAGGTCAAGCTCA

>Rhsi-DAB1*03:12

CTGATGCTGTCTGCTTTCACTGGAGAA-----------------------------------------------------------------------------------------------------------------------------------------------------------------------------------------------------------------------GCAAATGGATACTACCATTCTAATTGGGCTAGATGCATCCACAGCTCCCGTGATCTCAGTGACATGGTGTTCGTTCTTAACTATGTCTTCAATAAAGATCTGGTCGTACAGTTCAACAGCACTGTTGGGGAATTTGTGGGTTACACTGCACTTGGAGTGCATAATGCAGAATTATGGAACAAAGATCCCAACATTCTGGCACAATGGAGAGGTGAGAAGGACAGATACTGCCTTCATAATGCTGAAATCAGACAGTCAGCTATTGCT---GATAAAAAA------------------------------------------------------------------------------------------------------------------------------------------------------------------------------------------------------------------------------------------------------------------------------------------------------------------------------------------------------------------------------------------------GTGGCACCAGAGGTCAAGCTCAGTTCAGTGACGCCGGCTGGAGGCAGACATCCAGCTGTCTTGATGTGCAGCGCTTACAACTTCTACCCACACCAGATCACAGTTTCCTGGATGAGAGATGGTAAAGTTGTGAAGTCTGATGTGACTTCAACTGAGGAGATGGCTGATGGAGACTGGCACTACCAGATCCACTCTCACCTAGAATACACTCCCAAATCTGGAGAGAAGATCTCCTGTGCCGTGGATCACGCCGGCTTAACTAAACCCACCATCATAGACTGG-----------------------------------------------------------------------------------------------------------------------------------------------------------------------GATCCATCTCTTCCTGAGCCTGACAGGAATAAAATCGCTATAGGAGCTTCTGGTCTGGTGCTGGGAATCATCCTTGCAGCTGCTGGACTCATTTACTACAAAAAGAAATCAACAGGGAGGATCCTGGTACCAAACTGA

>Rhsi-DAB1*03:13

CTGATGCTGTCTGCTTTCACTGGAGAA-----------------------------------------------------------------------------------------------------------------------------------------------------------------------------------------------------------------------GCAAATGGATATTACTATTCTGGGTGGTATAGATGCATCCACAGCTCCCGTGATCTCAGTGACATGGTGTTCATTTTTAATAAAGTCTTCAACAAAGATGTGTTCGCACAGTTCAACGGCACTGTAGGGGAGTTTGTGGGGTACACTGCACATGGAGTATATAATGCAGAATTATGGAACAAAGATCCCAACATTCTGGCACAATGGAGAGGTGAGAAGGACAGAGTCTGCAATCATAATGCTGAAATCAGACAGTCAGCTATCGCT---GATAAAAAA------------------------------------------------------------------------------------------------------------------------------------------------------------------------------------------------------------------------------------------------------------------------------------------------------------------------------------------------------------------------------------------------GTGGCACCAGAGGTCAAGCTCAA

>Rhsi-DAB1*03:14

CTGATGCTGTCTGCTTTCACTGGAGCA-----------------------------------------------------------------------------------------------------------------------------------------------------------------------------------------------------------------------GCTCATGGATACTACTGGTCTATGTGGTCTAAATGCATCCACAGCTCCCGGGATTTCAGCGACATGGTGTTCATTGATAACTATATCTTCAATAAAGATGTGTTCATACAGTTCAACAGCACTGTGGGAGAGTATGTGGGGTACACTGAACTTGGAGTATATAATGCACGAAGAATGAACAACGATCCCAACCGTCTGCAGCAAGAGAGAGCTGAGGTGGAGAGATACTGCAAACATAATGCTGAAATCTATCAGGCAGCTATCGCT---GATAAAACA------------------------------------------------------------------------------------------------------------------------------------------------------------------------------------------------------------------------------------------------------------------------------------------------------------------------------------------------------------------------------------------------GTGGCACCAGAGGTCAAGCTCAA

>Rhsi-DAB1*03:15

CTGATGCTGTCTGCTTTCACTGGAGAA-----------------------------------------------------------------------------------------------------------------------------------------------------------------------------------------------------------------------GCAAATGGATACTACAATTCCGTGTGGTATAGATGCATCCACAGCTCCCGTGATCTCAGTGACATGGTGTTCATCAAGGACAGTATCTTCAGTAAAGATGTGTACATACAGTTCAACAGCACCGTAGGGGAGTTTGTGGGGTACACTGCACTGGGAGTACATAATGCAGAATTATGGAACAAAGATCCCAACCTTCTGGCGCAATGGAGAGCTCAGAAGGACGTATACTGCAAACATAATGCTGAAATCAGACAGTCAGCTATCGCT---GATAAAAAA------------------------------------------------------------------------------------------------------------------------------------------------------------------------------------------------------------------------------------------------------------------------------------------------------------------------------------------------------------------------------------------------GTGGCACCAGAGGTCAAGCTCAA

>Rhsi-DAB1*03:16

CTGATGCTGTCTGCTTTCACTGGAGAA-----------------------------------------------------------------------------------------------------------------------------------------------------------------------------------------------------------------------GCAAATGGATACTACAATTCTGTGTGGTATAGATGCATCCACAGCTCCCGTGATCTCAGTGACATGGTGTTCATCAAGGACAGTATCTTCAATAAAGATGCGTACATACAGTTCAACAGCACTGTAGGGGAGTTTGTGGGGTACACTGCATTGGGAGTACATAATGCAGAATTATGGAACAAAGATCCCAACCTTCTGGCGCAATGGAGAGCTCAGAAGGACGTATACTGCAAACATAATGCTGAAATCAGACAGTCAGCTATCGCC---GATAAAAAA------------------------------------------------------------------------------------------------------------------------------------------------------------------------------------------------------------------------------------------------------------------------------------------------------------------------------------------------------------------------------------------------GTGGCACCAGAGGTCAAGCTCA

>Rhsi-DAB1*03:17

CTGATGCTGTCTGCTTTCACTGGAGAA-----------------------------------------------------------------------------------------------------------------------------------------------------------------------------------------------------------------------GCAAATGGATACTACAGTTCTTATTGGTTTAGATGCATCCACAGCTCCCGTGATCTCAGTGACATGGTGTTCGTCCTTAGCTATGTCTTCAATAAAGATGTGTACATACAGTTCAACAGCACTGTTGGGGAATTTGTGGGTTACACTGCACTTGGAGTGCATAATGCAGAATTATGGAACAAAGATCCCAACATTCTGGCACAATGGAGAGGTGAGAAGGACAGATACTACCTTCATAATGCTGAAATCAGACAGTCAGCTATTGCT---GATAAAAAA------------------------------------------------------------------------------------------------------------------------------------------------------------------------------------------------------------------------------------------------------------------------------------------------------------------------------------------------------------------------------------------------GTGGCACCAGAGGTCAAGCTCAGTTCAGTGACGCCGGCTGGAGGCAGACATCCAGCTGTCTTGATGTGCAGCGCTTACAACTTCTACCCACACCAGATCACAGTTTCCTGGATGAGAGATGATAAAGTTGTGAAGTCTGATGTGACTTCAACTGAGGAGATGGCTGATGGAGACTGGTACTACCAGATCCACTCTCACCTAGAATACACTCCCAAATCTGGAGAGAAGATCTCCTGTGCCGTGGATCACGCCGGCTTAACTAAACCCACCATCATAGACTGG-----------------------------------------------------------------------------------------------------------------------------------------------------------------------GATCCCTCTCTCCCTGAGCCTGACAGGAATAAAATCGCCATTGGAGCTTCTGGTCTGGTGCTGGGAATCATCCTCGCAGCTGCTGGACTCATTTACTACAAGAAGAAATCAACAGGGAGGATCCTGGTACCAAACTGA

>Rhsi-DAB1*03:18

CTGATGCTGTCTGCTTTCACTGGAGCA-----------------------------------------------------------------------------------------------------------------------------------------------------------------------------------------------------------------------GCAAATGGATACTACAGTTCTTATTGGTTTAGATGCATCCACAGCTCCCGTGATCTCAGTGACATGGTGTTCGTTCTTAGCTATGTCTTCAATAAAGATGTGTACATACAGTTCAACAGCACTGTTGGGGAATTTGTGGGTTACACTGCACTTGGAGTGCATAATGCAGAATTATGGAACAAAGATCCCAACATTCTGGCACAATGGAGAGGTGAGAAGGACAGATACTGCCTTCATAATGCTGAAATCAGACAGTCAGCTATTGCT---GATAAAAAA------------------------------------------------------------------------------------------------------------------------------------------------------------------------------------------------------------------------------------------------------------------------------------------------------------------------------------------------------------------------------------------------GTGGCACCAGAGGTCAAGCTCAGTTCAGTGACGCCGGCTGGAGGCAGACATCCAGCTGTCTTGATGTGCAGCGCTTACAACTTCTACCCACACCAGATCACAGTTTCCTGGATGAGAGATGATAAAGTTGTGAAGTCTGATGTGACTTCAACTGAGGAGATGGCTGATGGAGACTGGTACTACCAGATCCACTCTCACCTAGAATACACTCCCAAATCTGGAGAGAAGATCTCCTGTGCCGTGGATCACGCCGGCTTAACTAAACCCACCATCATAGACTGG-----------------------------------------------------------------------------------------------------------------------------------------------------------------------GATCCCTCTCTCCCTGAGCCTGACAGGAATAAAATCGCCATTGGAGCTTCTGGTCTGGTGCTGGGAATCATCCTCGCAGCTGCTGGACTCATTTACTACAAGAAGAAATCAACAGGGAGGATCCTGGTACCAAACTGA

>Rhsi-DAB1*04:01

CTGATGCTGTCTGCTTTCACCGGAGCA-----------------------------------------------------------------------------------------------------------------------------------------------------------------------------------------------------------------------GCTCATGGATACTACAATTCTGTGTGGTCTAAATGCATCCACAGCTCCCGGGATTTCAGCGACATGGTGTTCATTGATAACTATGTCTTCAATAAAGATGTGTACATACAGTTCAACAGCACTGTGGGAGAGTATGTGGGGTACACTGAACTTGGAGTAAGTAATGCACGGAATTGGAACAACGATCCCAACATTCTGCAGCAAGAGAGAGCTCAGGTGGAGACATACTGCAAACCTAATGCTGAAATAGATTACAGCTCTAATCTTATTGGTAAAGCA------------------------------------------------------------------------------------------------------------------------------------------------------------------------------------------------------------------------------------------------------------------------------------------------------------------------------------------------------------------------------------------------GTGGCACCACAGGTCAAACTCAGTTCAGTGACGCAGGCTGGCGGCAGACATCCTGCTGTACTGATGTGCAGCGCTTATCACTTCTACCCACGCTGGATCAAAGTGTCCTGGATGAGAGATGATAAAGTTGTGAAGACTGACGTGACCTCAACTGAGGAGATGCCTAACGGAGACTGGTACTACCAGATTCACTCGCACCTGGAATACACTCCCAAATCTGGAGAGAAGATCTCCTGTGTGGTGGATCACGCCAGCTTAACTAAATCCATCATCGTAGACTGG

>Rhsi-DAB1*04:02

CTGATGCTGTCTGCTTTTACTGGAGCA-----------------------------------------------------------------------------------------------------------------------------------------------------------------------------------------------------------------------GCTCATGGATACTACAATTCTGTGTGGTCTAAATGCATCCACAGCTCCCGGGATTTCAGCGACATGGTGTTCATTGATAACTATGTCTTCAATAAAGATGTGAACATACAGTTCAACAGCACTGTGGGAGAGTATGTGGGGTACACTGAACTTGGAGTAAGTAATGCACGGAATTGGAACAACGATCCCAACATTCTGCAGCAAGAGAGAGCTCAGGTGGAGACATACTGCAAACCTAATGCTGAAATAGATTACAGCTCTAATCTTATTGGTAAAGCA------------------------------------------------------------------------------------------------------------------------------------------------------------------------------------------------------------------------------------------------------------------------------------------------------------------------------------------------------------------------------------------------GTGGCACCACAGGTCAAACTCAGTTCAGTGACGCAGGCTGGCGGCAGACATCCTGCTGTACTGATGTGCAGCGCTTATCACTTCTACCCACGCTGGATCAAAGTGTCCTGGATGAGAGATGATAAAGTTGTGAAGACTGACGTGACCTCAACTGAGGAGATGCCTAACGGAGACTGGTACTACCAGATTCACTCGCACCTGGAATACACTCCCAAATCTGGAGAGAAGATCTCCTGTGTGGTGGATCACGCCAGCTTAACTAAATCCATCATCGTAGACTGG-----------------------------------------------------------------------------------------------------------------------------------------------------------------------GATCCATCTCTTCCTGAGTCTGAGAGGAATAAAATCGCCATCGGGGCGTCTGGTCTGGTGCTGGGGATCATCATAGCAGCTGCTGGACTCATTTACTACAAGAAGAAATCAACAGGGAGGATCCTGGTACCAAATC

>Rhsi-DAB1*05:01

CTGATGCTGTCTACTTTCACTGGAGCA-----------------------------------------------------------------------------------------------------------------------------------------------------------------------------------------------------------------------GCTAATGGATACTACAATTCTCGGTGGAATAAATGCATCCACAGCTCCCGGGATTTCAGCGACATGGTGTTCATTGATAACTATATCTTCAATAAAGATGTGGTTGTACAGTTCAATAGCACTGTGGGAGAGTTTGTGGGGTACACTGAACTTGGAGTAAGTAGTGCACGGAATTGGAACAGCGATCCCAACCGTCTGCAGCAAGAGAGAGCTGAGTTGGAGGGATACTGCAAACATAATGCTGAAATCAGACAGGCATCTATCACT---GATAAAACA------------------------------------------------------------------------------------------------------------------------------------------------------------------------------------------------------------------------------------------------------------------------------------------------------------------------------------------------------------------------------------------------GTGGCACCAAAGGTCAAGCTCAGTTCAGTGACGCAGGCCGGCGGCAGACATCCTGCTGTACTGATGTGCAGCGCTTACCGCTTCTACCCGCACTGGATCAAAGTGTCCTGGATGAGAGACGGTACAGTTGTGAAGACTGATGTGACCTCAACTGAGGAGATGCCTAACGGAGACTGGTACTACCAGATTCACTCGCACCTGGAGTACACTCCCAAATCTGGAGAGAAGATCTCCTGTGTGGTGGATCACGCCGGCTTAACTAAATCCATCATCGTAGACTGG-----------------------------------------------------------------------------------------------------------------------------------------------------------------------GATCCCGCTATGCCTGAGTCTGACAGGAATAAAATCGCCATCGGGGCGTCTGGTCTGGTGCTGGGGATCATCATAGCAGCTGCTGGACTCATTTATTACAAGAAGAAATCAACAGGGAGGATCCTGGTACCAAATC

>Rhsi-DAB1*05:02

CTGATGCTGTCGGCTTTTACTGGAGCA-----------------------------------------------------------------------------------------------------------------------------------------------------------------------------------------------------------------------GCTAATGGATACTACAATTCTCGGTGGAATAAATGCACCCACAGCTCCCGGGATTTCAGCGACATGGTGTTCATTGATAACTATATCTTCAATAAAGATGTGGTTGTACAGTTCAATAGCACTGTGGGAGAGTTTGCGGGGTACACTGAACTTGGAGTAAGTAGTGCACGGAATTGGAACAGCGATCCCAACCGTCTGCAGCAAGAGAGAGCTGAGTTGGAGAGATACTGCAAACATAATGCTGAAATCAGACAGGCATCTATCGCT---GATAAAACA------------------------------------------------------------------------------------------------------------------------------------------------------------------------------------------------------------------------------------------------------------------------------------------------------------------------------------------------------------------------------------------------GTGGCACCAAAGGTCAAGCTCAGTTCAGTGACGCAGGCCGGCGGCAGACATCCTGCTGTACTGATGTGCAGCGCTTACCGCTTCTACCCGCACTGGATCAAAGTGTCCTGGATGAGAGACGGTACAGTTGTGAAGACTGATGTGACCTCAACTGAGGAGATGCCTAACGGAGACTGGTACTACCAGATTCACCCGCACCTGGAGTACACTCCCAAATCTGGAGAGAAGATCTCCTGTGTGGTGGATCACGCCGGCTTAACTAAATCCATCATCGTAGACTGG-----------------------------------------------------------------------------------------------------------------------------------------------------------------------GATCCCGCTATGCCTGAGTCTGACAGGAATAAAATCGCCATCGGGGCGTCTGGTCTGGTGCTGGGGATCATCATAGCAGCTGCTGGACTAATTTATTACAAGAAGAAATCAACAGGGAGGATCCTGGTACCAAA

>Rhsi-DAB1*05:03

CTGATGCTGTCTGCTTTCACTGGAGCA-----------------------------------------------------------------------------------------------------------------------------------------------------------------------------------------------------------------------GCTAATGGATACTACAATTCTCGGTGGAATAAATGCATCCACAGCTCCCGGGATTTCAGCGACATGGTGTTCATTGATAACTATATCTTCAATAAAGATGTGGTTGTACAGTGCAATAGCACTGTGGGAGAGTTTGTGGGGTACACTGAACTTGGAGTAAGTAGTGCACGGAATTGGAACAGCGATCCCAACCGTCTGCAGCAAGAGAGAGCTGAGTTGGAGAGATACTGCAAACATAATGCTGAAATCAGACAGGCATCTATCGCT---GATAAAACA------------------------------------------------------------------------------------------------------------------------------------------------------------------------------------------------------------------------------------------------------------------------------------------------------------------------------------------------------------------------------------------------GTGGCACCAGAGGTCAAGCTCA

>Rhsi-DAB1*05:04

CTGATGCTGTCTGCTTTCACCGGAGCA-----------------------------------------------------------------------------------------------------------------------------------------------------------------------------------------------------------------------GCTAATGGATACTACAATTCTCGGTGGAATAAATGCATCCACAGCTCCCGGGATTTCAGCGACATGGTGTTCATTGATAACTATATCTTCAATAAAGATGTGGTTGTACAGTTAAATAGCACTGTGGGAGAGTTTGCGGGGTACACTGAACTTGGAGTAAGTAGTGCACGGAATTGGAACAGCGATCCCAACCGTCTGCAGCAAGAGAGAGCTGAGCTGGAGAGATACTGCAAACATAATGCTGAAATCAGACAGGCATCTATCGCT---GATAAAGCA------------------------------------------------------------------------------------------------------------------------------------------------------------------------------------------------------------------------------------------------------------------------------------------------------------------------------------------------------------------------------------------------GTGGCACCAAAGGTCAAGCTCAGTTCAGTGACGCAGGCCGGCGGCAGACATCCTGCTGTACTGATGTGCAGCGCTTACCGCTTCTACCCGCACTGGATCAAAGTGTCCTGGATGAGAGACGGTACAGTTGTGAAGACTGATGTGACCTCAACTGAGGAGATGCCTAACGGAGACTGGTACTACCAGATTCACCCGCACCTGGAGTACACTCCCAAATCTGGAGAGAAGATCTCCTGTGTGGTGGATCACGCCGGCTTAACTAAATCCATCATCGTAGACTGG-----------------------------------------------------------------------------------------------------------------------------------------------------------------------AATCCCGCTATGCCTGAGTCTGACAGGAATAAAATCGCCATCAGGGCGTCTGGTCTGGTGCTGGGGATCATAATAGCAGCTGCTGGACTCATTTATTACAAGAAGAAATCAACAGGGAGGATCCTGGTACCAAA

>Rhsi-DAB1*05:05

CTGATGCTGTCTGCTTTCACTGGAGCA-----------------------------------------------------------------------------------------------------------------------------------------------------------------------------------------------------------------------GCTAATGGATACTACAATTCTCGGTGGAATAAATGCATCCACAGCTCCCGGGATTTCAGCGACATGGTGTTCATTGATAACTATATCTTCAATAGAGATGTGGTTGTACAGTTCAATAGCACTGTGGGAGAGTTTGTGGGGTACACTGAACTTGGAGTAAGTAGTGCACGGAATTGGAACAGCGATCCCAACCGTCTGCAGCAAGAGAGAGCTGAGTTGGAGAGATACTGCAAACATAATGCTGAAATCAGACAGGCATCTATCGCT---GATAAAACA------------------------------------------------------------------------------------------------------------------------------------------------------------------------------------------------------------------------------------------------------------------------------------------------------------------------------------------------------------------------------------------------GTGGCACCAGAGGTCAAGCTCAA

>Rhsi-DAB1*05:06

CTGATGCTGTCTGCTTTCACTGGAGCA-----------------------------------------------------------------------------------------------------------------------------------------------------------------------------------------------------------------------GCTAATGGATACTACAATTCTCGGTGGAATAAATGCATCCACAGCTCCCGGGATTTCAGCGACATGGTGTTCATTGATAACTATATCTTCAATAAAGATGTGGTTGTACGGTTCAATAGCACTGTGGGAGAGTTTGTGGGGTACACTGAACTTGGAGTAAGTAGTGCACGGAATTGGAACAGCGATCCCAACCGTCTGCAGCAAGAGAGAGCTGAGTTGGAGAGATACTGCAAACATAATGCTGAAATCAGACAGGCATCTATCGCT---GATAAAACA------------------------------------------------------------------------------------------------------------------------------------------------------------------------------------------------------------------------------------------------------------------------------------------------------------------------------------------------------------------------------------------------GTGGCACCAGAGGTCAAGCTCAA

>Rhsi-DAB1*05:07

CTGATGCTGTCGACTTTCACTGGAGCA-----------------------------------------------------------------------------------------------------------------------------------------------------------------------------------------------------------------------GCTAATGGATACTACAATTCTCGGTGGAATAAATGCATCCACAGCTCCCGGGATTTCAGCGACGTGGTGTTCATTGATAACTATATCTTCAATAAAGATGTGGTTGTACAGTTCAATAGCACTGTGGGAGAGTTTGTGGGGTACACTGAACTTGGAGTAAGTAGTGCACGGAATTGGAACAGCGATCCCAACCGTCTGCAGCAAGAGAGAGCTGAGTTGGAGAGATACTGCAAACATAATGCTGAAATCAGACAGGCATCTATCGCT---GATAAAACA------------------------------------------------------------------------------------------------------------------------------------------------------------------------------------------------------------------------------------------------------------------------------------------------------------------------------------------------------------------------------------------------GTGGCACCAAAGGTCAAGCTCAGTTCAGTGACGCAGGCCGGCGGCAGACATCCTGCTGTACTGATGTGCAGCGCTTACCGCTTCTACCCGCACTGGATCAAAGTGTCCTGGATGAGAGACGGTACAGTTGTGAAGACTGATGTGACCTCAACTGAGGAGATGCCTAACGGAGACTGGTACTACCAGATTCACTCGCACCTGGAGTACACTCCCAAATCTGGAGAGAAGATCTCCTGTGTGGTGGATCACGCCGGCTTAACTAAATCCATCATCGTAGACTGG-----------------------------------------------------------------------------------------------------------------------------------------------------------------------GATCCCGCTATGCCTGAGTCTGACAGGAATAAAATCGCCATCGGGGCGTCTGGTCTGGTGCTGGGGATCATCATAGCAGCTGCTGGACTCATTTATTACAAGAAGAAATCAACAGGGAGGATCCTGGTACCAAATC

>Rhsi-DAB1*05:08

CTGATGCTGTCTACTTTTACTGGAGCA-----------------------------------------------------------------------------------------------------------------------------------------------------------------------------------------------------------------------GCTAATGGATACTACAATTATCGGTGGAATAAATGCATCCACAGCTCCCGGGATTTCAACGACATGGTGTTCATTGATAACTATATCTTCAATAAAGATGTGGTTGTACAGTTCAATAGCACTGTGGGAGAGTTTGCGGGGTACACTGAACTTGGAGTAAGTAGTGCACGGAATTGGAACAGCGATCCCAACAGTCTGCAGCAAGAGAGAGCTGAGTTGGAGAGATACTGCAAACATAATGCTGAAATCAGACAGGCATCTATCGCT---GATAAAACA------------------------------------------------------------------------------------------------------------------------------------------------------------------------------------------------------------------------------------------------------------------------------------------------------------------------------------------------------------------------------------------------GTGGCACCAAAGGTCAAGCTCAGTTCAGTGACGCAGGCCGGCGGCAGACATCCTGCTGTACTGATGTGCAGCGCTTACCGCTTCTACCCGCACTGGATCAAAGTGTCCTGGATGAGAGACGGTACAGTTGTGAAGACTGATGTGACCTCAACTGAGGAGATGCCTAACGGAGACTGGTACTACCAGATTCACCCGCACCTGGAGTACACTCCCAAATCTGGAGAGAAGATCTCCTGTGTGGTGGATCACGCCGGCTTAACTAAATCCATCATCGTAGACTGA-----------------------------------------------------------------------------------------------------------------------------------------------------------------------GATCCCGCTATGCCTGAGTCTGACAGGAATAAAATCGCCATCGGGGCGTCTGGTCTGGTGCTGGGGATCATCATAGCAGCTGCTGGACTCATTTATTACAAGAAGAAATCAACAGGGAGGATCCTGGTACCAAA

>Rhsi-DAB1*05:09

CTGATGCTGTCTACATTCACTGGAGCA-----------------------------------------------------------------------------------------------------------------------------------------------------------------------------------------------------------------------GCTAATGGATACTACAATTCTCGGTGGAATAAATGCATCCACAGCTCCCGGGATTTCAGCGACATGGTGTTCATTTATAACTATATCTTCAATAAAGATGTGGTTGTACAGTTCAATAGCACTGTGGGAGAGTTTGCGGGGTACACTGAACTTGGAGTAAGTAGTGCACGGAATTGGAACAGCGATCCCAACCGTCTGCAGCAAGAGAGAGCTGAGTTGGAGAGATACTGCAAACATAATGCTGAAATCAGACAGGCATCTATCGCT---GATAAAACA------------------------------------------------------------------------------------------------------------------------------------------------------------------------------------------------------------------------------------------------------------------------------------------------------------------------------------------------------------------------------------------------GTGGCACCAAAGGTCAAGCTCAGTTCAGTGACGCAGGCCGGCGGCAGACATCCTGCTGTACTGATGTGCAGCGCTTACCGCTTCTACCCGCACTGGATCAAAGTGTCCTGGATGAGAGACGGTACAGTTGTGAAGACTGATGTGACCTCAACTGAGGAGATGCCTAACGGAGACTGGTACTACCAGATTCACCCGCACCTGGAGTACACTCCCAAATCTGGAGAGAAGATCTCCTGTGTGGTGGATCACGCCGGCTTAACTAAATCCATCATCGTAGACTGG-----------------------------------------------------------------------------------------------------------------------------------------------------------------------GATCCCGCTATGCCTGAGTCTGACAGGAATAAAATCGCCATCGGGGCGTCTGGTCTGGTGCTGGGGATCATCATAGCAGCTGCTGGACTCATTTATTACAAGAAGAAATCAACAGGGAGGATCCTGGTACCA

>Rhsi-DAB1*05:10

CTGATGCTGTCTGCTTTTACCGGAGCA-----------------------------------------------------------------------------------------------------------------------------------------------------------------------------------------------------------------------GCTAATGGATACTACAATTCTCGGTGGAATAAATGCATCCACAGCTCCCGGGATTTCAGCGACATGGTGTTCATTGATAACTGTATCTTCAATAAAGATGTGGTTGTACAGTTCAATAGCACTGTGGGAGAGTTTGCGGGGTACACTGAACTTGGAGTAAGTAGTGCACGGAATTGGAACAGCGATCCCAACCGTCTGCAGCAAGAGAGAGCTGAGTTGGAGAGATACCGCAAACATAATGCTGAAATCAGACAGGCATCTATCGCT---GATAAAACA------------------------------------------------------------------------------------------------------------------------------------------------------------------------------------------------------------------------------------------------------------------------------------------------------------------------------------------------------------------------------------------------GTGGCACCAAAGGTCAAGCTCAGTTCAGTGACGCAGGCCGGCGGCAGACATCCTGCTGTACTGATGTGCAGCGCTTACCGCTTCTACCCGCACTGGATCAAAGTGTACTGGATGAGAGACGGTACAGTTGTGAAGACTGATGTGACCTCAACTGAGGAGATGCCTAACGGAGACTGGTACTACCAGATTCACCCGCACCTGGAGTACACTCCCAAATCTGGAGAGAAGATCTCCTGTGTGGTGGATCACGCCGGCTTAACTAAATCCATCATCGTAGACTGG-----------------------------------------------------------------------------------------------------------------------------------------------------------------------GATCCCGCTATGCCTGAGTCTGACAGGAATAAAATCGCCATCGGGGCGTCTGGTCTGGTGCTGGGGATCATCATAGCAGCTGCTGGACTCATTTACTACAAGAAGAAATCAACAGGGAGGATCCTGGTACCAAA

>Rhsi-DAB1*05:11

CTGATGCTGTCTGCTTTCACTGGAGCA-----------------------------------------------------------------------------------------------------------------------------------------------------------------------------------------------------------------------GCTAATGGATACTGCAATTCTCGGTGGAATAAATGCATCCACAGCTCCCGGGATTTCAGCGACATGGTGTTCATTGATAACTATATCTTCAATAAAGATGTGGTCGTACAGTTCAATAGCACTGTGGGAGAGTTTGTGGGGTACACTGAACTTGGAGTAAGTAGTGCACGGAATTGGAACAGCGATCCCAACCGTCTGCAGCAAGAGAGAGCTGAGTTGGAGAGATACTGCAAACATAATGCTGAAATCAGACAGGCATCTATCGCT---GATAAAACA------------------------------------------------------------------------------------------------------------------------------------------------------------------------------------------------------------------------------------------------------------------------------------------------------------------------------------------------------------------------------------------------GTGGCACCAGAGGTCAAGCTCAA

>Rhsi-DAB1*05:12

CTGATGCTGTCGACATTTACTGGAGCA-----------------------------------------------------------------------------------------------------------------------------------------------------------------------------------------------------------------------GCTAATGGATACTACAATTCTCGGTGGAATAAATGCATCCACAGCTCCCGGGATTTCAGCGACATGGTGTTCATTGATAACTATATCTTCGATAAAGATGTGGTTGTACAGTTCAATAGCACTGTGGGAGAGTTTGTGGGGTACACTGAACTTGGAGTAAGTAGTGCACGGAATTGGAACAGCGGTCCCAACCGTCTGCAGCAAGAGAGAGCTGAGTTGGAGAGATACTGCAAACATAATGCTGAAATCAGACAGGCATCTATCGCT---GATAAAACA------------------------------------------------------------------------------------------------------------------------------------------------------------------------------------------------------------------------------------------------------------------------------------------------------------------------------------------------------------------------------------------------GTGGCACCAAAGGTCAAGCTCAGTTCAGTGACGCAGGCCGGCGGCAGACATCCTGCTGTACTGATGTGCAGCGCTTACCGCTTCTACCCGCACTGGATCAAAGTGTCCTGGATGAGAGACGGTACAGTTGTGAAGACTGATGTGACCTCAACTGAGGAGATGCCTAACGGAGACTGGTACTACCAGATTCACTCGCACCTGGAGTACACTCCCAAATCTGGAGAGAAGATCTCCTGTGTGGTGGATCACGCCGGCTTAACTAAATCCATCATCGTAGACTGG-----------------------------------------------------------------------------------------------------------------------------------------------------------------------GATCCCGCTATGCCTGAGTCTGACAGGAATAAAATCGCCATCGGGGCGTCTGGTCTGGTGCTGGGGATCATCATAGCAGCTGCTGGACTCATTTATTACAAGAAGAAATCAACAGGGAGGATCCTGGTACCAAATC

>Rhsi-DAB1*05:13

CTGATGCTGTCTGCATTTACTGGAGCA-----------------------------------------------------------------------------------------------------------------------------------------------------------------------------------------------------------------------GCTAATGGATACTACAATTCTCGGTGGAATAAATGCATCCACAGCTCCCGGGATTTCAGCGACATGGTGTTCATTGATAACTATATCTTCAATAAAGATGTGGTTGTACAGCTCAATAGCACTGTGGGAGAGTTTGTGGGGTACACTGAACTTGGAGTAAGTAGTGCACGGAATTAGAACAGCGATCCCAACCGTCTGCAGCAAGAGAGAGCTGAGTTGGAGAGATACTGCAAACATAATGCTGAAATCAGACAGGCATCTATCGCT---GATAAAACA------------------------------------------------------------------------------------------------------------------------------------------------------------------------------------------------------------------------------------------------------------------------------------------------------------------------------------------------------------------------------------------------GTGGCACCAAAGGTCAAGCTCAGTTCAGTGACGCAGGCCGGCGGCAGACATCCTGCTGTACTGATGTGCAGCGCTTACCGCTTCTACCCGCACTGGATCAAAGTGTCCTGGATGAGAGACGGTACAGTTGTGAAGACTGATGTGACCTCAACTGAGGAGATGCCTAACGGAGACTGGTACTACCAGATTCACTCGCACCTGGAGTACACTCCCAAATCTGGAGAGAAGATCTCCTGTGTGGTGGATCACGCCGGCTTAACTAAATCCATCATCGTAGACTGG-----------------------------------------------------------------------------------------------------------------------------------------------------------------------GATCCCGCTATGCCTGAGTCTGACAGGAATAAAATCGCCATCGGGGCGTCTGGTCTGGTGCTGGGGATCATCATAGCAGCTGCTGGACTCATTTATTACAAGAAGAAATCAACAGGGAGGATCCTGGTACCAAATC

>Rhsi-DAB1*05:14

CTGATGCTGTCTGCTTTTACTGGAGCA-----------------------------------------------------------------------------------------------------------------------------------------------------------------------------------------------------------------------GCTAATGGATACTACAATTCTCGGTGGAATAAATGCATCCACAGCTCCCGGGATTTCAGCGACATGGTGTCCATTGATAACTATATCTTCAATAAAGATGTGGTTGTACAGTTCAATAGCACTGTGGGAGAGTTTGTGGGGTACACTGAACTTGGAGTAAGTAGTGCACGGAATTGGAACAGCGATCCCAACCGTCTGCAGCAAGAGAGAGCTGAGTTGGAGAGATACTGCAAACATAATGCTGAAATAAGACAGGCATCTATCGCT---GATAAAACA------------------------------------------------------------------------------------------------------------------------------------------------------------------------------------------------------------------------------------------------------------------------------------------------------------------------------------------------------------------------------------------------GTGGCACCAAAGGTCAAGCTCAATTCAGTGACGCAGGCCGGCGGCAGACATCCTGCTGTACTGATGTGCAGCGCTTACCGCTTCTACCCGCACTGGATCAAAGTGTCCTGGATGAGAGACGGTACAGTTGTGAAGACTGATGTGACCTCAACTGAGGAGATGCCTAACGGAGACTGGTACTACCAGATTCACTCGCACCTGGAGTACACTCCCAAATCTGGAGAGAAGATCTCCTGTGTGGTGGATCACGCCGGCTTAACTAAATCCATCATCGTAGACTGG-----------------------------------------------------------------------------------------------------------------------------------------------------------------------GATCCCGCTATGCCTGAGTCTGACAGGAATAAAATCGCCATCGGGGCGTCTGGTCTGGTGCTGGGGATCATCATAGCAGCTGCTGGACTCATTTATTACAAGAAGAAATCAACAGGGAGGATCCTGGTACCAA

>Rhsi-DAB1*05:15

CTGATGCTGTCTGCTTTCACCGGAGCA-----------------------------------------------------------------------------------------------------------------------------------------------------------------------------------------------------------------------GCTAATGGATACTACAATTCTCGGTGGAATAAATGCATTCACAGCTCCCGGGATTTCAGCGACATGGTGTTCATTGATAACTATATCTTCAATAAAGATGTGGTTGTACAGTTCAATAGCACTGTGGGAGAGTTTGTGGGGTACACTGAACTTGGAGTAAGTAGTGCACGGAATTGGAACAGCGATCCCAACCGTCTGCAGCAAGAGAGAGCTGAGTTGGAGAGATACTGCAAACATAATGCTGAAATCAGACAGGCATCTATCGCT---GATAAAACA------------------------------------------------------------------------------------------------------------------------------------------------------------------------------------------------------------------------------------------------------------------------------------------------------------------------------------------------------------------------------------------------GTGGCACCAAAGGTCAAGCTCAGTTCAGTGACGCAGGCCGGCGGCAGACATCCTGCTGTACTGATGTGCAGCGCTTACCGCTTCTACCCGCACTGGATCAAAGTGTCCTGGATGAGAGACGGTACAGTTGTGAAGACTGATGTGACCTCAACTGAGGAGATGCCTAACGGAGACTGGTACTACCAGATTCACTCGCACCTGGAGTACACTCCCAAATCTGGAGAGAAGATCTCCTGTGTGGTGGATCACGCCGGCTTAACTAAATCCATCATCGTAGACTGG-----------------------------------------------------------------------------------------------------------------------------------------------------------------------GATCCCGCTATGCCTGAGTCTGACAGGAATAAAATCGCCATCGGGGCGTCTGGTCTGGTGCTGGGGATCATCATAGCAGCTGCT-GACTCATTTATTACAAGAAGAAATCAACAGGGAGGATCCTGGTACCAA

>Rhsi-DAB1*05:16

CTGATGCTGTCTACATTCACTGGAGCA-----------------------------------------------------------------------------------------------------------------------------------------------------------------------------------------------------------------------GCTAATGGATACTACAATTCTCGGTGGAATAAATGCATCCACAGCTCCCGGGATTTCAGCGACATGGTGTTCATTGATAGCTATATCTTCAATAAAGATGTGGTTGTACAGTTCAATAGCACTGTGGGAGAGTTTGTGGGGTACACTGAACTTGGAGTAAGTAGTGCACGGAATTGGAACAGCGATCCCAACCGTCTGCAGCAAGAGAGAGCTGAGTTGGAGAGATACTGCAAACATAATGCTGAAATCAGACAGGCATCTATCGCT---GATAAAACA------------------------------------------------------------------------------------------------------------------------------------------------------------------------------------------------------------------------------------------------------------------------------------------------------------------------------------------------------------------------------------------------GTGGCACCAAAGGTCAAGCTCAGTTCAGTGACGCAGGCCGGCGGCAGACATCCTGCTGTACTGATGTGCAGCGCTTACCGCTTCTACCCGCACTGGATCAAAGTGTCCTGGATGAGAGACGGTACAGTTGTGAAGACTGATGTGACCTCAACTGAGGAGATGCCTAACGGAGACTGGTACTACCAGATTCACTCGCACCTGGAGTACACTCCCAAATCTGGAGAGAAGATCTCCTGTGTGGTGGATCACGCCGGCTTAACTAAATCCATCATCGTAGACTGG-----------------------------------------------------------------------------------------------------------------------------------------------------------------------GATCCCGCTATGCCTGAGTCTGACAGGAATAAAATCGCCATCGGGGCGTCTGGTCTGGTGCTGGGGATCATCATAGCAGCTGCTGGACTCATTTATTACAAGAAGAAATCAACAGGGAGGATCCTGGTACCAA

>Rhsi-DAB1*05:17

CTGATGCTGTCTACTTTTACTGGAGCA-----------------------------------------------------------------------------------------------------------------------------------------------------------------------------------------------------------------------GCTAATGGATACTACAATTCTCGGTGGAATAAATGCATCCACAGCTCCCGGGATTTCAGCGACATGGTGTTCATTGATAACTATATCTTCAATAAAGATGTGGTTGTACAGTCCAATAGCACTGTGGGAGAGTTTGTGGGGTACACTGAACTTGGAGTAAGTAGTGCACGGAATTGGAACAGCGATCCCAACCGTCTGCAGCAAGAGAGAGCTGAGTTGGAGAGATACTGCAAACATAATGCTGAAATCAGACAGGCATCTATCGCT---GATAAAACA------------------------------------------------------------------------------------------------------------------------------------------------------------------------------------------------------------------------------------------------------------------------------------------------------------------------------------------------------------------------------------------------GTGGCACCAAAGGTCAAGCTCAGTTCAGTGACGCAGGCCGGCGGCAGACATCCTGCTGTACTGATGTGCAGCGCTTACCGCTTCTACCCGCACTGGATCAAAGTGTCCTGGATGAGAGACGGTACAGTTGTGAAGACTGATGTGACCTCAACTGAGGAGATGCCTAACGGAGACTGGTACTACCAGATTCACTCGCACCTGGAGTACACTCCCAAATCTGGAGAGAAGATCTCCTGTGTGGTGGATCACGCCGGCTTAACTAAATCCATCATCGTAGACTGG-----------------------------------------------------------------------------------------------------------------------------------------------------------------------GATCCCGCTATGCCTGAGTCTGACAGGAATAAAATCGCCATCGGGGCGTCTGGTCTGGTGCTGGGGATCATCATAGCAGCTGCTGGATTCATTTATTACAAGAAGAAATCAACAGGGAGGATCCTGGTACCAAATC

>Rhsi-DAB1*05:18

CTGATGCTGTCTGCTTTCACCGGAGCA-----------------------------------------------------------------------------------------------------------------------------------------------------------------------------------------------------------------------GCTAATGGATACTACAATTCTCGGTGGAATAAATGCATCCACAGCTCCCGGGATTTCAGCGACATGGTGTTCATTGATAACTACATCTTCAATAAAGATGTGGTTGTACAGTTCAATAGCACTGTGGGAGAGTTTGTGGGGTACACTGAACTTGGAGTAAGTAGTGCACGGAATTGGAACTGCGATCCCAACCGTCTGCAGCAAGAGAGAGCTGAGTTGGAGAGATACTGCAAACATAATGCTGAAATCAGACAGGCATCTATCGCT---GATAAAACA------------------------------------------------------------------------------------------------------------------------------------------------------------------------------------------------------------------------------------------------------------------------------------------------------------------------------------------------------------------------------------------------GTGGCACCAAAGGTCAAGCTCAGTTCAGTGACGCAGGCCGGCGGCAGACATCCTGCTGTACTGATGTGCAGCGCTTACCGCTTCTACCCGCACTGGATCAAAGTGTCCTGGATGAGAGACGGTACAGTTGTGAAGACTGATGTGACCTCAACTGAGGAGATGCCTAACGGAGACTGGTACTACCAGATTCACTCGCACCTGGAGTACACTCCCAAATCTGGAGAGAAGATCTCCTGTGTGGTGGATCACGCCGGCTTAACTAAACCCATCATCGTAGACTGG-----------------------------------------------------------------------------------------------------------------------------------------------------------------------GATCCCGCTATGCCTGAGTCTGACAGGAATAAAATCGCCATCGGGGCGTCTGGTCAGGTGCTGGGGATCATCATAGCAGCTGCTGGACTCATTTATTACAAGAAGAAATCAACAGGGAGGATCCTGGTACCAAATC

>Rhsi-DAB1*05:19

CTGATGCTGTCTACTTTTACCGGAGCA-----------------------------------------------------------------------------------------------------------------------------------------------------------------------------------------------------------------------GCTAATGGATACTACAATTCTCGGTGGAATAAATGCATCCACAGCTCCCGGGATTTCAGCGACATGGTGTTCATTGATAACTATATCTTCAATAAAGATGTGGTTATACAGTTCAATAGCACTGTGGGAGAGTTTGTGGGGTACACTGAACTTGGAGTAAGTAGTGCACGGAATTGGAACAGCGATCCCAACCGTCTGCAGCAAGAGAGAGCTGAGTTGGAGAGATACTGCAAACATAATGCTGAAATCAGACAGGCATCTATCGCT---GATAAAACA------------------------------------------------------------------------------------------------------------------------------------------------------------------------------------------------------------------------------------------------------------------------------------------------------------------------------------------------------------------------------------------------GTGGCACCAAAGGTCAAGCTCAGTTCAGTGACGCAGGCCGGCGGCAGACATCCTGCTGTACTGATGTGCAGCGCTTACCGCTTCTACCCGCACTGGATCAAAGTGTCCTGGATGAGAGACGGTACAGTTGTGAAGACTGATGTGACCTCAACTGAGGAGATGCCTAACGGAGACTGGTACTACCAGATTCACTCGCACCTGGAGTACACTCCCAAATCTGGAGAGAAGATCTCCTGTGTGGTGGATCACGCCGGCTTAACTAAATCCATCATCGTAGACTGG-----------------------------------------------------------------------------------------------------------------------------------------------------------------------GATCCCGCTATGCCTGAGTCTGACAGGAATAAAATCGCCATCGGGGCGTCTGGTCTGGTGCTGGGGATCATCATAGCAGCTGCTGGACTCATTTATTACAAGAAGAAATCAACAGGGAGGATCCTGGTACCAAATC

>Rhsi-DAB1*06:01

CTGATGCTGTCTGCTTTTACTGGATCA-----------------------------------------------------------------------------------------------------------------------------------------------------------------------------------------------------------------------GCTCATGGATACTACTATTCTCGGTGGGCTAAATGCATCCACAGCTCCCGGGATTTCAGCGACATGGTGTTTATTGATAACTATGTCTTCAATAAAGATGTGTTCATACACTTCAACAGCACTGTGGGAGAGTTTGCGGGGTACACTGAACTTGGAGTATATAATGCACGGAATTGGAACAGCGATCCCAACTTTCTGCAGTCAGAGAGAGCTGAGGTGGAGAGATACTGCAAACATAATGCTGAACTCTATCAGGCAGCTATCGCT---GATAAAACA------------------------------------------------------------------------------------------------------------------------------------------------------------------------------------------------------------------------------------------------------------------------------------------------------------------------------------------------------------------------------------------------GTGGCACCAAAGGTCAAGCTCAGTTCAGTGATGCAGGCCAGCGGCAGTCATCCTGCTGTACTGATGTGCAGCGCTTACCGCTTCTACCCACACTGGATCAAAGTGTCCTGGATGAGGGATGGTAAAGTTGTGAAGACTGACGTGACCTCAACTGAGGAGATGCCTAACGGAGACTGGTACTACCAGATTCACTCGCACCTGGAATACACTCCCAAATCTGGAGAGAAGATTTCCTGTGTGGTGGATCACGCCGGCTTAACTAAATCCATCATCGTAGACTGG-----------------------------------------------------------------------------------------------------------------------------------------------------------------------GATCCCGCTATGCCAGAGTCTGAGAGGAATAAAATCGCCATCGGAGCGTCTGGTCTGGTGCTGGGGATCATCATAGCAGCTGCTGGACTCATTTACTACAAGAAGAAATCAACAGGGAGGATCCTGGTACCAAATC

>Rhsi-DAB1*06:02

CTGATGCTGTCTACTTTCACTGGATCA-----------------------------------------------------------------------------------------------------------------------------------------------------------------------------------------------------------------------GCTCATGGATACTACTATTCTCGGTGGGCTAAATGCATCCACAGCTCCCGGGATTTCAGCGACATGGTGTTTATTGATAACTATGTCTTCAATAAAGATGTGTTCATACACTCCAACAGCACTGTGGGAGGGTTTGTGGGGTACACTGAACTTGGAGTATATAATGCACGGAATTGGAACAGCGATCCCAACTTTCTGCAGTCAGAGAGAGCTGAGGTGGAGAGATACTGCAAACATAATGCTGAACTCTATCAGGCAGCTATCGCT---GATAAAACA------------------------------------------------------------------------------------------------------------------------------------------------------------------------------------------------------------------------------------------------------------------------------------------------------------------------------------------------------------------------------------------------GTGGCACCAAAGGTCAAGCTCAGTTCAGTGATGCAGGCCAGCGGCAGTCATCCTGCTGTACTGATGTGCAGCGCTTACCGCTTCTACCCACACTGGATCAAAGTGTCCTGGATGAGGGATGGTAAAGTTGTGAAGACTGACGTGACCTCAACTGAGGAGATGCCTAACGGAGACTGGTACTACCAGATTCACTCGCACCTGGAATACACTCCCAAATCTGGAGAGAAGATTTCCTGTGTGGTGGATCACGCCGGCTTAACTAAATCCATCATCGTAGACTGG-----------------------------------------------------------------------------------------------------------------------------------------------------------------------GATCCCGCTATGCCAGAGTCTGAGAGGAATAAAATCGCCATCGGAGCGTCTGGTCTGGTGCTGGGGATCATCATAGCAGCTGCTGGACTCATTTACTACAAGAAGAAATCAACAGGGAGGATCCTGGTACCAAATC

>Rhsi-DAB1*06:03

CTGATGCTGTCTGCTTTCACTGGATCA-----------------------------------------------------------------------------------------------------------------------------------------------------------------------------------------------------------------------GCTCATGGATACTACTATTCTCGGTGGGCTAAACGCATCCACAGCTCCCGGGATTTCAGCGACATGGTGTTTATTGATAACTATGTCTTCAATAAAGATGTGTTCATACACTTCAACAGCACCGTGGGAGAGTTTGTGGGGTACACTGAACTTGGAGTATATAATGCACGGAATTGGAACAGCGATCCCAACTTTCTGCAGTCAGAGAGAGCTGAGGTGGAGAGATACTGCAAACATAATGCTGAACTCTATCAGGCAGCTATCGCT---GATAAAACA------------------------------------------------------------------------------------------------------------------------------------------------------------------------------------------------------------------------------------------------------------------------------------------------------------------------------------------------------------------------------------------------GTGGCACCAAAGGTCAAGCTCAGTTCAGTGATGCAGGCCAGCGGCAGTCATCCTGCTGTACTGATGTGCAGCGCTTACCGCTTCTACCCACACTGGATCAAAGTGTCCTGGATGAGGGATGGTAAAGTTGTGAAGACTGACGTGACCTCAACTGAGGAGATGCCTAACGGAGACTGGTACTACCAGATTCACTCGCACCTGGAATACACTCCCAAATCTGGAGAGAAGATTTCCTGTGTGGTGGATCACGCCGGCTTAACTAAATCCATCATCGTAGACTGG-----------------------------------------------------------------------------------------------------------------------------------------------------------------------GATCCCGCTATGCCAGAGTCTGAGAGGAATAAAATCGCCATCGGAGCGTCTGGTCTGGTGCTGGGGATCATCATAGCAGCTGCTGGACTCATTTACTACAAGAAGAAATCAACAGGGAGGATCCTGGTACCAAATC

>Rhsi-DAB1*06:04

CTGATGCTGTCTACATTCACCGGATCA-----------------------------------------------------------------------------------------------------------------------------------------------------------------------------------------------------------------------GCTCATGGATACTACTATTCTCGGTGGGCTAAATGCATCCACAGCTCCCGGGATTTCAGCGACATGGTGTTTATTGATAACTATGTCTTCAATAAAGATGAGTTCATACACTTCAACAGCACTGTGGGAGAGTTTGTGGGGTACACTGAACTTGGAGTATATAATGCACGGAATTGGAACAGCGATCCCAACTTTCTGCAGTCAGAGAGAGCTGAGGTGGAGAGATACTGCAAACATAATGCTGAACTCTATCAGGCAGCTATCGCT---GATAAAACA------------------------------------------------------------------------------------------------------------------------------------------------------------------------------------------------------------------------------------------------------------------------------------------------------------------------------------------------------------------------------------------------GTGGCACCAAAGGTCAAGCTCAGTCCAGTGATGCAGGCCAGCGGCAGTCATCCTGCTGTACTGATGTGCAGCGCTTACCGCTTCTACCCACACTGGATCAAAGTGTCCTGGATGAGGGATGGTAAAGTTGTGAAGACTGACGTGACCTCAACTGAGGAGATGCCTAACGGAGACTGGTACTACCAGATTCACTCGCACCTGGAATACACTCCCAAATCTGGAGAGAAGATTTCCTGTGTGGTGGATCACGCCGGCTTAACTAAATCCATCATCGTAGACTGG-----------------------------------------------------------------------------------------------------------------------------------------------------------------------GATCCCGCTATGCCAGAGTCTGAGAGGAATAAAATCGCCATCGGAGCGTCTGGTCTGGTGCTGGGGATCATCATAGCAGCTGCTGGACTCATTTACTACAAGAAGAAATCAACAGGGAGGATCCTGGTACCAAATC

>Rhsi-DAB1*06:05

CTGATGCTGTCTGCTTTTACTGGATCA-----------------------------------------------------------------------------------------------------------------------------------------------------------------------------------------------------------------------GCTCATGGATACTACTATTCTCGGTGGGCTAAATGCATCCACAGCTCCCGGGATTTCAGCGACATGGTGTTTATTGGTAACTATGTCTTCAATAAAGATGTGTTCATACACTTCAACAGCACTGTGGGAGAGTTTGTGGGGTACACTGAACTTGGAGTATATAATGCACGGAATTGGAACAGCGATCCCAACTTTCTGCAGTCAGAGAGAGCTGAGGTGGAGAGATACTGCAAACATAATGCTGAACTCTATCAGGCAGCTATCGCT---GATAAAACA------------------------------------------------------------------------------------------------------------------------------------------------------------------------------------------------------------------------------------------------------------------------------------------------------------------------------------------------------------------------------------------------GTGGCACCAAAGGTCAAGCTCAGTTCAGTGATGCAGGCCAGCGGCAGTCATCCTGCTGTACTGATGTGCAGCGCTTACCGCTTCTACCCACACTGGATCAAAGTGTCCTGGATGAGGGATGGTAAAGTTGTGAAGACTGACGTGACCTCAACTGAGGAGATGCCTAACGGAGACTGGTACTACCAGATTCACTCGCACCTGGAATACACTCCCAAATCTGGAGAGAAGATTTCCTGTGTGGTGGATCACGCCGGCTTAACTAAATCCATCATCGTAGACTGG-----------------------------------------------------------------------------------------------------------------------------------------------------------------------GATCCCGCTATGCCAGAGTCTGAGAGGAATAAAATCGCCATCGGAGCGTCTGGTCTGGTGCTGGGGATCATCATAGCAGCTGCTGGACTCATTTACTACAAGAAGAAATCAACAGGGAGGATCCTGGTACCAAATC

>Rhsi-DAB1*06:06

CTGATGCTGTCTACTTTCACCGGATCA-----------------------------------------------------------------------------------------------------------------------------------------------------------------------------------------------------------------------GCTCATGGATACTACTATTCTCGGTGGGCTAAATGCATCCACAGCTCCCGGGATTTCAGCGACATGGTGTTTATTGATAACTATGTCTTCAATAAAGATGTGTTCATACACTTCGACAGCACTGTGGGAGAGTTTGTGGGGTACACTGAACTTGGAGTATATAATGCACGGAATTGGAACAGCGATCCCAACTTTCTGCAGTCAGAGAGAGCTGAGGTGGAGAGATACTGCAAACATAATGCTGAACTCTATCAGGCAGCTATCGCT---GATAAAACA------------------------------------------------------------------------------------------------------------------------------------------------------------------------------------------------------------------------------------------------------------------------------------------------------------------------------------------------------------------------------------------------GTGGCACCAAAGGTCAAGCTCAGTTCAGTGATGCAGGCCAGCGGCAGTCATCCTGCTGTACTGATGTGCAGCGCTTACCGCTTCTACCCACACTGGATCAAAGTGTCCTGGATGAGGGATGATAAAGTTGTGAAGACTGACGTGACCTCAACTGAGGAGATGCCTAACGGAGACTGGTACTACCAGATTCACTCGCACCTGGAATACACTCCCAAATCTGGAGAGAAGATTTCCTGTGTGGTGGATCACGCCGGCTTAACTAAATCCATCATCGTAGACTGG-----------------------------------------------------------------------------------------------------------------------------------------------------------------------GATCCCGCTATGCCAGAGTCTGAGAGGAATAAAATCGCCATCGGAGCGTCTGGTCTGGTGCTGGGGATCATCATAGCAGCTGCTGGACTCATTTACTACAAGAAGAAATCAACAGGGAGGATCCTGGTACCAA

>Rhsi-DAB1*06:07

CTGATGCTGTCTGCTTTTACTGGATCA-----------------------------------------------------------------------------------------------------------------------------------------------------------------------------------------------------------------------GCTCATGGATACTACTATTCTCGGTGGGCTAAATGCAACCACAGCTCCCGGGATTTCAGCGACATGGTGTTTATTGATAACTATGTCTTCAATAAAGATGTGTTCATACACTTCAACAGCACTGTGGGAGAGTTTGTGGGGTACACTGAACTTGGAGTATATAATGCACGGAATTGGAACAGCGATCCCAACTTTCTGCAGTCAGAGAGAGCTGAGGTGGAGAGATACTGCAAACATAATGCTGAACTCTATCAGGCAGCTATCGCT---GATAAAACA------------------------------------------------------------------------------------------------------------------------------------------------------------------------------------------------------------------------------------------------------------------------------------------------------------------------------------------------------------------------------------------------GTGGCACCAAAGGTCAAGCTCAGTTCAGTGATGCAGGCCAGCGGCAGTCATCCTGCTGTACTGATGTGCAGCGCTTACCGCTTCTACCCACACTGGATCAAAGTGTCCTGGATGAGGGATGGTAAAGTTGTGAAGACTGACGTGACCTCAACTGAGGAGATGCCTAACGGAGACTGGTACTACCAGATTCACTCGCACCTGGAATACACTCCCAAATCTGGAGAGAAGATTTCCTGTGTGGTGGATCACGCCGGCTTAACTAAATCCATCATCGTAGACTGG-----------------------------------------------------------------------------------------------------------------------------------------------------------------------GATCCCGCTATGCCAGAGTCTGAGAGGAATAAAATCGCCATCGGAGCGTCTGGTCTGGTGCTGGGGATCATCATAGCAGCTGCTGGACTCATTTACTACAAGAAGAAATCAACAGGGAGGATCCTGGTACCAA

>Rhsi-DAB1*06:08

CTGATGCTGTCTACTTTCACCGGATCA-----------------------------------------------------------------------------------------------------------------------------------------------------------------------------------------------------------------------GCTCATGGATACTACTATTCTCGGTGGGCTAAATGCATCCACAGCTCCCGGGATTTCAGCGACATGGTGTTTATTGATAACCATGTCTTCAATAAAGATGTGTTCATACACTTCAACAGCACTGTGGGAGAGTTTGTGGGGTACACTGAACTTGGAGTATATAATGCACGGAATTGGAACAGCGATCCCAACTTTCTGCAGTCAGAGAGAGCTGAGGTGGAGAGATACTGCAAACATAATGCTGAACTCTATCAGGCAGCTATCGCT---GATAAAACA------------------------------------------------------------------------------------------------------------------------------------------------------------------------------------------------------------------------------------------------------------------------------------------------------------------------------------------------------------------------------------------------GTGGCACCAAAGGTCAAGCTCAGTTCAGTGATGCAGGCCAGCGGCAGTCATCCTGCTGTACTGATGTGCAGCGCTTACCGCTTCTACCCACACTGGATCAAAGTGTCCTGGATGAGGGATGGTAAAGTTGTGAAGACTGACGTGACCTCAACTGAGGAGATGCCTAACGGAGACTGGTACTACCAGATTCACTCGCACCTGGAATACACTCCCAAATCTGGAGAGAAGATTTCCTGTGTGGTGGATCACGCCGGCTTAACTAAATCCATCATCGTAGACTGG-----------------------------------------------------------------------------------------------------------------------------------------------------------------------GATCCCGCTATGCCAGAGTCTGAGAGGAATAAAATCGCCATCGGAGCGTCTGGTCTGGTGCTGGGGATCATCATAGCAGCTGCTGGACTCATTTACTACAAGAAGAAATCAACAGGGAGGATCCTGGTACCAA

>Rhsi-DAB1*06:09

CTGATGCTGTCTACTTTCACTGGATCA-----------------------------------------------------------------------------------------------------------------------------------------------------------------------------------------------------------------------GCTCATGGATACTACTATTCTCGGTGGGCTAAATGCATCCACAGCTCCCGGGATTTCAGCGACATGGTGTTCATTGATAACTATGTCTTCAATAAAGATGTGTTCATACACTTCAACAGCACTGTGGGAGAGTWTGTGGGGTACACTGAACTTGGAGTATATAATGCACGGAATTGGAACAGCGATCCCAACTTTCTGCAGTCAGAGAGAGCTGAGGTGGAGAGATACTGCAAACATAATGCTGAACTCTATCAGGCAGCTATCGCT---GATAAAACA------------------------------------------------------------------------------------------------------------------------------------------------------------------------------------------------------------------------------------------------------------------------------------------------------------------------------------------------------------------------------------------------GTGGCACCAAAGGTCAAGCTCAGTTCAGTGATGCAGGCCAACGGCAGTCATCCTGCTGTACTGATGTGCAGCGCTTACCGCTTCTACCCACACTGGATCAAAGTGTCCTGGATGAGGGATGGTAAAGTTGTGAAGACTGACGTGACCTCAACTGAGGAGATGCCTAACGGAGACTGGTACTACCAGATTCACTCGCTCCTGGAATACGCTCCCAAATCTGGAGAGAAGATTTCCTGTGTGGTGGATCACGCCGGCTTAACTAAATCCATCATCGTAGACTGG-----------------------------------------------------------------------------------------------------------------------------------------------------------------------GATCCCGCTATGCCAGAGTCTGASAGGAATAAAATCGCCATCGGAGCGTCTGGTCTGGTGCTGGGGATCATCATAGCAGCTGCTGGACTCATTTACTACAAGAAGAGATCAACAGGGAGGATCCTGGTACCAA

>Rhsi-DAB1*06:10

CTGATGCTGTCTGCTTTTACTGGATCA-----------------------------------------------------------------------------------------------------------------------------------------------------------------------------------------------------------------------GCTCATGGATACTACTATTCTCGGTGGGCTAAATGCATCCACAGCTCCCGGGATTTCAGCGACATGGTGTTTATTGATAACTATGTCTTCAATAAAGATGTGCTCATACACTTCAACAGCACTGTGGGAGAGTTTGTGGGGTACACTGAACTTGGAGTATATAATGCACGGAATTGGAACAGCGATCCCAACTTTCTGCAGTCAGAGAGAGCTGAGGTGGAGAGATACTGCAAACATAATGCTGAACTCTATCAGGCAGCTATCGCT---GATAAAACA------------------------------------------------------------------------------------------------------------------------------------------------------------------------------------------------------------------------------------------------------------------------------------------------------------------------------------------------------------------------------------------------GTGGCACCAAAGGTCAAGCTCAGTTCAGTGATGCAGGCCAGCGGCAGTCATCCTGCTGTACTGATGTGCAGCGCTTACCGCTTCTACCCACACTGGATCAAAGTGTCCTGGATGAGGGATGGTAAAGTTGTGAAGACTGACGTGACCTCAACTGAGGAGATGCCTAACGGAGACTGGTACTACCAGATTCACTCGCACCTGGAATACACTCCCAAATCTGGAGAGAAGATTTCCTGTGTGGTGGATCACGCCGGCTTAACTAAATCCATCATCGTAGACTGG-----------------------------------------------------------------------------------------------------------------------------------------------------------------------GATCCCGCTATGCCAGAGTCTGAGAGGAATAAAATCGCCATCGGAGCGTCTGGTCTGGTGCTGGGGATCATCATAGCAGCTGCTGGACTCATTTACTACAAGAAGAAATCAACAGGGAGGATCCTGGTACCAA

>Rhsi-DAB1*06:11

CTGATGCTGTCTGCTTTTACTGGATCA-----------------------------------------------------------------------------------------------------------------------------------------------------------------------------------------------------------------------GCTCATGGATACTACTATTCTCGGTGGGCTAAATGCATCCACAGCTCCCGGGATTTCAGCGACATGGTGTTTATTGATAACTATGTCTTCAGTAAAGATGTGTTCATACACTTCAACAGCACTGTGGTAGAGTTTGTGGGGTACACTGAACTTGGAGTATATAATGCACGGAATTGGAACAGCGATCCCAACTTTCTGCAGTCAGAGAGAGCTGAGGTGGAGAGATACTGCAAACATAATGCTGAACTCTATCAGGCAGCTATCGCT---GATAAAACA------------------------------------------------------------------------------------------------------------------------------------------------------------------------------------------------------------------------------------------------------------------------------------------------------------------------------------------------------------------------------------------------GTGGCACCAAAGGTCAAGCTCAGTTCAGTGATGCAGGCCAGCGGCAGTCATCCTGCTGTACTGATGTGCAGCGCTTACCGCTTCTACCCACACTGGATCAAAGTGTCCTGGATGAGGGATGGTAAAGTTGTGAAGACTGACGTGACCTCAACTGAGGAGATGCCTAACGGAGACTGGTACTATCAGATTCACTCGCACCTGGAATACACTCCCAAATCTGGAGAGAAGATCTCCTGTGTGGTGGATCACGCCGGCTTAACTAAATCCATCATCGTAGACTGG-----------------------------------------------------------------------------------------------------------------------------------------------------------------------GATCCCGCTATGCCAGAGTCTGACAGGAATAAAATCGCCATCGGAGCGTCTGGTCTGGTGCTGGGGATCATCATAGCAGCTGCTGGACTCATTTACTACAAGAAGAAATCAACAGGGAGGATCCTGGTACCAA

>Rhsi-DAB1*06:12

CTGATGCTGTCTGCTTTTACTGGATCA-----------------------------------------------------------------------------------------------------------------------------------------------------------------------------------------------------------------------GCTCATGGATACTACTATTCTCGGTGGGCTAAATGCATCCACAGCTCCCGGGATTTCAGCGACATGGTGTTTATTGATAACTATGTCTTCAATAAAGATGTGTTCCTACACTTCAACAGCACTGTGGGAGAGTTTGTGGGGTACACTGAACTTGGAGTATATAATGCACGGAATTGGAACAGCGATCCCAACTTTCTGCAGTCAGAGAGAGCTGAGGTGGAGAGATACTGCAAACATAATGCTGAACTCTATCAGGCAGCTATCGCT---GATAAAACA------------------------------------------------------------------------------------------------------------------------------------------------------------------------------------------------------------------------------------------------------------------------------------------------------------------------------------------------------------------------------------------------GTGGCACCAAAGGTCAAGCTCAGTTCAGTGATGCAGGCCAGCGGCAGTCATCCTGCTGTACTGATGTGCAGCGCTTACCGCTTCTACCCACACTGGATCAAAGTGTCCTGGATGAGGGATGGTAAAGTTGTGAAGACTGACGTGGCCTCAACTGAGGAGATGCCTAACGGAGACTGGTACTATCAGATTCACTCGCACCTGGAATACACTCCCAAATCTGGAGAGAAGATCTCCTGTGTGGTGGATCACGCCGGCTTAACTAAATCCATCATCGTAGACTGG-----------------------------------------------------------------------------------------------------------------------------------------------------------------------GATCCCGCTATGCCAGAGTCTGACAGGAATAAAATCGCCATCGGAGCGTCTGGTCTGGTGCTGGGGATCATCATAGCAGCTGCTGGACCCATTTACTACAAGAAGAAATCAACAGGGAGGATCCTGGTACCAA

>Rhsi-DAB1*07:01

CTGATGCTGTCTGCTTTTACCGGATCA-----------------------------------------------------------------------------------------------------------------------------------------------------------------------------------------------------------------------GCTCATGGATACTACTTTTCTCGGTGGACTAAATGCATCCACAGCTCCCGGGATTTCAGCGACATGGTGTACATTGATAACTATATCTTCAATAAAGATGTGTTCATACAGTTCAACAGCACTGTGGGAGAGTTTGTGGGGTACACTGAACTTGGAGTATATAATGCACGGAATTGGAACAGCGATCCCAACCGTCTGCAGGAAGAGAGCGCTAGAGTGGAGACATACTGCAAACATAATGCTGAAATCTATCAGGCAGCTATCGCT---GATAAAACA------------------------------------------------------------------------------------------------------------------------------------------------------------------------------------------------------------------------------------------------------------------------------------------------------------------------------------------------------------------------------------------------GTGGCACCAAAGGTCAAGCTCAGTTCAGTGATGCAGGTCAGCGGCAGTCATCCTGCTGTACTGATGTGCAGCGCTTACCGCTTCTACCCACACTGGATCAAAGTGTCCTGGATGAGGGATGGTAAAGTTGTGAAGACTGACGTGACCTCAACTGAGGAGATGCCTAACGGAGACTGGTACTACCAGATTCACTCGCACCTGGAATACACTCCCAAATCTGGAGAGAAGATCTCCTGTGTGGTGGATCACGCCGGCTTAACTAAATCCATCATCGTAGACTGG-----------------------------------------------------------------------------------------------------------------------------------------------------------------------GATCCCGCTATGCCAGAGTCTGACAGGAATAAAATCGCCATCGGGGCATCAGGTCTGGTGCTGGGGATCATCATGGTAGCTGCTGGACTCATTTACTACAAGAAGAAATCAACAGGGAGGATCCTGGTACCAAA

>Rhsi-DAB1*07:02

CTGATGCTGTCTGCATTTACCGGATCA-----------------------------------------------------------------------------------------------------------------------------------------------------------------------------------------------------------------------GCTCATGGATACTACTTTTCTCGGTGGACTAAATGCATCCACAGCTCCCGGGATTTCAGCGACATGGCGTACATTGATAACTATATCTTCAATAAAGATGTGTTCATACAGTTCAACAGCACTGTGGGAGAGTTTGTGGGGTACACTGAACTTGGAGTATATAATGCACGGAATTGGAACAGCGATCCCAACCGCCTGCAGTCAGAGAGAGCTAGAGTGGAGACATACTGCAAACATAATGCTGAAATCTATCAGGCAGCTATCGCT---GATAAAACA------------------------------------------------------------------------------------------------------------------------------------------------------------------------------------------------------------------------------------------------------------------------------------------------------------------------------------------------------------------------------------------------GTGGCACCAAAGGTCAAGCTCAGTTCAGTGATGCAGGCCAGCGGCAGTCATCCTGCTGTACTGATGTGCAGCGCTTACCGCTTCTACCCACACTGGATCAAAGTGTCCTGGATGAGGGATGGTAAAGTTGTGAAGACTGACGTGACCTCAACTGAGGAGATGCCTAACGGAGACTGGTACTACCAGATCCACTCGCACCTGGAATACACTCCCAAATCTGGAGAGAAGATCTCCTGTGTAGTGGATCACGCCGGCTTAACTAAATCCATCATCGTAGACTGG-----------------------------------------------------------------------------------------------------------------------------------------------------------------------GATCCCGCTATGCCAGAGTCTGACAGGAATAAAATCGCCATCGGAGCGTCTGGTCTGGTGCTGGGGATCATCATAGCAGCTGCTGGACTCATTTACTACAAGAAGAAATCAACAGGGAGGATCCTGGTACCAAA

>Rhsi-DAB1*07:03

CTGATGCTGTCTGCTTTTACCGGATCA-----------------------------------------------------------------------------------------------------------------------------------------------------------------------------------------------------------------------GCTCATGGATACTACTTTTCTCGGTGGACTAAATGCATCCGCAGCTCCCGGGATTTCAGCGACATGGTGTACATTGATAACTATATCTTCAATAAAGATGTGTTCATACAGTTCAACAGCACTGTGGGAGAGTTTGTGGGGTACACTGAACTTGGAGTATATAATGCACGGAATTGGAACAGCGATCCCAACCGTCTGCAGTCAGAGAGAGCTAGAGTGGAGACATACTGCAAACATAATGCTGAAATCTATCAGGCAGCTATCGCT---GATAAAACA------------------------------------------------------------------------------------------------------------------------------------------------------------------------------------------------------------------------------------------------------------------------------------------------------------------------------------------------------------------------------------------------GTGGCACCAAAGGTCAAGCTCAGTTCAGTGATGCAGGCCAGCGGCAGTCATCCTGCTGTACTGATGTGCAGCGCTTACCGCTTCTACCCACACTGGATCAAAGTGTCCTGGATGAGGGATGGTAAAGTTGTGAAGACTGACGTGACCTCAACTGAGGAGATGCCTAACGGAGACTGGTACTACCAGATTCACTCGCACCTGGAATACACTCCCAAATCTGGAGAGAAGATCTCCTGTGTGGTGGATCACGCCGGCTTAACTAAATCCATCATCGTAGACTGG-----------------------------------------------------------------------------------------------------------------------------------------------------------------------GATCCCGCTATGCCAGAGTCTGACAGGAATAAAATCGCCATCGGAGCGTCTGGTCTGGTGCTGGGGATCATCATAGCAGCTGCTGGACTCATTTACTACAAGAAGAAATCAACAGGGAGGATCCTGGTACCAAA

>Rhsi-DAB1*07:04

CTGATGCTGTCTACATTTACCGGATCA-----------------------------------------------------------------------------------------------------------------------------------------------------------------------------------------------------------------------GCTCATGGATACTACTTTTCTCGGTGGACTAAATGCATCCACAGCTCTCGGGATTTCAGCGACATGGAGTACATTGATAACTATATCTTCAATAAAGATGTGTTCATACAGTTCAACAGCACTGTGGGAGAGTTTGTGGGGTACACTGAACTTGGAGTATATAATGCACGGAATTGGAACAGCGATCCCAACCGTCTGCAGGAAGAGAGCGCTAGAGTGGAGACATACTGCAAACATAATGCTGAAATCTATCAGGCAGCTATCGCT---GATAAAACA------------------------------------------------------------------------------------------------------------------------------------------------------------------------------------------------------------------------------------------------------------------------------------------------------------------------------------------------------------------------------------------------GTGGCACCAAAGGTCAAGCTCAGTTCAGTGATGCAGGCCAGCGGCAGTCATCCTGCTGTACTGATGTGCAGCGCTTACCGCTTCTACCCACACTGGATCAAAGTGTCCTGGATGAGGGATGGTAAAGTTGTGAAGACTGACGTGACCTCAACTGAGGAGATGCCTAACGGAGACTGGTACTACCAAATTCACTCGCACCTGGAATACACTCCCAAATCTGGAGAGAAGATCTCCTGTGTGGTGGATCACGCCGGCTTAACTAAATCCATCATCGTAGACTGG-----------------------------------------------------------------------------------------------------------------------------------------------------------------------GATCCCGCTATGCCAGAGTCTGACAGGAATAAAATCGCCATCGGGGCATCAGGTCTGGTGCTGGGGATCATCATAGCAGCTGCTGGACTCATTTACTACAAGAAGAAATCAACAGGGAGGATCCTGGTACCAAATC

>Rhsi-DAB1*07:05

CTGATGCTGTCTACATTTACCGGATCA-----------------------------------------------------------------------------------------------------------------------------------------------------------------------------------------------------------------------GCTCATGGATACTACTTTTCTCGGTGGACTAAATGCATCCACAGCTCCCGGGATTTCAGCGACATGGTGTACATTGATAACTATATCTTCAATAAAGATGTGTTCATACAGTTCAACAGCACTGCGGGAGAGTTTGTGGGGTACACTGAACTTGGAGTATATAATGCACGGAATTGGAACAGCGATCCCAACCGTCTGCAGGAAGAGAGCGCTAGAGTGGAGACATACTGCAAACATAATGCTGAAATCTATCAGGCAGCTATCGCT---GATAAAACA------------------------------------------------------------------------------------------------------------------------------------------------------------------------------------------------------------------------------------------------------------------------------------------------------------------------------------------------------------------------------------------------GTGGCACCAAAGGTCAAGCTCAGTTCAGTGATGCAGGCCAGCGGCAGTCATCCTGCTGTACTGATGTGCAGCGCTTACCGCTTCTACCCACACTGGATCAAAGTGTCCTGGATGAGGGATGGTAAAGTTGTGAAGACTGACGTGACCTCAACTGAGGAGATGCCTAACGGAGACTGGTACTACCAGATTCACTCGCACCTGGAATACACTCCCAAATCTGGAGAGAAGATCTCCTGTGTGGTGGATCACGCCGGCTTAACTAAATCCATCATCGTAGACTGG-----------------------------------------------------------------------------------------------------------------------------------------------------------------------GATCCCGCTATGCCAGAGTCTGACAGGAATAAAATCGCCATCGGGGCATCAGGTCTGGTGCTGGGGATCATCATAGCAGCTGCTGGACTCATTTACTACAAGAAGAAATCAACAGGGAGGATCCTGGTACCAAATC

>Rhsi-DAB1*07:06

CTGATGCTGTCTACATTTACCGGATCA-----------------------------------------------------------------------------------------------------------------------------------------------------------------------------------------------------------------------GCTCATGGATACTACTTTTCTCGGTGGACTAAATGCATCCACAGCTCCCGGGATTTCAGCGACATGGTGTACAATGATAACTATATCTTCAATAAAGATGTGTTCATACAGTTCAACAGCACTGTGGGAGAGTTTGTGGGGTACACTGAACTTGGAGTATATAATGCACGGAATTGGAACAGCGATCCCAACCGTCTGCAGGAAGAGAGCGCTAGAGTGGAGACATACTGCAAACATAATGCTGAAATCTATCAGGCAGCTATCGCT---GATAAAACA------------------------------------------------------------------------------------------------------------------------------------------------------------------------------------------------------------------------------------------------------------------------------------------------------------------------------------------------------------------------------------------------GTGGCACCAAAGGTCAAGCTCAGTTCAGTGATGCAGGCCAGCGGCAGTCATCCTGCTGTACTGATGTGCAGCGCTTACCGCTTCTACCCACACCGGATCAAAGTGTCCTGGATGAGGGATGGTAAAGTTGTGAAGACTGACGTGACCTCAACTGAGGAGATGCCTAACGGAGACTGGTACTACCAGATTCACTCGCACCTGGAATACACTCCCAAATCTGGAGAGAAGATCTCCTGTGTGGTGGATCACGCCGGCTTAACTAAATCCATCATCGTAGACTGG-----------------------------------------------------------------------------------------------------------------------------------------------------------------------GATCCCGCTATGCCAGAGTCTGACAGGAATAAAATCGCCATCGGGGCATCAGGTCTGGTGCTGGGGATCATCATAGCAGCTGCTGGACTCATTTACTACAAGAAGAAATCAACAGGGAGGATCCTGGTACCAAA

>Rhsi-DAB1*07:07

CTGATGCTGTCTGCATTCACTGGATCA-----------------------------------------------------------------------------------------------------------------------------------------------------------------------------------------------------------------------GCTCATGGATACTACTTTTCTCGGCGGACTAAATGCATCCACAGCTCCCGGGATCTCAGCGACATGGTGTACATTGATAACTATATCTTCAATAAAGATGTGTTCGTACAGTTCAACAGCACTGTGGGAGAGTTTGTGGGGTACACTGAACTTGGAGTATATAATGCACGGAATTGGAACAGCGATCCCAACCGTCTGCAGGAAGAGAGCGCTAGAGTGGAGACATACTGCAAACATAATGCTGAAATCTATCAGGCAGCTATCGCT---GATAAAACA------------------------------------------------------------------------------------------------------------------------------------------------------------------------------------------------------------------------------------------------------------------------------------------------------------------------------------------------------------------------------------------------GTGGCACCAAAGGTCAAGCTCAGTTCAGTGATGCAGGCCAGCGGCAGTCATCCTGCTGTACTGATGTGCAGCGCTTACCGCTTCTACCCACACTGGATCAAAGTGTCCTGGATGAGGGATGGTAAAGTTGTGAAGACTGACGTGACCTCAACTGAGGAGATGCCTAACGGAGACTGGTACTACCAGATTCACTCGCACCTGGAATACACTCCCAAATCTGGAGAGAAGATCTCCTGTGTGGTGGATCACGCCGGCTTAACTAAATCCATCATCGTAGACTGG-----------------------------------------------------------------------------------------------------------------------------------------------------------------------GATCCCGCTATGCCAGAGTCTGACAGGAATAAAATCGCCATCGGGGCATCAGGTCTGGTGCTGGGGATCATCATAGCAGCTGCTGGACTCATTTACTACAAGAAGAAATCAACAGGGAGGATCCTGGTACCAA

>Rhsi-DAB1*07:08

TTGATGCTGTGTGCATTCACCAGATCA-----------------------------------------------------------------------------------------------------------------------------------------------------------------------------------------------------------------------GCTCATGGATACTACTTTTCTCGGTGGACTAAATGCATCCACAGCTCCCGGGATTTCAGCGACATGGTGTACATTGATAACTATATCTTCAATAAAGATGTGTTCATACACTTCAACAGCACTGTGGGAGAGTTTGTGGGGTACACTGAACTTGGAGTATATAATGCACGGAATTGGAACAGCGATCCCAACCGTCTGCAGGAAGAGAGCGCTAGAGTGGAGACATACTGCAAACATAATGCTGAAATCTATCAGGCAGCTATCGCT---GATAAAACA------------------------------------------------------------------------------------------------------------------------------------------------------------------------------------------------------------------------------------------------------------------------------------------------------------------------------------------------------------------------------------------------GTGGCACCAAAGGTCAAGCTCAGTTCAGTGATGCAGGCCAGCGGCAGTCATCCTGCTGTACTGATGTGCAGCGCTTACCGCTTCTACCCACACTGGATCAAAGTGTCCTGGATGAGGGATGGTAAAGTTGTGAAGACTGACGTGACCTCAACTGAGGAGATGCCTAACGGAGACTGGTACTACCAGATTCACTCGCACCTGGAATACACTCCCAAATCTGGAGAGAAGATCTCCTGTGTGGTGGATCACGCCGGCTTAACTAAATCCATCATCGTAGACTGG-----------------------------------------------------------------------------------------------------------------------------------------------------------------------GATCCCGCTATGCCAGAGTCTGACAGGAATAAAATCGCCATCGGGGCATCAGGTCTGGTGCTGGGGATCATCATAGCAGCTGCTGGACTCATTTACTACAAGAAGAAATCAACAGGGAGGATCCTGGTACCATCTTAATGATGGTCAGACTGACTGGAAGAGGATTCAATTTGGGCCACTGATGGAAAACTCGCTTTCTGAACTTTACACTTATGCTTCATTTTTAACAATGGAAACAATTTTGGGAAAATGAAAACAATAACAACAAATCTAATACATTTGTTTGTTATTTTCTTTTTTTATTGAATGTTGATGATGATATAATGATGTTTAAAATATGGAGATTATATATAGAGAAGTAGAGATTATCATTCTTCCTGGCTTCTTTCCAGTACTTTTACTGGATTAACATGCATATAGGTTCATAATGTTATAACACAGTCTTGTAAGTCTTTGTTACTAATAAGTAGCAGTCAGGCTCTGGGTCTGAGAAAGGGTACTCTGAAGTCTTTTTCTGATTACTACTTTAAATTTACCATAAAAAATATATTAAAACTACATTTGTGCACTCTTATATTTAATAGAAAAAACATATTTAGCATAAATTAGACCATCAACAGATTATGTTTTTCCTTTTTATTCATTCTGACAATGATTCTTCTGTATGATTTGTTGACTGCAAAATA

>Rhsi-DAB1*07:09

CTGATGCTGTCTACATTTACTGGATCA-----------------------------------------------------------------------------------------------------------------------------------------------------------------------------------------------------------------------GCTCATGGATACTACTTTTCTCGGTGGACTAAATGCATCCACAGCTCCCGGGATTTCAGCGACATGGTGTACATTGATAACTATATCTACAATAAAGATGTGTTCATACAGTTCAACAGCACTGTGGGAGAGTTTGTGGGGTACACTGAACTTGGAGTATATAATGCACGGAATTGGAACAGCGATCCCAACCGTCTGCAGGAAGAGAGCGCTAGAGTGGAGACATACTGCAAACATAATGCTGAAATCTATCAGGCAGCTATCGCT---GATAAAACA------------------------------------------------------------------------------------------------------------------------------------------------------------------------------------------------------------------------------------------------------------------------------------------------------------------------------------------------------------------------------------------------GTGGCACCAAAGGTCAAGCTCAGTTCAGTGATGCAGGCCAGCGGCAGTCATCCTGCTGTACTGATGTGCAGCGCTTACCGCTTCTACCCACACTGGATCAAAGTGTCCTGGATGAGGGATGGTAAAGTTGTGAAGACTGACGTGACCTCAACTGAGGAGATGCCTAACGGAGACTGGTACTACCAGATTCACTCGCACCTGGAGTACACTCCCAAATCTGGAGAGAAGATCTCCTGTGTGGTGGATCACGCCGGCTTAACTAAATCCATCATCGTAGACTGG-----------------------------------------------------------------------------------------------------------------------------------------------------------------------GATCCCGCTATGCCAGAGTCTGACAGGAATAAAATCGCCATCGGGGCATCAGGTCTGGTGCTGGGGATCATCATAGCAGCTGCTGGACTCATTTACTACAAGAAGAAATCAACAGGGAGGATCCTGGTACCAAATC

>Rhsi-DAB1*08:01

CTGATGCTGTCTGCATTCACTGGAGCA-----------------------------------------------------------------------------------------------------------------------------------------------------------------------------------------------------------------------GCTCATGGATACTACTGGTCTATGTGGTCTAAATGCATCCACAGCTCCCGGGATTTCAGCGACATGGTGTTCATTGATAACTATATCTTCAATAAAGATGTGTTCATACAGTTCAACAGCACTGTGGGAGAGTATGTGGGGTACACTGAACTTGGAGTATATAATGCACGAAGAATGAACAACGATCCCAACCGTCTGCAGCAAGAGAGAGCTGAGGTGGAGAGATACTGCAAACATAATGCTGAAATCTATCAGGCAGCTATCGCT---GATAAAACA------------------------------------------------------------------------------------------------------------------------------------------------------------------------------------------------------------------------------------------------------------------------------------------------------------------------------------------------------------------------------------------------GTGGCACCAAAGGTCAAGCTCAGTTCAATGATGCAGGCCAGCGGCAGTCATCCTGCTGTACTGATGTGCAGCGCTTACCGCTTCTACCCACACTGGATCAAAGTGTCCTGGATGAGGGATGGTAAAGTTGTGAAGACTGACGTGACCTCAACTGAGGAGATGCCTAACGGAGACTGGTACTACCAGATTCACTCGCACCTGGAATACACTCCCAAATCTGGAGAGAAGATCTCCTGTGTGGTGGATCACGCCGGCTTAACTAAATCCATCATCGTAGACTGG-----------------------------------------------------------------------------------------------------------------------------------------------------------------------GATCCCGCTATGCCAGAGTCTGACAGGAATAAAATTGCCATCGGAGCGTCTGGTCTGGTGCTGGGGATCATCATAGCAGCTGCTGGACTCATTTACTACAAGGAGAAATCAACAGGGAGGATCCTGGTACCAAATC

>Rhsi-DAB1*08:02

CTGATGCTGTCTGCTTTTACCGGATCA-----------------------------------------------------------------------------------------------------------------------------------------------------------------------------------------------------------------------GCTCATGGATACTACTGGTCTCGGTGGAATAAATGCATCCACAGCTCCCGGGATTTCAGCGACATGGTGTTCATTGATAACTATATCTTCAATAAAGATGTGTACATACAGTTCAACAGCACTGTGGGAGAGTATGTGGGGTACACTGAACTTGGAGTATATAATGCACGAAGAATGAACAACGATCCCAACTTTCTGCAGCAAGAGAGAGCTGGAGTGGAGACATACTGCAAACATAATGCTGAAATCTATCAGGCAGCTATCGCT---GACAAAACA------------------------------------------------------------------------------------------------------------------------------------------------------------------------------------------------------------------------------------------------------------------------------------------------------------------------------------------------------------------------------------------------GTGGCACCAAAGGTCAAGCTCAGTTCAGTGACGCAGGCCAGCGGCAGTCATCCTGCTGTACTGATGTGCAGCGCTTACCGCTTCTACCCACACTGGATCAAAGTGTCCTGGATGAGGGATGGTAAAGTTGTGAAGACTGACGTGACCTCAACTGAGGAGATGCCTAACGGAGACTGGTACTACCAGATTCACTCGCACCTGGAATACACTCCCAAATCTGGAGAGAAGATCTCCTGTGTGGTGGATCACGCCGGCTTAACTAAATCTATCATCGTAGACTGG-----------------------------------------------------------------------------------------------------------------------------------------------------------------------GATCCCGCTATGCCTGAGTCTGACAGGAATAAAATCGCCATCGGGGCGTCTGGTCTGATGCTGGGGATCATCATAGCAGCTGCTGGACTCATTTACTACAAGAAGAAATCAACAGGGAGGATCCTGGTACCAAATC

>Rhsi-DAB1*08:03

CTGATGCTGTCTGCATTCACCGGAGCA-----------------------------------------------------------------------------------------------------------------------------------------------------------------------------------------------------------------------GCTCATGGATACTACTGGTCTATGTGGTCTAAATGCATCCACAGCTCCCGGGATTTCAGCGACATGGTGCTCATTGATAACTATATCTTCAATAAAGATGTGTTCATACAGTTCAACAGCACTGTGGGAGAGTATGTGGGGTACACTGAACTTGGAGTATATAATGCACGAAGAATGAACAACGATCCCAACCGTCTGCAGCAAGAGAGAGCTGAGGTGGAGAGATACTGCAAACATAATGCTGAAATCTATCAGGCAGCTATCGCT---GATAAAACA------------------------------------------------------------------------------------------------------------------------------------------------------------------------------------------------------------------------------------------------------------------------------------------------------------------------------------------------------------------------------------------------GTGGCACCAAAGGTCAAGCTCAGTTCAATGATGCAGGCCAGCGGCAGTCATCCTGCTGTACTGATGTGCAGCGCTTACCGCTTCTACCCACACTGGATCAAAGTGTCCTGGATGAGGGATGGTAAAGTTGTGAAGACTGACGTGACCTCAACTGAGGAGATGCCTAACGGAGACTGGTACTACCAGATTCACTCGCACCTGGAATACACTCCCAAATCTGGAGAGAAGATCTCCTGTGTGGTGGATCACGCCGGCTTAACTAAATCCATCATCGTAGACTGG-----------------------------------------------------------------------------------------------------------------------------------------------------------------------GATCCCGCTATGCCAGAGTCTGACAGGAATAAAATCGCCATCGGAGCGTCTGGTCTGGTGCTGGGGATCATCATAGCAGCTGCTGGACTCATTTACTACAAGAAGAAATCAACAGGGAGGATCCTGGTACCAAATC

>Rhsi-DAB1*08:04

CTGATGCTGTCGACTTTCACCGGAGCA-----------------------------------------------------------------------------------------------------------------------------------------------------------------------------------------------------------------------GCTCATGGATACTACTGGTCTATGTGGTCTAAATGCATCCACAGCCCCCGGGATTTCAGCGACATGGTGTTCATTGATAACTATATCTTCAATAAAGATGTGTTCATACAGTTCAACAGCACTGTGGGAGAGTATGTGGGGTACACTGAACTTGGAGTATATAATGCACGAAGAATGAACAACGATCCCAACCGTCTGCAGCAAGAGAGAGCTGAGGTGGAGAGATACTGCAAACATAATGCTGAAATCTATCAGGCAGCTATCGCT---GATAAAACA------------------------------------------------------------------------------------------------------------------------------------------------------------------------------------------------------------------------------------------------------------------------------------------------------------------------------------------------------------------------------------------------GTGGCACCAAAGGTCAAGCTCAGTTCAATGATGCAGGCCAGCGGCAGTCATCCTGCTGTACTGATGTGCAGCGCTTACCGCTTCTACCCACACTGGATCAAAGTGTCCTGGATGAGGGATGGTAAAGTTGTGAAGACTGACGTGACCTCAACTGAGGAGATGCCTAACGGAGACTGGTACTACCAGATTCACTCGCACCTGGAATACACTCCCAAATCTGGAGAGAAGATCTCCTGTGTGGTGGATCACGCCGGCTTAACTAAATCCATCATCGTAGACTGG-----------------------------------------------------------------------------------------------------------------------------------------------------------------------GATCCCGCTATGCCAGAGTCTGACAGGAATAAAATCGCCATCGGAGCGTCTGGTCTGGTGCTGGGGATCATCATAGCAGCTGCTGGACTCATTTACTACAAGAAGAAATCAACAGGGAGGATCCTGGTACCAAATC

>Rhsi-DAB1*08:05

CTGATGCTGTCTGCATTCACCGGAGCA-----------------------------------------------------------------------------------------------------------------------------------------------------------------------------------------------------------------------GCTCATGGATACTACTGGTCTATGTGGTCTAAATGCATCCACAGCTCCCGGGGTTTCAGCGACATGGTGTTCATTGATAACTATATCTTCAATAAAGATGTGTTCATACAGTTCAACAGCACTGTGGGAGAGTATGTGGGGTACACTGAACTTGGAGTATATAATGCACGAAGAATGAACAACGATCCCAACATTCTGCAGCAAGAGAGAGCTGAGGTGGAGAGATACTGCAAACATAATGCTGAAATCTATCAGGCAGCTATCGCT---GATAAAACA------------------------------------------------------------------------------------------------------------------------------------------------------------------------------------------------------------------------------------------------------------------------------------------------------------------------------------------------------------------------------------------------GTGGCACCAAAGGTCAAGCTCAGTTCAATGATGCAGGCCAGCGGCAGTCATCCTGCTGTACTGATGTGCAGCGCTTACCGCTTCTACCCACACTGGATCAAAGTGTCCTGGATGAGGGATGGTAAAGTTGTGAAGACTGACGTGACCTCAACTGAGGAGATGCCTAACGGAGACTGGTACTACCAGATTCACTCGCACCTGGAATACACTCCCAAATCTGGAGAGAAGATCTCCTGTGTGGTGGATCACGCCGGCTTAACTAAATCCATCATCGTAGACTGG-----------------------------------------------------------------------------------------------------------------------------------------------------------------------GATCCCGCTATGCCAGAGTCTGACAGGAATAAAATCGCCATCGGAGCGTCTGGTCTGGTGCTGGGGATCATCATAGCAGCTGCTGGACTCATTTACTACAAGAAGAAATCAACAGGGAGGATCCTGGTACCAAATC

>Rhsi-DAB1*08:06

CTGATGCTGTCGGCTTTCACTGGAGCA-----------------------------------------------------------------------------------------------------------------------------------------------------------------------------------------------------------------------GCTCATGGATACTACTGGTCTATGTGGTCTAAATGCATCCGCAGCTCCCGGGATTTCAGCGACATGGTGTTCATTGATAACTATATCTTCAATAAAGATGTGTTCATACAGTTCAACAGCACTGTGGGAGAGTATGTGGGGTACACTGAACTTGGAGTATATAATGCACGAAGAATGAACAACGATCCCAACATTCTGCAGCAAGAGAGAGCTGAGGTGGAGAGATACTGCAAACATAATGCTGAAATCTATCAGGCAGCTATCGCT---GATAAAACA------------------------------------------------------------------------------------------------------------------------------------------------------------------------------------------------------------------------------------------------------------------------------------------------------------------------------------------------------------------------------------------------GTGGCACCAAAGGTCAAGCTCAGTTCAATGATGCAGGCCAGCGGCAGTCATCCTGCTGTACTGATGTGCAGCGCTTACCGCTTCTACCCACACTGGATCAAAGTGTCCTGGATGAGGGATGGTAAAGTTGTGAAGACTGACGTGACCTCAACTGAGGAGATGCCTAACGGAGACTGGTACTACCAGATTCACTCGCACCTGGAATACACTCCCAAATCTGGAGAGAAGATCTCCTGTGTGGTGGATCACGCCGGCTTAACTAAATCCATCATCGTAGACTGG-----------------------------------------------------------------------------------------------------------------------------------------------------------------------GATCCCGCTATGCCAGAGTCTGACAGGAATAAAATCGCCATCGGAGCGTCTGGTCTGGTGCTGGGGATCAACATAGCAGCTGCTGGACTCATTTACTACAAGAAGAAATCAACAGGGAGGATCCTGGTACCAAATC

>Rhsi-DAB1*08:07

CTGATGCTGTCTGCTTTCACCGGAGCA-----------------------------------------------------------------------------------------------------------------------------------------------------------------------------------------------------------------------GCTCATGGATACTACTGGTCTATGTGGTCTAAATGCATCCACAGCTCCCGGGATTTCAGCGACATGGTGTTCATTGACAACTATATCTTCAATAAAGATGTGTTCATACAGTTCAACAGCACTGTGGGAGAGTATGTGGGGTACACTGAACTTGGAGTATATAATGCACGAAGAATGAACAACGATCCCAACATTCTGCAGCAAGAGAGAGCTGAGGTGGAGAGATACTGCAAACATAATGCTGAAATCTATCAGGCAGCTATCGCT---GATAAAACA------------------------------------------------------------------------------------------------------------------------------------------------------------------------------------------------------------------------------------------------------------------------------------------------------------------------------------------------------------------------------------------------GTGGCACCAAAGGTCAAGCTCAGTTCAATGATGCAGGCCAGCGGCAGTCATCCTGCTGTACTGATGTGCAGCGCTTACCGCTTCTACCCACACTGGATCAAAGTGTCCTGGATGAGGGATGGTAAAGTTGTGAAGACTGACGTGACCTCAACTGAGGAGATGCCTAACGGAGACTGGTACTACCAGATTCACTCGCACCTGGAATACACTCCCAAATCTGGAGAGAAGATCTCCTGTGTGGTGGATCACGCCGGCTTAACTAAATCCATCATCGTAGACTGG-----------------------------------------------------------------------------------------------------------------------------------------------------------------------GATCCCGCTATGCCAGAGTCTGACAGGAATAAAATCGCCATCGGAGCGTCTGGTCTGGTGCTGGGGATCATCATAGCAGCTGCTGGACTCATTTACTACAAGAAGAAATCAACAGGGAGGATCCTGGTACCAAATC

>Rhsi-DAB1*08:08

CTGATGCTGTCTGCATTTACTGGAGCA-----------------------------------------------------------------------------------------------------------------------------------------------------------------------------------------------------------------------GCTCATGGATACTACTGGTCTATGTGGTCTAAATGCATCCACAGCTCCCGGGATTTCAGCGACATGGTGTTCATTGGTAACTATATCTTCAATAAAGATGTGTTCATACAGTTCAACAGCACTGTGGGAGAGTATGTGGGGTACACTGAACTTGGAGTATATAATGCACGAAGAATGAACAACGATCCCAACCGTCTGCAGCAAGAGAGAGCTGAGGTGGAGAGATACTGCAAACATAATGCTGAAATCTATCAGGCAGCTATCGCT---GATAAAACA------------------------------------------------------------------------------------------------------------------------------------------------------------------------------------------------------------------------------------------------------------------------------------------------------------------------------------------------------------------------------------------------GTGGCACCAAAGGCCAAGCTCAGTTCAATGATGCAGGCCAGCGGCAGTCATCCTGCTGTACTGATGTGCAGCGCTTACCGCTTCTACCCACACTGGATCAAAGTGTCCTGGATGAGGGATGGTAAAGTTGTGAAGACTGACGTGACCTCAACTGAGGAGATGCCTAACGGAGACTGGTACTACCAGATTCACTCGCACCTGGAATACACTCCCAAATCTGGAGAGAAGATCTCCTGTGTGGTGGATCACGCCGGCTTAACTAAATCCATCATCGTAGACTGG-----------------------------------------------------------------------------------------------------------------------------------------------------------------------GATCCCGCTATGCCAGAGTCTGACAGGAATAAAATCGCCATCGGAGCGTCTGGTCTGGTGCTGGGGATCATCATAGCAGCTGCTGGACTCATTTACTACAAGAAGAAATCAACAGGGAGGATCCTGGTACCAAATC

>Rhsi-DAB1*09:01

CTGATGCTGTCTGCTTTTACTGGAGCA-----------------------------------------------------------------------------------------------------------------------------------------------------------------------------------------------------------------------GCTCATGGATACTACAATTCTGTGTGGTCTAAATGCATCCACAGCTCCCGGGATTTCAGCGACATGGTGTTGATTGATAACTATGTCTTCAATAAAGATGTGGTTGTACAGTTCAACAGCATTGTGGGAGAGTATGTGGGGTACACTGAACTTGGAGTAAGTAGTGCACGGAATTGGAACAGCGATCCCAACATTCTGCAGCAAGAGAGAGCTGAGGTGGAGAGATTCTGCAAACATAATGCTGAAATATATCAGGCAGCTATCGCT---GATAAAACA------------------------------------------------------------------------------------------------------------------------------------------------------------------------------------------------------------------------------------------------------------------------------------------------------------------------------------------------------------------------------------------------GTGGCACCAAAGGTCAAGCTCAGTTCAGTGACGCAGGCCGGCGGCAGACATCCTGCTGTACTGATGTGCAGCGCTTACCGCTTCTACCCGCACTGGATCAAAGTGTCCTGGATGAGAGACGGTACAGTTGTGAAGACTGACGTGACCTCAACTGAGGAGATGCCTAACGGAGACTGGTACTACCAGATTCACTCGCACCTGGAGTACACTCCCAAATCTGGAGAGAAGATCTCCTGTGTGGTGGATCACGCCGGCTTAACTAAATCCATCATCGTAGACTGG-----------------------------------------------------------------------------------------------------------------------------------------------------------------------GATCCCGCTATGCCAGAGTCTGACAGGAATAAAATCGCCATCGGGGCGTCTGGTCTGGTGCTGGGGATCATCATAGCAGCTGCTGGACTCATTTATTACAAGAAGAAATCAGCAGGGAGGATCCTGGTACCAAA

>Rhsi-DAB1*09:02

CTGATGCTGTCTACATTTACCGGAGCA-----------------------------------------------------------------------------------------------------------------------------------------------------------------------------------------------------------------------GCTCATGGATACTACAATTCTGTGTGGTCTAAATGCATCCACAGCTCCCGGGATTTCAGCGACATGGTGTTGATTGATAACTATGCCTTCAATAAAGATGTGGTTGTACAGTTCAACAGCACTGTGGGAGAGTATGTGGGGTACACTGAACTTGGAGTAAGTAGTGCACGGAATTGGAACAGCGATCCCAACATTCTGCAGCAAGAGAGAGCTGAGGTGGAGAGATTCTGCAAACATAATGCTGAAATATATCAGGCAGCTATCGCT---GATAAAACA------------------------------------------------------------------------------------------------------------------------------------------------------------------------------------------------------------------------------------------------------------------------------------------------------------------------------------------------------------------------------------------------GTGGCACCAAAGGTCAAGCTCAGTTCAGTGACGCAGGCCGGCGGCAGACATCCTGCTGTACTGATGTGCAGCGCTTACCGCTTCTACCCGCACTGGATCAAAGTGTCCTGGATGAGAGACGGTACAGTTGTGAAGACTGACGTGACCTCAACTGAGGAGATGCCTAACGGAGACTGGTACTACCAGATTCACTCGCACCTGGAGTACACTCCCAAATCTGGAGAGAAGATCTCCTGTGTGGTGGATCACGCCGGCTTAACTAAATCCATCATCGTAGACTGG-----------------------------------------------------------------------------------------------------------------------------------------------------------------------GATCCCGCTATGCCAGAGTCTGACAGGAATAAAATCGCCATCGGGGCGTCTGGTCTGGTGCTGGGGATCATCATAGCAGCTGCTGGACTCATTTATTACAAGGAGAAATCAACAGGGAGGATCCTGGTACCAAA

>Rhsi-DAB1*09:03

CTGATGCTGTCTACTTTCACCGGAGCA-----------------------------------------------------------------------------------------------------------------------------------------------------------------------------------------------------------------------GCTCATGGATACTACAATTCTGTGTGGTCTAAATGCACCCACAGCTCCCGGGATTTCAGCGACATGGTGTTGATTGATAACTATGTCTTCAATAAAGATGTGGTTGTACAGTTCAACAGCACTGTGGGAGAGTATGTGGGGTACACTGAACTTGGAGTAAGTAGTGCACGGAATTGGAACAGCGATCCCAACATTCTGCAGCAAGAGAGAGCTGAGGTGGAGAGATTCTGCAAACATAATGCTGAAATATATCAGGCAGCTATCGCT---GATAAAACA------------------------------------------------------------------------------------------------------------------------------------------------------------------------------------------------------------------------------------------------------------------------------------------------------------------------------------------------------------------------------------------------GTGGCACCAAAGGTCAAGCTCAGTTCAGTGACGCAGGCCGGCGGCAGACATCCTGCTGTACTGATGTGCAGCGCTTACCGCTTCTACCCGCACTGGATCAAAGTGTCCTGGATGAGAGACGGTACAGTTGTGAAGACTGGCGTGACCTCAACTGAGGAGATGCCTAACGGAGACTGGTACTACCAGATTCACTCGCACCTGGAGTACACTCCCAAATCTGGAGAGAAGATCTCCTGTGTGGTGGATCACGCCGGCTTAACTAAATCCATCATCGTAGACTGG-----------------------------------------------------------------------------------------------------------------------------------------------------------------------GATCCCGCTATGCCAGAGTCTGACAGGAATAAAATCGCCATCGGGGCGTCTGGTCTGGTGCTGGGGATCATCATAGCAGCTGCTGGACTCATTTATTACAAGAAGAAATCAACAGGGAGGATCCTGGTACCAAA

>Rhsi-DAB1*10:01

CTGATGCTGTCTGCTTTTACCGGATCA-----------------------------------------------------------------------------------------------------------------------------------------------------------------------------------------------------------------------GCTCATGGATACTACAGGTCTCGGTGGGCTAAATGCATCCACAGCTCCCGGGATTTCAGCGACATGGTGTACATTGATAACTATATCTTCAATAAAGATGTGTACATACAGTTCAACAGCACTGTGGGAGAGTTTGTGGGGTACACTGAACATGGAGTATATAATGCAAAATTACGGAACGACAATCCCAACATTCTGCAGGGAGAGAGAGCTGATGTGGAGAGATACTGCAAACATAATGCTAAAAACAGACAGGCAGCTATCGCA---GATGAAACA------------------------------------------------------------------------------------------------------------------------------------------------------------------------------------------------------------------------------------------------------------------------------------------------------------------------------------------------------------------------------------------------GTGGCACCAAAGGTCAAGCTCAGTTCAGTGACGCAGGCCGGCGGCAGACATCCTGCTGTACTGATGTGCAGCGCTTACCGCTTCTACCCACACTGGATCAAAGCGTCCTGGATGAGGGATGGTAAAGTTGTGAAGACTGACGTGACCTCAACTGAGGAGATGCCTAACGGAGACTGGTACTACCAGATTCACTCGCACCTGGAATACACTCCCAAATCTGGAGAGAAGATCTCCTGTGTGGTGGATCACGCCGGCTTAACTAAATCCATCATCGTAGACTGG-----------------------------------------------------------------------------------------------------------------------------------------------------------------------GATCCCGCTATGCCAGAGTCTGACAGGAATAAAATCGCCATCGGGGCGTCTGGTCTGGTGCTGGGGATCATCATAGCAGCTGCTGGACTCATTTACTACAAGAAGAAATCAACAGGGAGGATCCTGGTACCAAA

>Rhsi-DAB1*10:02

CTGATGCTGTCTGCATTCACTGGATCA-----------------------------------------------------------------------------------------------------------------------------------------------------------------------------------------------------------------------GCTCATGGATACTACAGGTCTCGGTGGGCTAAATGCATCCACAGCTCCCGGGATTTCAGCGACATGGCGTACATTGATAACTATATCTTCAATAAAGATGTGTACATACAGTTCAACAGCACTGTGGGAGAGTTTGTGGGGTACACTGAACATGGAGTATATAATGCAAAATTACGGAACGACAATCCCAACATTCTGCAGGGAGAGAGAGCTGATGTGGAGAGATACTGCAAACATAATGCTAAAAACAGACAGGCAGCTATCGCA---GATAAAACA------------------------------------------------------------------------------------------------------------------------------------------------------------------------------------------------------------------------------------------------------------------------------------------------------------------------------------------------------------------------------------------------GTGGCACCAAAGGTCAAGCTCAGTTCAGTGACGCAGGCCGGCGGCAGACATCCTGCTGTACTGATGTGCAGCGCTTACCGCTTCTACCCACACTGGATCAAAGTGTCCTGAATGAGGGATGGTAAAGTTGTGAAGACTGACGTGACCTCAACTGAGGAGATGCCTAACGGAGACTGGTACTACCAGATTCACTCGCACCTGGAATACACTCCCAAATCTGGAGAGAAGATCTCCTGTGTGGTGGATCACGCCGGCTTAACTAAATCCATCATCGTAGACTGG-----------------------------------------------------------------------------------------------------------------------------------------------------------------------GATCCCGCTATGCCAGAGTCTGACAGGAATAAAATCGCCATCGGGGCGTCTGGTCTGGTGCTGGGGATCATCATAGCAGCTGCTGGACTCATTTACTACAAGAAGAAATCAACAGGGAGGATCCTGGTACCAAA

>Rhsi-DAB1*10:03

CTGATGCTGTCTGCATTTACCGGATCA-----------------------------------------------------------------------------------------------------------------------------------------------------------------------------------------------------------------------GCTCATGGATACTACAGGTCTCGGTGGGCTAAATGCATCCACAGCTCCCGGGATTTCAGCGACATGGTGTACATTGATAACTATATTTTCAATAAAGATGTGTACATACAGTTCAACAGCACTGTGGGAGAGTTTGTGGGGTACACTGAACATGGAGTATATAATGCAAAATTACGGAACGACAATCCCAACATTCTGCAGGGAGAGAGAGCTGATGTGGAGAGATACTGCAAACATAATGCTAAAAACAGACAGGCAGCTATCGCA---GATAAAACA------------------------------------------------------------------------------------------------------------------------------------------------------------------------------------------------------------------------------------------------------------------------------------------------------------------------------------------------------------------------------------------------GTGGCACCAAAGGTCAAGCTCAGTTCAGTGACGCAGGCCGGCGGCAGACATCCTGCTGTACTGATGTGCAGCGCTTACCGCTTCTACCCACACTGGATCAAAGTGTCCTGGATGAGGGATGGTAAAGTTGTGAAGACTGACGTGACCTCAACTGAGGAGATGCCTAACGGAGACTGGTACTACCAGATTCACTCGCACCTAGAATACACTCCCAAATCTGGAGAGAAGATCTCCTGTGTGGTGGATCACGCCGGCTTAACTAAATCCATCATCGTAGACTGG-----------------------------------------------------------------------------------------------------------------------------------------------------------------------GATCCCGCTATGCCAGAGTCTGACAGGAATAAAATCGCCATCGGGGCGTCTGGTCTGGTGCTGGGGATCATCATAGCAGCTGCTGGACTCATTTACTACAAGAAGAAATCAACAGGGAGGATCCTGGTACCAAA

>Rhsi-DAB1*11:01

CTGATGCTGTCTGCATTTACTGGAGCA-----------------------------------------------------------------------------------------------------------------------------------------------------------------------------------------------------------------------GCTCATGGATACTACTTTTCTCGGTGGACGAAATGCATCTACAGCTCCCATGATTTAAGCGACATGGTGTTCATTGACAACTATTTCTTCAATAAAGATATGTTCATACACTTCAACAGCACTGTGGGAGTGTATGTGGGGTACACTGAATTTGGAGTATGTAATGCACGGAATTGGAACAACGATCATGACTTTCTGCAGGGAGAGAGAGCTCTGGTGGAGAGATACTGCAGACATAATGCTTTACTATATCAGGCAGCTGTCACT---GATAAAACA------------------------------------------------------------------------------------------------------------------------------------------------------------------------------------------------------------------------------------------------------------------------------------------------------------------------------------------------------------------------------------------------GTGGCACCAAAGGTCAAGCTCAGTTCAGTGACGCAGGCCGATGGCAGACATCCTGCTTTACTGATGTGCAGCGCTTACCGCTTCTACCCACACTGGATCAAAGTGTCCTGGATGAGAGACGGTACAGTTGTGAAGACTGATGTGACCTCAACTGAGGAGATGCCTAACGGAGACTGGTACTACCAGATTCACTCGCACCTGGAGTACACTCCCAAATCTGGAGAGAAGATCTCCTGTGTGGTGGATCACGCCGGCTTAACTAAATCCATCATCGCAGACTGG-----------------------------------------------------------------------------------------------------------------------------------------------------------------------GATCCCGCTATCCCTGAGTCTGACAAGAATAAAATCGCCATCGGGGCGTCTGGTCTGGTGCTGGGGATCATCATAGCAGCTGCTGGACTCATTTACTACAAGAAGAAATCAACAGGGAGGATCCTGGTACCAAATC

>Rhsi-DAB1*11:02

CTGATGCTGTCTGCTTTTACTGGAGCA-----------------------------------------------------------------------------------------------------------------------------------------------------------------------------------------------------------------------GCTCATGGATACTACTTTTCTCGGTGGACGAAATGCATCTACAGCTCCCATGATTTAAGCGACATGGTGTTCATTGATAACTATTTCTTCAATAAGGATATGTTCATACACTTCAACAGCACTGTGGGAGTGTATGTGGGGTACACTGAATTTGGAGTATGTAATGCACGGAATTGGAACAACGATCATGACTTTCTGCGGGGAGAGAGAGCTCTGGTGGAGAGATACTGCAGACATAATGCTTTACTATATCAGGCAGCTGTCACT---GATAAAACA------------------------------------------------------------------------------------------------------------------------------------------------------------------------------------------------------------------------------------------------------------------------------------------------------------------------------------------------------------------------------------------------GTGGCACCAAAGGTCAAGCTCAGTTCAGTGACGCAGGCCGATGGCAGACATCCTGCTTTACTGATGTGCAGCGCTTACCGCTTCTACCCACACTGGATCAAAGTGTCCTGGATGAGAGACGGTACAGTTGTGAAGACTGATGTGACCTCAACTGAGGAGATGCCTAACGGAGACTGGTACTACCAGATTCACTCGCACCTGGAATACACTCCCAAATCTGGAGAGAAGATCTCCTGTGTGGTGGATCACGCCGGCTTAACTAAATCCATCATCGTAGACTGGGGTTAGAGGAAACATTTATTTTCCCTCAGATAAGTAAATTAAATATTTTAACAAACAAAAATAAGACTGAATAAAATATGTAAATATACTGTAGTTACATTCACATTTGCCAAGCCCTTATTCAGCACTGTGCATATTAATATAAAACATGATTTCTGTACCCCACAGATCCCGCTATCCCTGAGTCTGACAAGAATAAAATCGCCATCGGGGCGTCTGGTCTGGTGCTGGGGATCATCATAGCAGCTGCTGGACTCATTTATTACAAGAAGAAATCAACAGGGAGGATCCTGGTACCAAATC

>Rhsi-DAB1*12:01

CTGATGCTGTCTGCTTTCACTGGAGCA---------------------------GGTAAATACAATCAAGAGTAAAGCAAAATCGATAGATATATAATGAGAGTAATGTTATTCTTCAATAGTGGTAGAAAAGACGTGCGTTAATACAACAACCAACTAATGTATAAAAGTCCTTCTGTTTTTCTGTAGTTGACATGAAATCATCTGATTTAACTGTAATCCTGTTTTAACATTATTTTTCAGCTCATGGATACTACAATTCTCGGTGGGCTGAATGCATCCACAGCTCCCGGGATTTCAGTGACATGGTGTTCATTGATAACTATGTCTTCAATAAAGATGTGTTCATACAGTTCAACAGCACTGTGGGAGAGTTTGTGGGGTACACTGAACTTGGAGTAAGTAATGCACGGAATTGGAACAGCGATCCCAACCGTCTGCAGCAAGAGAGAGCTCTGGTGGAGACATACTGCAAACATAATGCTGAACTCTATCAGGCAGCTATCGCA---GATAAAACA---GGTACAGGCTTCTGATCCTCCTGTCACACGGGAAACTCTTGTTTTACACATTTACAATAAGTGCTTTCACACAACAGTTTGCTAACTGTATAATTATTTTAAAAGTGTGTTATGAGAACCATTACAGTCTTCAGTTTCGTAATAAACATTTTAAAATGCTTGTTTTCACCGTTGGACGCGCACGAGCACAGCGGCGAGATTAGAACCTT

>Rhsi-DAB1*13:01

CTGATGCTGTCGACTTTTACTGGACCA-----------------------------------------------------------------------------------------------------------------------------------------------------------------------------------------------------------------------GCTCATGGATACTACTATTCTCGGTGGAATAAATGCATCCACAGCTCCCGGGATTTCAGCGACATGGTGTTCATTGATAACTATATCTTCAATAAAGTTGTGCACATACAGTTCAACAGCACTGTGGGAGAGTTTGTGGGGTACACTGAACATGGAGTATATAATGCACGGAATTGGAACAACGATCCCAACATTCTGCAGTCAGAGAGAGCTGATGTGGAGAGAGTCTGCAAACATAATGCTGAAATGTATCAGGCAGCTATCGCT---GATAAAACA------------------------------------------------------------------------------------------------------------------------------------------------------------------------------------------------------------------------------------------------------------------------------------------------------------------------------------------------------------------------------------------------GTGGCACCAAAGGTCAAGCTCAGTTCAATGATGCAGGCCAGCGGCAGTCATCCTGCTGTACTGATGTGCAGCGCTTACCGCTTCTACCCACACTGGATCAAAGTGTCCTGGATGAGGGATGGTAAAGTTGTGAAGACTGACGTGACCTCAACTGAGGAGATGCCTAACGGAGACTGGTACTACCAGATTCACTCGCACCTGGAATACACTCCCAAATCTGGAGAGAAGATCTCCTGTGTGGTGGATCACGCCGGCTTAACTAAATCCATCATCGTAGACTGG-----------------------------------------------------------------------------------------------------------------------------------------------------------------------GATCCCGCTATGCCAGAGTCTGACAGGAATAAAATCGCCATCGGAGCGTCTGGTCTGGTGCTGGGGATCATCATAGCAGCTGCTGGACTCATTTACTACAAGAAGAAATCAACAGGGAGGATCCTGGTACCAAATC

>Rhsi-DAB1*14:01

CTGATGCTGTCTGCATTCACTGGAGCA-----------------------------------------------------------------------------------------------------------------------------------------------------------------------------------------------------------------------GCTCATGGATACTACAATTCTGTGTGGTCTAAATGCATCCACAGCTCCCGGGATTTCAGCGACATGGTGTTGATTGATAACTATGTCTTCAATAAAGATGTGGTTGTACAGTTCAACAGCACTGTGGGAGAGTATGTGGGGTACACTGAACTTGGAGTAAGTAGTGCACGGAATTGGAACAGCGATCCCAACATTCTGCAGCAAGAGAGAGCTGAGGTGGAGAGATTCTGCAAACATAATGCTGAAATATATCAGGCAGCTATCGCT---GATAGAACA------------------------------------------------------------------------------------------------------------------------------------------------------------------------------------------------------------------------------------------------------------------------------------------------------------------------------------------------------------------------------------------------GTGGCACCAAAGGTCAAGCTCAGTTCAGTGACGCAGGCCGGCGGCAGACATCCTGCTGTACTGATGTGCAGCGCTTACCGCTTCTACCCGCACTGGATCAAAGTGTCCTGGATGAGAGACGGTACAGTTGTGAAGACTGACGTGACCTCAACTGAGGAGATGCCTAACGGAGACTGGTACTACCAGATTCACTCGCACCTGGAGTACACTCCCAAATCTGGAGAGAAGATCTCCTGTGTGGTGGATCACGCCGGCTTAACTAAATCCATCATCGTAGACTGG-----------------------------------------------------------------------------------------------------------------------------------------------------------------------GATCCCGCTATGCCAGAGTCTGACAGGAATAAAATCGCCATCGGGGCGTCTGGTCTGGTGCTGGGGATCATCATAGCAGCTGCTGGACTCATTTATTACAAGAAGAAATCAACAGGGAGGATCCTGGTACCAAA

>Rhsi-DAB1*15:01

CTGATGCTGTCTGCTTTTACTGGATCA-----------------------------------------------------------------------------------------------------------------------------------------------------------------------------------------------------------------------GCTCATGGATACTACTTTTCTCGGTGGGCTAAATGCATCCACAGCTCCCGGGATTTCAGCGACATGGTGTTCATTGATAACTATGTCTTCAATAAAGATGTGTTCATACAGTTCAACAGCTCTGTGGGAGAGTTTGTGGGGTACACTGAACTTGGAGTATATAATGCACGGAATTGGAACAACGATCCCAACCATCTGCAGTCAGAGAGAGCTCTGGTGGAGACATTCTGCAAACATAATGCTGAAATCTATCAGGCAGCTATCGCT---GATAAAACA------------------------------------------------------------------------------------------------------------------------------------------------------------------------------------------------------------------------------------------------------------------------------------------------------------------------------------------------------------------------------------------------GTGGCACCAAAGGTCAAGCTCAGTTCAGTGATGCAGGCCAGCGGCAGTCATCCTGCTGTACTGATGTGCAGCGCTTACCGCTTCTACCCACACTGGATCAAAGTGTCCTGGATGAGGGATGGTAAAGTTGTGAAGACTGACGTGACCTCAACTGAGGAGATGCCTAACGGAGACTGGTGCTACCAGATTCACTCGCACCTGGAATACACTCCCAAATCTGGAGAGAAGATCTCCTGTGTGGTGGATCACGCCGGCTTAACTAAATCCATCATCGTAGACTGG-----------------------------------------------------------------------------------------------------------------------------------------------------------------------GATCCTGCTATGCCAGAGTCTGACAGGAATAAAATCGCCATTGGGGCGTCTGGTCTGGTGCTGGGGATCATCATAGCAGCTGCTGGACTCATTTACTACAAGAAGAAATCAACAGGGAGGATCCTGGTACCAAA

>Rhsi-DAB3*01:01:01

CTGATGCTGTCTACTTTCACTGGAACA-----------------------------------------------------------------------------------------------------------------------------------------------------------------------------------------------------------------------GCTGATGGATATTATCAATACGACATAGCTGAATGCTTCTACAGCACCAGTGATTACAGCGATATGGTGTATCTACGTTCATTTTCATTCAATAAAGTTGTGGATGTACAGTTCAACAGCACTTTGGGGAAGTGTGTGGGCTACACTGAACAAGGAGTGAAACATGCAGAGAACTTCAACAATGACCCGGCCGTCTTGCAGCAGATGAAAGCTGAGGTGGACACATTCTGCAGACATAATGCTCAGATCTGGGACACAGCTGTTCGT---GATAAAGCA------------------------------------------------------------------------------------------------------------------------------------------------------------------------------------------------------------------------------------------------------------------------------------------------------------------------------------------------------------------------------------------------GTGAAACCGACGGTTATGATCAGTTCAGTAAAACGGGCTGATGGCAGTCATCCAGCTGTTCTGATGTGCAGCGCATATGAATTCTACCCCTCAAAAATCAAAGTGTCTTGGCTGAGAGATGGTAAACCGATGACCTCTGATGTGACCTCCACAATGGAGATGGCTGATGGGGACTGGTACTACCAGATTCACTCTGAGCTGGAATACACTCCTAAATCTGGGGAGAAGATCTCCTGTATGGTGGAGCACGCCAGCTTCAATAAACCCATGATTTATGACTGG-----------------------------------------------------------------------------------------------------------------------------------------------------------------------GATCCCGCTATCTCTGAGTCTGACAGGAATAAAATCGCCATCGGGGCGTCTGGTCTGGTGCTGGGGATCATCATAGCAGCTGCTGGACTCATTTACTACAAGAAGAAATCAACAGGGAGGATCCTGGTACCAAATC

>Rhsi-DAB3*01:01:02

CTGATGCTGTCTGCTTTTACTGGAACA-----------------------------------------------------------------------------------------------------------------------------------------------------------------------------------------------------------------------GCTGATGGATATTATCAATACGACATAGCTGAATGCTTCTACAGCACCAGTGATTACAGCGATATGGTGTATCTACGTTCATTCTCATTCAATAAAGTTGTGGATGTACAGTTCAACAGCACTTTGGGGAAGTGTGTGGGCTACACTGAACAAGGAGTGAAACATGCAGAGAACTTCAACAATGACCCGGCCGTCTTGCAGCAGATGAAAGCTGAGGTGGACACATTCTGCAGACATAATGCTCAGATCTGGGACACAGCTGTTCGT---GATAAAGCA------------------------------------------------------------------------------------------------------------------------------------------------------------------------------------------------------------------------------------------------------------------------------------------------------------------------------------------------------------------------------------------------GTGAAACCGACGGTTATGATCAGTTCAGTAAAACAGGCTGATAGCAGTCATCCAGCTGTTCTGATGTGCAGCGCATATGAATTCTACCCCTCAAAAATCAAAGTGTCTTGGCTGAGAGATGGTAAACCGATGACCTCTGATGTGACCTCCACAATGGAGATGGCTGATGGGGACTGGTACTACCAGATTCACTCTGAGCTGGAGTACACTCCTAAATCTGGGGAGAAGATCTCCTGTATGGTGGAGCACGCCAGCTTCAATAAACCCATGATTTATGACTGG-----------------------------------------------------------------------------------------------------------------------------------------------------------------------GATCCCGCTATCTCTGAGTCTGACAGGAATAAAATCGCCATCGGGGCGTCTGGTCTGGTGCTGGGGATCATCATAGCAGCTGCTGGACTCATTTACTACAAGAAGAAATCAACAGGGAGGATCCTGGTACCAAA

>Rhsi-DAB3*01:01:03

CTGATGCTGTCGGCTTTTACTGGAACA-----------------------------------------------------------------------------------------------------------------------------------------------------------------------------------------------------------------------GCTGATGGATATTATCAATACGACATAGCTGAATGCTTCTACAGCACCAGTGATTACAGCGATATGGTGTATCTACGTTCATTTTCATTCAATAAAGTTGTGGATGTACAGTTCAACAGCACTTTGGGGAAGTGTGTGGGCTACACTGAACAAGGAGTGAAACATGCAGAGAACTTCAACAATGACCCGGCCGTCTTGCAGCAGATGAAAGCTGAGGTGGACACATTCTGCAGACATAATGCTCAGATCTGGGACACAGCTGTTCGC---GATAAAGCA------------------------------------------------------------------------------------------------------------------------------------------------------------------------------------------------------------------------------------------------------------------------------------------------------------------------------------------------------------------------------------------------GTGAAACCGACGGTTATGATCAGTTCAGTAAAACAGGCTGATGGCAGTCATCCAGCTGTTCTGATGTGCAGCGCATATGAATTCTACCCCTCAAAAATCAAAGTGTCTTGGCTGAGAGATGGTAAACCGATGACCTCTGATGTGACCTCCACAATGGAGATGGCTGATGGGGACTGGTACTACCAGATTCACTCTGAGCTGGAATACACTCCTAAATCTGGGGAGAAGATCTCCTGTATGGTGGAGCACGCCAGCTTCAGTAAACCCATGATTTATGACTGG-----------------------------------------------------------------------------------------------------------------------------------------------------------------------GATCCCGCTATCTCTGAGTCTGACAGGAATAAAATCGCCATCGGGGCGTCTGGTCTGGTGCTGGGGATCATCATAGCAGCTGCTGGACTCATTTACTACAAGAAGAAATCAACAGGGAGGATCCTGGTACCAAATC

>Rhsi-DAB3*01:01:04

CTGATGCTGTCTGCATTCACCGGAACA-----------------------------------------------------------------------------------------------------------------------------------------------------------------------------------------------------------------------GCTGATGGATACTATCAATACGACATAGCTGAATGCTTCTACAGCACCAGTGATTACAGCGATATGGTGTATCTACGTTCATTTTCATTCAATAAAGTTGTGGATGTACAGTTCAACAGCACTTTGGGGAAGTGTGTGGGCTACACTGAACAAGGAGTGAAACATGCAGAGAACTTCAACAATGACCCGGCCGTCTTGCAGCAGATGAAAGCTGAGGTGGACACATTCTGCAGACATAATGCTCAGATCTGGGACACAGCTGTTCGT---GATAAAGCA------------------------------------------------------------------------------------------------------------------------------------------------------------------------------------------------------------------------------------------------------------------------------------------------------------------------------------------------------------------------------------------------GTGAAACCGACGGTTATGATCAGTTCAGTAAAACAGGCTGATGGCAGTCATCCAGCTGTTCTGATGTGCAGCGCATATGAATTCTACCCCTCAAAAATCAAAGTGTCTTGGCTGAGAGATGGTAAACCGATGACCTCTGATGTGACCTCCACAATGGAGATGGCTGATGGGGACTGGTACTACCAGATTCACTCTGAGCTGGAATGCACTCCTAAATCTGGGGAGAAGATCTCCTGTATGGTGGAGCACGCCAGCTTCAATAAACCCATGATTTATGACTGG-----------------------------------------------------------------------------------------------------------------------------------------------------------------------GATCCCGCTATCTCTGAGTCTGACAGGAATAAAATCGCCATCGGGGCGTCTGGTCTGGTGCTGGGGATCATCATAGCAGCTGCTGGACTCATTTACTACAAGAAGAAATCAACAGGGAGGATCCTGGTACCAAATC

>Rhsi-DAB3*01:01:05

CTGATGCTGTCGACTTTTACCGGAACA-----------------------------------------------------------------------------------------------------------------------------------------------------------------------------------------------------------------------GCTGATGGATATTATCAATACGACATAGCTGAATGCTTCTACAGCACCAGTGATTACAGCGATATGGTGTATCTACGTTCATTTTCATTCAACAAAGTTGTGGATGTACAGTTCAACAGCACTTTGGGGAAGTGTGTGGGCTACACTGAACAAGGAGTGAAACATGCAGAGAACTTCAACAATGACCCGGCCGTCTTGCAGCAGATGAAAGCTGAGGTGGACACATTCTGCAGACATAATGCTCAGATCTGGGACACAGCTGTTCGT---GATAAAGCA------------------------------------------------------------------------------------------------------------------------------------------------------------------------------------------------------------------------------------------------------------------------------------------------------------------------------------------------------------------------------------------------GTGAAACCGACGGTTATGATCAGTTCAGTAAAACAGGCTGATGGCAGTCATCCAGCTGTTCTGATGTGCAGCGCATATGAATTCTACCCCTCAAAAATCAAAGTGTCTTGGCTGAGAGATGGTAAACCGATGACCTCTGATGTGACCTCCACAATGGAGATGGCTGATGGGGACTGGTACTACCAGATTCACTCTGAGCTGGAATACACTCCTAAATCTGGGGAGAAGATCTCCTGTATGGTGGAGCACGCCAGCTTCAATAAACCCATGATTTATGACTGG-----------------------------------------------------------------------------------------------------------------------------------------------------------------------GATCCCGCTATCTCTGAGTCTGACAGGAATAAAATCGCCATCGGGGCGTCTGGTCTGGTGCTGGGGATCATCATAGCAGCTGCTGGACTCATTTACTACAAGAAGAAATCAACAGGGAGGATCCTGGTACCAAATC

>Rhsi-DAB3*01:02

CTGATGCTGTCGGCTTTTACTGGAACA-----------------------------------------------------------------------------------------------------------------------------------------------------------------------------------------------------------------------GCTGATGGATATTATCAATACGACATAGCTGAATGCTTCTACAGCACCAGTGATTACAGCGATATGGTGTATCTACGTTCATTTTCATTCAATAAAGTTGTGGATGTACAGTTCAACAGCACTGTGGGGAAGTATGTGGGCTACACTGAACAAGGAGTGAAACATGCAGAGAACTTCAACAATGACCCGGCCGTCTTGCAGCAGATGAAAGCTGAGGTGGACAGATTCTGCAGACATAATGCTCAGATCTTGGACACAGCTGTTCGT---GATAAAGCA------------------------------------------------------------------------------------------------------------------------------------------------------------------------------------------------------------------------------------------------------------------------------------------------------------------------------------------------------------------------------------------------GTGAAACCGACGGTTATGATCAGTTCAGTAAAACAGGCTGATGGCAGTCATCCAGCTGTTCTGATGTGCAGCGCATATGAATTCTACCCCTCAAAAATCAAAGTGTCTTGGCTGAGAGATGGTAAACCGATGACCTCTGATGTGACCTCCACAATGGAGATGGCTGATGGGGACTGGTACTACCAGATTCACTCTGAGCTGGAATACACTCCTAAATCTGGGGAGAAGATCTCCTGTATGGTGGAGCACGCCAGCTTCAATAAACCCATGATTTATGACTGG-----------------------------------------------------------------------------------------------------------------------------------------------------------------------GATCCCGCTATCTCTGAGTCTGACAGGAATAAAATCGCCATCGGGGCGTCTGGTCTGGTGCTGGGGATCATCATAGCAGCTGCTGGACTCATTTACTACAAGAAGAAATCAACAGGGAGGATCCTGGTACCAAA

>Rhsi-DAB3*01:03

CTGATGCTGTCTGCATTTACCGGAACA-----------------------------------------------------------------------------------------------------------------------------------------------------------------------------------------------------------------------GCTGATGGATATTATCAATACGACATAGCTGAATGCTTCTACAGCACCAGTGATTACAGCGATATGGTGTATCTACGTTCATTTTCATTCAATAAAGTTGTGGATGTACAGTTCAACAGCACTTTGGGGAAGTGTGTGGGCTACACTGAACAAGGAGTGAAACATGCAGAGAACTTCAACAATGACCCGGCCGTCTTGCAGCAGATGAAAGTTGAGGTGGACACATTCTGCAGACATAATGCTCAGATCTGGGACACAGCTGTTCGT---GATAAGGCA------------------------------------------------------------------------------------------------------------------------------------------------------------------------------------------------------------------------------------------------------------------------------------------------------------------------------------------------------------------------------------------------GTGAAACCGACGGTTATGATCAGTTCAGTAAAACAGGCTGATGGCAGTCATCCAGCTGTTCTGATGTGCAGCGCATATGAATTCTACCCCTCAAAAATCAAAGTGTCATGGCTGAGAGATGGTAAACCGATGACCTCTGATGTGACCTCCACAATGGAGATGGCTGATGGGGACTGGTACTACCAGATTCACTCTGAGCTGGAATACACTCCTAAATCTGGGGAGAAGATCTCCTGTATGGTGGAGCACGCCAGCTTCAATAAACCCATGATTTATGACTGG-----------------------------------------------------------------------------------------------------------------------------------------------------------------------GATCCCGCTATCTCTGAGTCTGACAGGAATAAAATCGCCATCGGGGCGTCTGGTCTGGTGCTGGGGATCATCATAGCAGCTGCTGGACTCATTTACTACAAGAAGAAATCAACAGGGAGGATCCTGGTACCAAATC

>Rhsi-DAB3*01:04

CTGATGCTGTCTGCATTCACTGGAACA-----------------------------------------------------------------------------------------------------------------------------------------------------------------------------------------------------------------------GCTGATGGATATTATCAATACGACATAGCTGAATGCTTCTACAGCACCAGTGATTACAGCGATATGGTGTATCTACGTTCATTTTCATTCAGTAAAGTTGTGGATGTACAGTTCAACAGCACTTTGGGGAAGTGTGTGGGCTACACTGAACAAGGAGTGAAACATGCAGAGAACTTCAACAATGACCCGGCCGTCTTGCAGCAGATGAAAGCTGAGGTGGACACATTCTGCAGACATAATGCTCAGATCTGGGACACAGCTGTTCGT---GATAAAGCA------------------------------------------------------------------------------------------------------------------------------------------------------------------------------------------------------------------------------------------------------------------------------------------------------------------------------------------------------------------------------------------------GTGAAACCGACGGTTATGATCAGTTCAGTAAAACAGGCTGATGGCAGTCATCCAGCTGTTCTGATGTGCAGCGCATATGAATCCTACCCCTCAAAAATCAAAGTGTCTTGGCTGAGAGATGGTAAACCGATGACCTCTGATGTGACCTCCACAATGGAGATGGCTGATGGGGACTGGTACTACCAGATTCACTCTGAGCTGGAATACACTCCTAAATCTGGGGAGAAGATCTCCTGTATGGTGGAGCACGCCAGCTTCAATAAACCCATGATTTATGACTGG-----------------------------------------------------------------------------------------------------------------------------------------------------------------------GATCCCGCTATCTCTGAGTCTGACAGGAATAAAATCGCCATCGGGGCGTCTGGTCTGGTGCTGGGGATCATCATAGCAGCTGCTGGACTCATTTACTACAAGAAGAAATCAACAGGGAGGATCCTGGTACCAAATC

>Rhsi-DAB3*01:05

CTGATGCTGTCTGCATTTACTGGAACA-----------------------------------------------------------------------------------------------------------------------------------------------------------------------------------------------------------------------GCTGATGGATATCATCAATACGACATAGCTGAATGCTTCTACAGCACCAGTGATTACAGCGATATGGTGTATCTACGTTCATTTTCATTCAATAAAGTTGTGGATGTACAGTTCAACAGCACTGTGGGGAAGTATGTGGGCTACACTGAACAAGGAGTGAAACATGCAGAGAACTTCAACAATGACCCGGCCGTCTTGCAGCAGATGAAAGCTGAGGTGGACAGATTCTGCAGACATAATGCTCAGATCTTGGACACAGCTGTTCGG---GATAAAGCA------------------------------------------------------------------------------------------------------------------------------------------------------------------------------------------------------------------------------------------------------------------------------------------------------------------------------------------------------------------------------------------------GTGAAACCGACGGTTATGATCAGTTCAGTAAAACAGGCTGATGGCAGTCATCCAGCTGTTCTGATGTGCAGCGCATATGAATTCTACCCCTCAAAAATCAAAGTGTCTTGGCTGAGAGATGGTAAACCGATGACCTCTGATGTGACCTCCACAATGGAGATGGCTGATGGGGACTGGTACTACCAGATTCACTCTGAGCTGGAATACACTCCTAAATCTGGGGAGAAGATCTCCTGTATGGTGGAGCACGCCAGCTTCAATAAACCCATGATTTATGACTGG-----------------------------------------------------------------------------------------------------------------------------------------------------------------------GATCCCGCTATCTCTGAGTCTGACAGGAATAAAATCGCCATCGGGGCGTCTGGTCTGGTGCTGGGGATCATCATAGCAGCTGCTGGACTCATTTACTACAAGAAGAAATCAACAGGGAGGATCCTGGTACCAAA

>Rhsi-DAB3*01:06

CTGATGCTGTCGACATTCACTGGAACA-----------------------------------------------------------------------------------------------------------------------------------------------------------------------------------------------------------------------GCTGATGGATATTATCAATACGACATAGCTGAATGCTTCTACAGCACCAGTGATTACAGCGATATGGTGTATCTACGTTCATTTTCATTCAATAAAGTTGTGGATGTACAGTTCAACCGCACTGTGGGGAAGTATGTGGGCTACACTGAACAAGGAGTGAAACATGCAGAGAACTTCAACAATGACCCGGCCGTCTTGCAGCAGATGAAAGCTGAGGTGGACAGATTCTGCAGACATAATGCTCAGATCTTGGACACAGCTGTTCGT---GATAAAGCA------------------------------------------------------------------------------------------------------------------------------------------------------------------------------------------------------------------------------------------------------------------------------------------------------------------------------------------------------------------------------------------------GTGAAACCGACGGTTATGATCAGTTCAGTAAAACAGGCTGATGGCAGTCATCCAGCTGTTCTGATGTGCAGCGCATATGAATTCTACCCCTCAAAAATCAAAGTGTCTTGGCTGAGAGATGGTAAACCGATGACCTCTGATGTGACCTCCACAATGGAGATGGCTGATGGGGACTGGTACTACCAGATTCACTCTGAGCTGGAATACACTCCTAAATCTGGGGAGAAGATCTCCTGTATGGTGGAGCACGCCAGCTTCAATAAACCCATGATTTATGACTGG-----------------------------------------------------------------------------------------------------------------------------------------------------------------------GATCCCGCTATCTCTGAGTCTGACAGGAATAAAATCGCCATCGGGGCGTCTGGTCTGGTGCTGGGGATCATCATAGCAGCTGCTGGACTCATTTACTACAAGAAGAAATCAACAGGGAGGATCCTGGTACCAAA

>Rhsi-DAB3*01:07

CTGATGCTGTCTGCTTTTACTGGAACA-----------------------------------------------------------------------------------------------------------------------------------------------------------------------------------------------------------------------GCTGATGGATATTATCAATACGACATAGCTGAATGCTTCTACAGCACCAGTGATTACAGCGATATGGTGTATCTACGTTCATTTTCATTCAATAAAGTTGTGGATGTACAGTTCAACAGCACTTTGGGGAAGTGTGTGGGCTACACTGAACAAGGAGTGAAACATGCAGAGAACTTCAACGATGACCCGGCCGTCTTGCAGCAGATGAAAGCTGAGGTGGACACATTCTGCAGACATAATGCCCAGATCTGGGACACAGCTGTTCGT---GATAAAGCA------------------------------------------------------------------------------------------------------------------------------------------------------------------------------------------------------------------------------------------------------------------------------------------------------------------------------------------------------------------------------------------------GTGAAACCGACGGTTATGATCAGTTCAGTAAAACAGGCTGATGGCAGTCATCCAGCTGTTCTGATGTGCAGCGCATATGAATTCTACCCCTCAAAAATCAAAGTGTCTTGGCTGAGAGATGGTAAACCGATGACCTCTGATGTGACCTCCACAATGGAGATGGCTGATGGGGACTGGTACTACCAGATTCACTCTGAGCTGGAATACACTCCTAAATCTGGGGAGAAGATCTCCTGTATGGTGGAGCACGCCAGCTTCAATAAACCCATGATTTATGACTGG-----------------------------------------------------------------------------------------------------------------------------------------------------------------------GATCCCGCTATCTCTGAGTCTGACAGGAATAAAATCGCCATCGGGGCGTCTGGTCTGGTGCTGGGGATCATCATAGCAGCTGCTGGACTCATTTACTACAAGAAGAAATCAACAGGGAGGATCCTGGTACCAAATC

>Rhsi-DAB3*01:08

CTGATGCTGTCTGCTTTTACCGGAACA-----------------------------------------------------------------------------------------------------------------------------------------------------------------------------------------------------------------------GCTGATGGATATTATCAATACGACATAGCTGAATGCTTCTACAGCACCAGTGATTACAGCGATATGGTGTATCTACGTTCATTTTCATTCAATAATGTTGTGGATGTACAGTTCAACAGCACTTTGGGGAAGTGTGTGGGCTACACTGAACAAGGAGTGAAACATGCAGAGAACTTCAACAATGACCCGGCCGTCTTGCAGCAGATGAAAGCTGAGGTGGACACATTCTGCAGACATAATGCTCAGATCTGGGACACAGCTGTTCGT---GATAAAGCA------------------------------------------------------------------------------------------------------------------------------------------------------------------------------------------------------------------------------------------------------------------------------------------------------------------------------------------------------------------------------------------------GTGAAACCGACGGTTATGATCAGTTCAGTAAAACAGGCTGATGGCAGTCATCCAGCTGTTCTGATGTGCAGCGCATATGAATTCTACCCCTCAAAAATCAAAGTGTCTTGGCTGAGAGATGGTAAACCGATGACCTCTGATGTGACCTCCACAATGGAGATGGCTGATGGGGACTGGTACTACCAGATTCACTCTGAGCTGGAATACACTCCTAAATCTGGGGAGAAGATCTCCTGTATGGTGGAGCACGCCAGCTTCAATAAACCCATGATTTATGACTTG-----------------------------------------------------------------------------------------------------------------------------------------------------------------------GATCCCGCTATCTCTGAGTCTGACAGGAATAAAATCGCCATCGGGGCGTCTGGTCTGGTGCTGGGGATCATCATAGCAGCTGCTGGACTCATTTACTACAAGAAGAAATCAACAGGGAGGATCCTGGTACCAAA

>Rhsi-DAB3*01:09

CTGATGCTGTCGGCATTTACCGGAACA-----------------------------------------------------------------------------------------------------------------------------------------------------------------------------------------------------------------------GCTGATGGATATTATCAATACGACATAGCTGAATGCTTCTACAGCACCTGTGATTACAGCGATATGGTGTATCTACGTTCATTTTCATTCAATAAAGTTGTGGATGTACAGTTCAACAGCACTTTGGGGAAGTGTGTGGGCAACACTGAACAAGGAGTGAAACATGCAGAGAACTTCAACAATGACCCGGCCGTCTTGCAGCAGATGAAAGCTGAGGTGGACACATTCTGCAGACATAATGCTCAGATCTGGGACACAGCTGTTCGT---GATAAAGCA------------------------------------------------------------------------------------------------------------------------------------------------------------------------------------------------------------------------------------------------------------------------------------------------------------------------------------------------------------------------------------------------GTGAAACCGACGGTTATGATCAGTTCAGTAAAACAGGCTGATGGCAGTCATCCAGCTGTTCTGATGTGCAGCGCATATGAATTCTACCCCTCAAAAATCAAAGTGTCTTGGCTGAGAGATGGTAAACCGATGACCTCTGATGTGACCTCCACAATGGAGATGGCTGATGGGGACTGGTACTACCAGATTCACTCTGAACTGGAATACACTCCTAAATCTGGGGAGAAGATCTCCTGTATGGTGGAGCACGCCAGCTTCAATAAACCCATGATTTATGACTGG-----------------------------------------------------------------------------------------------------------------------------------------------------------------------GATCCCGCTATCTCTGAGTCTGACAGGAATAAAATCGCCATCGGGGCGTCTGGTCTGGTGCTGGGGATCATCATAGCAGCTGCTGGACTCATTTACTACAAGAAGAAATCAACAGGGAGGATCCTGGTACCAAA

>Rhsi-DAB3*01:10

CTGATGCTGTCTACTTTCACTGGAACA-----------------------------------------------------------------------------------------------------------------------------------------------------------------------------------------------------------------------GCAGATGGATATTATCAATACGACATAGCTGAATGCTTCTACAGCACCAGTGATTACAGCGATATGGTGTATCTACGTTCATTTTCATTCAATAAAGTTGTGGATGTACAGTTCAACAGCACTTTGGGGAAGTGTGTGGGCTACACTGAACAAGGAGTGAAACATGCAGAGAACTTCAACAATGACCCGGCCGTCTTGCAGCAGATGAAAGCTGAGGTGGACACATTCTGCAGACATAAGGCTCAGATCTGGGACACAGCTGTTCGT---GATAAAGCA------------------------------------------------------------------------------------------------------------------------------------------------------------------------------------------------------------------------------------------------------------------------------------------------------------------------------------------------------------------------------------------------GTGAAACCGACGGTTATGATCAGTTCAGTAAAACAGGCTGATGGCAGTCATCCAGCTGTTCTGATGTGCAGCGCATATGAATTCTACCCCTCAAAAATCAAAGTGTCTTGGCTGAGAGATGGTAAACCGATGACCTCTGATGTGACCTCCACAATGGAGATGGCTGATGGGGACTGGTACTACCAGATTCACTCTGAGCTGGAATACACTCCTAAATCTGGGGAGAAGATCTCCTGTATGGTGGAGCACGCCAGCTTCAATAAACCCATGATTTATGACTGG-----------------------------------------------------------------------------------------------------------------------------------------------------------------------GATCCCGCTATCTCTGAGTCTGACAGGAATAAAATCGCCATCGGGGCGTCTGGTCTGGTGCTGGGGATCATCATAGCAGCTGCTGGACTCATTTACTACAAGAAGAAATCAACAGGGAGGATCCTGGTACCAAA

>Rhsi-DAB3*01:11

CTGATGCTGTCTACTTTTACCGGAACA-----------------------------------------------------------------------------------------------------------------------------------------------------------------------------------------------------------------------GCTGATGGATATTATCAATACGACATAGCTGAATGCTTCTACAGCACCAGTGATTACAGCGATATGGTGTATCTACGTTCATTTTCATTCAATAAAGTTGTGGATGTACAGTTCAACAGCACTTTGGGGAAGTGTGTGAGCTACACTGAACAAGGAGTGAAACATGCAGAGAACTTCAACAATGACCCGGCCGTCTTGCAGCAGATGAAAGCTGAGGTGGACACATTCTGCAGACATAATGCTCAGATCTGGGACACAGCTGTTCGT---GATAAAGCA------------------------------------------------------------------------------------------------------------------------------------------------------------------------------------------------------------------------------------------------------------------------------------------------------------------------------------------------------------------------------------------------GTGAAACCGACGGTTATGATCAGTTCAGTAAAACAGGCTGATGGCAGTCATCCAGCTGTTCTGATGTGCAGCGCATATGAATTCTACCCCTCAAAAATCAAAGTGTCTTGGCTGAGAGATGGTAAACCGATGACCTCTGATGTGACCTCCACAATGGAGATGGCTGATGGGGACTGGTACTACCAGATTCACTCTGAGCTGGAATACACTCCTAAATCTGGGGAGAAGATCTCCTGTATGGTGGAGCACGCCAGCTTCAATAAACCCATGATTTATGACTGG-----------------------------------------------------------------------------------------------------------------------------------------------------------------------GATCCCGCTATCTCTGAGTCTGACAGGAATAAAATCGCCATCGGGGCGTCTGGTCTGGTGCTGGGGATCATCATAGCAGCTGCTGGACTCATTTACTACAAGAAGAAATCAACAGGGAGGATCCTGGTACCAAATC

>Rhsi-DAB3*01:12

CTGATGCTGTCTGCTTTTACTGGAACA-----------------------------------------------------------------------------------------------------------------------------------------------------------------------------------------------------------------------GCTGATGGATATTATCAATACGACATAGCTGAATGCTTCTACGGCACCAGTGATTACAGCGATATGGTGTATCTACGTTCATTTTCATTCAATAAAGTTGTGGATGTACAGTTCAACAGCACTTTGGGGAAGTGTGTGGGCTACACTGAACAAGGAGTGAAACATGCAGAGAACTTCAACAATGACCCGGCCGTCTTGCAGCAGATGAAAGCTGAGGTGGACACATTCTGCAGACATAATGCTCAGATCTGGGACACAGCTGTTCGT---GATAAAGCA------------------------------------------------------------------------------------------------------------------------------------------------------------------------------------------------------------------------------------------------------------------------------------------------------------------------------------------------------------------------------------------------GTGAAACCGACGGTTATGATCAGTTCAGTAAAACAGGCTGATGGCAGTCATCCAGCTGTTCTGATGTGCAGCGCATATGAATTCTACCCCTCAAAAATCAAAGTGTCTTGGCTGAGAGATGGTAAACCGATGACCTCTGATGTGACCTCCACAATGGAGATGGCTGATGGGGACTGGTACTACCAGATTCACTCTGAGCTGGAATACACTCCTAAATCTGGGGAGAAGATCTCCTGTATGGTGGAGCACGCCAGCTTCAATAAACCCATGATTTATGACTGG-----------------------------------------------------------------------------------------------------------------------------------------------------------------------GATCCCGCTATCTCTGAGTCTGACAGGAATAAAATCGCCATCGGGGCGTCTGGTCTGGTGCTGGGGATCATCATAGCAGCTGCTGGACTCATTTACTACAAGAAGAAATCAACAGGGAGGATCCTGGTACCAAATC

>Rhsi-DAB3*01:13

CTGATGCTGTCTGCTTTTACTGGAACA-----------------------------------------------------------------------------------------------------------------------------------------------------------------------------------------------------------------------GCTGATGGATATTATCAATACGACATAGCTGAATGCTTCTACAGCACCAGTGTTTACAGCGATATGGTGTATCTACGTTCATTTTCATTCAATAAAGTTGTGGATGTACAGTTCAACAGCACTTTGGGGAAGTGTGTGGGCTACACTGAACAAGGAGTGAAACATGCAGAGAACTTCAACAATGACCCGGCCGTCTTGCAGCAGATGAAAGCTGAGGTGGACACATTCTGCAGACATAATGCTCAGATCTGGGACACAGCTGTTCGT---GATAAAGCA------------------------------------------------------------------------------------------------------------------------------------------------------------------------------------------------------------------------------------------------------------------------------------------------------------------------------------------------------------------------------------------------GTGAAACCGACGGTTATGATCAGTTCAGTAAAACAGGCTGATGGCAGTCATCCAGCTGTTCTGATGTGCAGCGCATATGAATTCTACCCCTCAAAAATCAAAGTGTCTTGGCTGAGAGATGGTAAACCGATGACCTCTGATGTGACCTCCACAATGGAGATGGCTGATGGGGACTGGTACTACCAGATTCACTCTGAGCTGGAATACACTCCTAAATCTGGGGAGAAGATCTCCTGTATGGTGGAGCACGCCAGCTTCAATAAACCCATGATTTATGACTGG-----------------------------------------------------------------------------------------------------------------------------------------------------------------------GATCCCGCTATCTCTGAGTCTGACAGGAATAAAATCGCCATCGGGGCGTCTGGTCTGGTGCTGGGGATCATCATAGCAGCTGCTGGACTCATTTACTACAAGAAGAAATCAACAGGGAGGATCCTGGTACCAAATC

>Rhsi-DAB3*01:14

CTGATGCTGTCTGCATTTACTGGAACA-----------------------------------------------------------------------------------------------------------------------------------------------------------------------------------------------------------------------GCTGATGGATATTATCAATACGACATAGCTGAATGCTTCTACAGCACCAGTGATTACAGCGATATGGTGTATCTACGTTCATTTTCATTCAATAAAGTTGTGGATGTACAGTTCAACAGCACTTTGGGGAAGTGTGTGGGCTACACTGAACAAGGAGTGAAACATGCAGAGAACTTCAACAATGACCCGGCCGTCTTGCAGCAGATGAAAGCTGAGGTGGACACATTCTGCAGACATAATGCTCAGATCTGGGACACAGCTGTTCGT---GATGAAGCA------------------------------------------------------------------------------------------------------------------------------------------------------------------------------------------------------------------------------------------------------------------------------------------------------------------------------------------------------------------------------------------------GTGAAACCGACGGTTATGATCAGTTCAGTAAAACAGGCTGATGGCAGTCATCCAGCTGTTCTGATGTGCAGCGCAYATGAATTCTACCCCTCAAAAATCAAAGTGTCTTGGCTGAGAGATGGTAAACCGATGACCTCTGATGTGACCTCCACAATGGAGATGGCTGATGGGGACTGGTACTACCAGATTCACTCTGAGCTGGAATACACTCCTAAATCTGGGGAGAAGATCTCCTGTATGGTGGAGCACGCCAGCTTCAATAAACCCATGATTTATGACTGG-----------------------------------------------------------------------------------------------------------------------------------------------------------------------GATCCCGCTATCTCTGAGTCTGACAGGAATAAAATCGCCATCGGGGCGTCTGGTCTGGTGCTGGGGA

>Rhsi-DAB3*01:15

CTGATGCTGTCTGCTTTCACTGGAACA-----------------------------------------------------------------------------------------------------------------------------------------------------------------------------------------------------------------------GCTGATGGATATTATCAATACGACATAGCTGAATGCTTCTACAGCACCAGTGATTACAGCGATATGGTGTATCTACGTTCATTTTCATTCAATAAAGTTGTGGATGTACAGTTCAACAGCACTTTGGGGAGGTGTGTGGGCTACACTGAACAAGGAGTGAAACATGCAGAGAACTTCAACAATGACCCGGCCGTCTTGCAGCAGATGAAAGCTGAGGTGGACACATTCTGCAGACATAATGCTCAGATCTGGGACACAGCTGTTCGT---GATAAAGCA------------------------------------------------------------------------------------------------------------------------------------------------------------------------------------------------------------------------------------------------------------------------------------------------------------------------------------------------------------------------------------------------GTGAAACCGACGGTTATGATCAGTTCAGTAAAACAGGCTGATGGCAGTCATCCAGCTGTTCTGATGTGCAGCGCATATGAATTCTACCCCTCAAAAATCAAAGTGTCTTGGCTGAGAGATGGTAAACCGATGACCTCTGATGTGACCTCCACAATGGAGATGGCTGATGGGGACTGGTACTACCAGATTCACTCTGAGCTGGAATACACTCCTAAATCTGGGGAGAAGATCTCCTGTATGGTGGAGCACGCCAGCTTCAATAAACCCATGATTTATGACTGG-----------------------------------------------------------------------------------------------------------------------------------------------------------------------GATCCCGCTATCTCTGAGTCTGACAGGAATAAAATCGCCATCGGGGCGTCTGGTCTGGTGCTGGGGATCATCATAGCAGCTGCTGGACTCATTTACTACAAGAAGAAATCAACAGGGAGGATCCTGGTACCAAATC

>Rhsi-DAB3*01:16

CTGATGCTGTCTGCTTTTACTGGAACA-----------------------------------------------------------------------------------------------------------------------------------------------------------------------------------------------------------------------GCTGATGGATATTATCAATACGACATAGCTGAATGCTTCTACAGCACCAGTGATTACAGCGATATGGTGTATCTACGTTCATTTTCATTCAATAAAGTTGTGGATGTACAGTTCAACAGCACTTTGGGGAAGTGTGTGGGCTACACTGAACAAGGAGTGAAACGTGCAGAGAACTTCAACAATGACCCGGCCGTCTTGCAGCAGATGAAAGCTGAGGTGGACACATTCTGCAGACATAATGCTCAGATCTGGGACACAGCTGTTCGT---GATAAAGCA------------------------------------------------------------------------------------------------------------------------------------------------------------------------------------------------------------------------------------------------------------------------------------------------------------------------------------------------------------------------------------------------GTGAAACCGACGGTTATGATCAGTTCAGTAAAACAGGCTGATGGCAGTCATCCAGCTGTTCTGATGTGCAGCGCATATGAATTCTACCCCTCAAAAATCAAAGTGTCTTGGCTGAGAGATGGTAAACCGATGACCTCTGATGTGACCTCCACAATGGAGATGGCTGATGGGGACTGGTACTACCAGATTCACTCTGAGCTGGAATACACTCCTAAATCTGGGGAGAAGATCTCCTGTATGGTGGAGCACGCCAGCTTCAATAAACCCATGATTTATGACTGG-----------------------------------------------------------------------------------------------------------------------------------------------------------------------GATCCCGCTATCTCTGAGTCTGACAGGAATAAAATCGCCATCGGGGCGTCTGGTCTGGTGCTGGGGATCATCATAGCAGCTGCTGGACTCATTTACTACAAGAAGAAATCAACAGGGAGGATCCTGGTACCAAATC

>Rhsi-DAB3*02:01:01

CTGATGCTGTCGGCATTTACTGGAACA-----------------------------------------------------------------------------------------------------------------------------------------------------------------------------------------------------------------------GCTGATGGATATTATCAATACGACATAGCTGAATGCTTCTACAGCACCAGTGATTACAGCGATATGGTGTATCTACGTTCATTTTCATTCAATAAAGTTGTGGATGTACAGTTCAACAGCACTGTGGGGAAGTGTGTGGGCTACACTGAACAAGGAGTGAAACATGCAGAGAACTTCAACAAAGATCAGGCCATCATGCAGCAGTTAAAATCTGAGGTGGACAGATTCTGCAGACACAATGCTCATAACCATGACTCAGCTGTTCGT---GATAAAGCA------------------------------------------------------------------------------------------------------------------------------------------------------------------------------------------------------------------------------------------------------------------------------------------------------------------------------------------------------------------------------------------------GTGAAACCGACGGTTATGATCAGTTCAGTAAAACAGGCTGATGGCAGTCATCCAGCTGTTCTGATGTGCAGCGCATATGAATTCTACCCCTCAAAAATCAAAGTGTCTTGGCTGAGAGATGGTAAACCGATGACCTCTGATGTGACCTCCACAATGGAGATGGCTGATGGGGACTGGTACTACCAGATTCACTCTGAGCTGGAATACACTCCTAAATCTGGGGAGAAGATCTCCTGTATGGTGGAGCACGCCAGCTTCAATAAACCCATGATTTATGACTGG-----------------------------------------------------------------------------------------------------------------------------------------------------------------------GATCCCGCTATCTCTGAGTCTGACAGGAATAAAATCGCCATCGGAGCGTCTGGTCTGGTGCTGGGGATCATCATAGCAGCTGCTGGACTCATTTACTACAAGAAGAAATCAACAGGGAGGATCCTGGTACCAAA

>Rhsi-DAB3*02:01:02

CTGATGCTGTCGGCTTTTACCGGAACA-----------------------------------------------------------------------------------------------------------------------------------------------------------------------------------------------------------------------GCTGATGGATATTATCAATACGACATAGCTGAATGCTTCTACAGCACCAGTGATTACAGCGATATGGTGTATCTACGTTCATTTTCATTCAATAAAGTTGTGGATGTACAGTTCAACAGCACTGTGGGGAAGTGTGTGGGCTACACTGAACAAGGAGTGAAACATGCAGAGAACTTCAACAAAGATCAGGCCATCATGCAGCAGTTAAAATCTGAGGTGGACAGATTCTGCAGACATAATGCTCATAACCATGACTCAGCTGTTCGT---GATAAAGCA------------------------------------------------------------------------------------------------------------------------------------------------------------------------------------------------------------------------------------------------------------------------------------------------------------------------------------------------------------------------------------------------GTGAAACCGACGGTTATGATCAGTTCAGTAAAACAGGCTGATGGCAGTCATCCAGCTGTTCTGATGTGCAGCGCATATGAATTCTACCCCTCAAAAATCAAAGTGTCTTAGCTGAGAGATGGTAAACCGATGACCTCTGATGTGACCTCCACAATGGAGATGGCTGATGGGGACTGGTACTACCAGATTCACTCTGAGCTGGAATACACTCCTAAATCTGGGGAGAAGATCTCCTGTATGGTGGAGCACGCCAGCTTCAATAAACCCATGATTTATGACTGG-----------------------------------------------------------------------------------------------------------------------------------------------------------------------GATCCCGCTATCTCTGAGTCTGACAGGAATAAAATCGCCATCGGAGCGTCTGGTCTGGTGCTGGGGATCATCATAGCAGCTGCTGGACTCATTTACTACAAGAAGAAATCAACAGGGAGGATCCTGGTACCAAA

>Rhsi-DAB3*02:02

CTGATGCTGTCTACTTTCACTGGAACA-----------------------------------------------------------------------------------------------------------------------------------------------------------------------------------------------------------------------GCTGATGGATATTATCAATACGACATAGCTGAATGCTTCTTCAGCACCAGTGATTACAGCGATATGGTGTATCTACGTTCATTTTCATTCAATAAAGTTGTGGATGTACAGCTCAACAGCACTGTGGGGAAGTGTGTGGGCTACACTGAACAAGGAGTGAAACATGCAGAGAACTTCAACAAAGATCAGGCCATCATGCAGCAGTTAAAATCTGAGGTGGACAGATTCTGCAGACATAATGCTCATAACCATGACTCAGCTGTTCGT---GATAAAGCA------------------------------------------------------------------------------------------------------------------------------------------------------------------------------------------------------------------------------------------------------------------------------------------------------------------------------------------------------------------------------------------------GTGAAACCGACGGTTATGATCAGTTCAGTAAAACAGGCTGATGGCAGTCATCCAGCTGTTCTGATGTGCAGCGCATATGAATTCTACCCCTCAAAAATCAAAGTGTCTTGGCTGAGAGATGGTAAACCGATGACCTCTGATGTGACCTCCACAATGGAGATGGCTGATGGGGACTGGTACTACCAGATTCACTCTGAGCTGGAATACACTCCTAAATCAGGGGAGAAGATCTCCTGTATGGTGGAGCACGCCAGCTTCAATAAACCCATGATTTATGACTGG-----------------------------------------------------------------------------------------------------------------------------------------------------------------------GATCCCGCTATCTCTGAGTCTGACAGGAATAAAATCGCCATCGGAGCGTCTGGTCTGGTGCTGGGGATCATCATAGCAGCTGCTGGACTCATTTACTACAAGAAGAAATCAACAGGGAGGATCCTGGTACCAAA

>Rhsi-DAB3*03:01:01

CTGATGCTGTCGGCATTCACCGGAACA-----------------------------------------------------------------------------------------------------------------------------------------------------------------------------------------------------------------------GCTGATGGATATTATGAATACGACACAGCTGAATGCTTCTACAGCACCAGTGATTACAGTGATATGGTGTATCTTTATTCATTATCATTTAATAAAGTTGTGGATGTACAGTTCAACAGCTCTCTGGGGAAGTGTGTGGGCTACACTGAACAAGGAGTGAAACATGCAGAGAACTTCAACAAAAACCCAGCCGTCATGCAGCAGCTGAAAGCTCAGGTGGACACATTCTGCAGACATAATGCTCAGATCTATGACACAGCTGTCCGT---GATAAATCA------------------------------------------------------------------------------------------------------------------------------------------------------------------------------------------------------------------------------------------------------------------------------------------------------------------------------------------------------------------------------------------------GTGAAACCAAAGGTTAAGATCAGTTCAGTGAAGCGGGCTGGTGGCAAACATCCAGCTGAGTTGATGTGCAGCGCTTATGAATTCTACCCCAAAAAAATCCAAATGTATTGGCTAAAAGACGGTAACAAGGTGACCACAGAAGTGACTTCCACAATGGAGATGGCTGATGGGGACTGGTTCTACCAGATTCACTCTGAGCTGGAATACACTCCTAAATCTGGGGAGAAGATCTCCTGTGTGGTGGAGCACGCCAGCTTCAATAAACCCATGGTTTATGACTGG-----------------------------------------------------------------------------------------------------------------------------------------------------------------------GATCCCGCGATCTCTGAGTCTGACAGAAATAAAATCGCCATCGGGGCGTCTGGTCTGGTGCTGGGGATCATCATAGCAGCTGCTGGACTCATTTACTATAAGAAGAAATCAACAGGGAGGATCCTGGTACCAAATC

>Rhsi-DAB3*03:01:02

CTGATGCTGTCGGCATTTACTGGAACA-----------------------------------------------------------------------------------------------------------------------------------------------------------------------------------------------------------------------GCTGATGGATATTATGAATACGACACAGCTGAATGCTTCTACAGCACCAGTGATTACAGTGATATGGTGTATCTTTATTCATTATCGTTTAATAAAGTTGTGGATGTACAGTTCAACAGCTCTCTGGGGAAGTGTGTGGGCTACACTGAACAAGGAGTGAAACATGCAGAGAACTTCAACAAAAACCCAGCCGTCATGCAGCAGCTGAAAGCTCAGGTGGACACATTCTGCAGACATAATGCTCAGATCTATGACACAGCTGTCCGT---GATAAATCA------------------------------------------------------------------------------------------------------------------------------------------------------------------------------------------------------------------------------------------------------------------------------------------------------------------------------------------------------------------------------------------------GTGAAACCAAAGGTTAAGATCAGTTCAGTGAAGCGGGCTGGTGGCAAACATCCAGCTGAGTTGATGTGCAGCGCTTATGAATTCTACCCCAAAAAAATCCAAATGTATTGGCTAAAAGACGGTAACAAGGTGACCACAGAAGTGACTTCCACAATGGAGATGGCTGATGGGGACTGGTTCTACCAGATTCACTCTGAGCTGGAATACACTCCTAAATCTGGGGAGAAGATCTCCTGTGTGGTGGAGCACGCCAGCTTCAATAAACCCATGGTTTATGACTGG-----------------------------------------------------------------------------------------------------------------------------------------------------------------------GATCCCGCGATCTCTGAGTCTGACAGAAATAAAATCGCCATCGGGGCGTCTGGTCTGGTGCTGGGGATCATCATAGCAGCTGCTGGACTCATTTACTATAAGAAGAAATCAACAGGGAGGATCCTGGTACCAAATC

>Rhsi-DAB3*03:02

CTGATGCTGTCTGCTTTTACTGGAACA-----------------------------------------------------------------------------------------------------------------------------------------------------------------------------------------------------------------------GCTGATGGATATTATGAATACGACTCAGCTGAATGCTTCTACAGCACCAGTGATTACAGTGATATGGTGTATCTTTATTCATTATCATTTAATAAAGTTGTGGATGTACAGTTCAACAGCTCTCTGGGGAAGTGTGTGGGCTACACTGAACAAGGAGTGAAACATGCAGAGAACTTCAACAAAAACCCAGCCGTCATGCAGCAGCTGAAAGCTCAGGTGGACACATTCTGCAGACATAATGCTCAGATCTATGACACAGCTGTCCGT---GATAAATCA------------------------------------------------------------------------------------------------------------------------------------------------------------------------------------------------------------------------------------------------------------------------------------------------------------------------------------------------------------------------------------------------GTGAAACCAAAGGTTAAGATCAGTTCAGTGAAGCGGGCTGGTGGCAAACATCCAGCTGAGTTGATGTGCAGCGCTTATGAATTCTACCCCAAAAAAATCCAAATGTATTGGCTAAAAGACGGTAACAAGGTGACCACAGAAGTGACTTCCACAATGGAGATGGCTGATGGGGACTGGTTCTACCAGATTCACTCTGAGCTGGAATACACTCCTAAATCTGGGGAGAAGATCTCCTGTGTGGTGGAGCACGCCAGCTTCAATAAACCCATGGTTTATGACTGG-----------------------------------------------------------------------------------------------------------------------------------------------------------------------GATCCCGCGATCTCTGAGTCTGACAGAAATAAAATCGCCATCGGGGCGTCTGGTCTGGTGCTGGGGATCATCATAGCAGCTGCTGGACTCATTTACTATAAGAAGAAATCAACAGGGAGGATCCTGGTACCAAATC

>Rhsi-DAB3*03:03

CTGATGCTGTCTGCTTTTACTGGAACA-----------------------------------------------------------------------------------------------------------------------------------------------------------------------------------------------------------------------GCTGATGGATATTATGAATACGACACAGCTGAATGCTTCTACAGCACCAGTGATTACGGTGATATGGTGTATCTTTATTCATTATCGTTTAATAAAGTTGTGGATGTACAGTTCAACAGCTCTCTGGGGAAGTGTGTGGGCTACACTGAACAAGGAGTGAAACATGCAGAGAACTTCAACAAAAACCCAGCCGTCATGCAGCAGCTGAAAGCTCAGGTGGACACACTCTGCAGACATAATGCTCAGATCTATGACACAGCTGTCCGT---GATAAATCA------------------------------------------------------------------------------------------------------------------------------------------------------------------------------------------------------------------------------------------------------------------------------------------------------------------------------------------------------------------------------------------------GTGAAACCAAAGGTTAAGATCAGTTCAGTGAAGCGGGCTGGTGGCAAACATCCAGCTGAGTTGATGTGCAGCGCTTATGAATTCTACCCCAAAAAAATCCAAATGTATTGGCTAAAAGACGGTAACAAGGTGACCACAGAAGTGACTTCCACAATGGAGATGGCTGATGGGGACTGGTTCTACCAGATTCACTCTGAGCTGGAATACACTCCTAAATCTGGGGAGAAGATCTCCTGTGTGGTGGAGCACGCCAGCTTCAATAAACCCATGGTTTATGACTGG-----------------------------------------------------------------------------------------------------------------------------------------------------------------------GATCCCGCGATCTCTGAGTCTGACAGAAATAAAATCGCCATCGGGGCGTCTGGTCTGGTGCTGGGGATCATCATAGCAGCTGCTGGACTCATTTACTATAAGAAGAAATCAACAGGGAGGAAATCACTAGTGAATT

>Rhsi-DAB3*03:04

CTGATGCTGTCTGCATTTACTGGAACA-----------------------------------------------------------------------------------------------------------------------------------------------------------------------------------------------------------------------GCTGATGGATATTATGAATACGACACAGCTGAATGCTTCTACAGCACCAGTGATTACAGTGATATGGTGTATCTTTATTCATTATCATTTAATAAAGTTGTGGATGTACTGTTCAACAGCTCTCTGGGGAAGTGTGTGGGCTACACTGAACAAGGAGTGAAACATGCAGAGAACTTCAACAAAAGCCCAGCCGTCATGCAGCAGCTGAAAGCTCAGGTGGACACATTCTGCAGACATAATGCTCAGATCTATGACACAGCTGTCCGT---GATAAATCA------------------------------------------------------------------------------------------------------------------------------------------------------------------------------------------------------------------------------------------------------------------------------------------------------------------------------------------------------------------------------------------------GTGAAACCAAAGGTTAAGATCAGTTCAGTGAAGCGGGCTGGTGGCAAACATCCAGCTGAGTTGATGTGCAGCGCTTATGAATTCTACCCCAAAAAAATCCAAATGTATTGGCTAAAAGACGGTAACAAGGTGACCGCAGAAGTGACTTCCACAATGGAGATGGCTGATGGGGACTGGTTCTACCAGATTCACTCTGAGCTGGAATACACTCCTAAATCTGGGGAGAAGATCTCCTGTGTGGTGGAGCACGCCAGCTTCAATAAACCCATGGTTTATGACTGG-----------------------------------------------------------------------------------------------------------------------------------------------------------------------GATCCCGCGATCTCTGAGTCTGACAGAAATAAAATCGCCATCGGGGCGTCTGGTCTGGTGCTGGGGATCATCATAGCAGCTGCTGGACTCATTTACTATAAGAAGAAATCAACAGGGAGGATCCTGGTACCAAATC

>Rhsi-DAB3*03:05

CTGATGCTGTCGGCTTTCACTGGAACA-----------------------------------------------------------------------------------------------------------------------------------------------------------------------------------------------------------------------GCTGATGGATATTATGAATACGACACAGCTGAATGCTTCTACAGCACCAGTGATTACAGTGATATGGTGTATCTTTATTCATTATCATTTAATAAAGTTGTGGATGTACAGTCCAACAGCTCTCTGGGGAAGTGTGTGGGCTACACTGAACAAGGAGTGAAACATGCAGAGAACTTCAACAAAAACCCAGCCGTCATGCAGCAGCTGAAAGCTCAGGTGGACACATTCTGCAGACATAATGCTCAGATCTATGACACAGCTGTCCGT---GATAAATCA------------------------------------------------------------------------------------------------------------------------------------------------------------------------------------------------------------------------------------------------------------------------------------------------------------------------------------------------------------------------------------------------GTGAAACCAAAGGCTAAGATCAGTTCAGTGAAGCGGGCTGGTGGCAAACATCCAGCTGAGTTGATGTGCAGCGCTTATGAATTCTACCCCAAAAAAATCCAAATGTATTGGCTAAAAGACGGTAACAAGGTGACCACAGAAGTGACTTCCACAATGGAGATGGCTGATGGGGACTGGTTCTACCAGATTCACTCTGAGCTGGAATACACTCCTAAATCTGGGGAGAAGATCTCCTGTGTGGTGGAGCACGCCAGCTTCAATAAACCCATGGTTTATGACTGG-----------------------------------------------------------------------------------------------------------------------------------------------------------------------GATCCCGCGATCTCTGAGTCTGACAGAAATAAAATCGCCATCGGGGCGTCTGGTCTGGTGCTGGGGATCATCATAGCAGCTGCTGGACTCATTTACTATAAGAAGAAATCAACAGGGAGGATCCTGGTACCAAATC

>Rhsi-DAB3*03:06

CTGATGCTGTCTACTTTTACTGGAACA-----------------------------------------------------------------------------------------------------------------------------------------------------------------------------------------------------------------------GCTGATGGATGTTATGAATACGACACAGCTGAATGCTTCTACAGCACCAGTGATTACAGTGATATGGTGTATCTTTATTCATTATCATTTAATAAAGTTGTGGATGTACAGTTCAACAGCTCTCTGGGGAAGTGTGTGGGCTACACTGAACAAGGAGTGAAACATGCAGAGAACTTCAACAAAAACCCAGCCGTCATGCAGCAGCTGAAAGCTCAGGTGGACACATTCTGCAGACATAATGCTCAGATCTATGACACAGCTGTCCGT---GATAAATCA------------------------------------------------------------------------------------------------------------------------------------------------------------------------------------------------------------------------------------------------------------------------------------------------------------------------------------------------------------------------------------------------GTGAAACCAAAGGTTAAGATCAGTTCAGTGAAGCGGGCTGGTGGCAAACATCCAGCTGAGTTGATGTGCAGCGCTTATGAATTCTACCCCAAAAAAATCCAAATGTATTGGCTAAAAGACGGTAACAAGGTGACCACAGAAGTGACTTCCACAATGGAGATGGCTGATGGGGACTGGTTCTACCAGATTCACTCTGAGCTGGAATACACTCCTAAATCTGGGGAGAAGATCTCCTGTGTGGTGGAGCACGCCAGCTTCAATAAACCCATGGTTTATGACTGG-----------------------------------------------------------------------------------------------------------------------------------------------------------------------GATCCCGCGATCTCTGAGTCTGACAGAAATAAAATCGCCATCGGGGCGTCTGGTCTGGTGCTGGGGATCATCATAGCAGCTGCTGGACTCATTTACTATAAGAAGAAATCAACAGGGAGGATCCTGGTACCAAATC

>Rhsi-DAB3*03:07

CTGATGCTGTCTACTTTTACTGGAACA-----------------------------------------------------------------------------------------------------------------------------------------------------------------------------------------------------------------------GCTGATGGATATTATGAATACGACACAGCTGAATGCTTCTACAGCACCAGTGATTACAGTGATATGGTGTATCTTTATTCATTATCATTTAATAAAGTTGTGGATGTACAGTTCAACAGCTCTCTGGGGAAGTGTGTGGGCTACACTGAACAAGGAGTGAAACATGCAGAGAACTTCAACAAAAACCCAGCCGTCATGCAGCAGCTGAAAGCTCAGGTGGACACATTCTGCAGACATAATGCTCAGATCTATGACACAGCTATCCGT---GATAAATCA------------------------------------------------------------------------------------------------------------------------------------------------------------------------------------------------------------------------------------------------------------------------------------------------------------------------------------------------------------------------------------------------GTGAAACCAAAGGTTAAGATCAGTTCAGTGAAGCGGGCTGGTGGCAAACATCCAGCTGAGTTGATGTGCAGCGCTTATGAATTCTACCACAAAAAAATCCAAATGTATTGGCTAAAAGACGGTAACAAGGTGACCACAGAAGTGACTTCCACAATGGAGATGGCTGATGAGGACTGGTTCTACCAGATTCACTCTGAGCTGGAATACACTCCTAAATCTGGGGAGAAGATCTCCTGTGTGGTGGAGCACGCCAGCTTCAATAAACCCATGGTTTATGACTGG-----------------------------------------------------------------------------------------------------------------------------------------------------------------------GATCCCGCGATCTCTGAGTCTGACAGAAATAAAATCGCCATCGGGGCGTCTGGTCTGGTGCTGGGGATCATCATAGCAGCTGCTGGACTCATTTACTATAAGAAGAAATCAACAGGGAGGATCCTGGTACCAAATC

>Rhsi-DAB3*04:01

CTGATGCTGTCTGCTTTTACTGGAACA-----------------------------------------------------------------------------------------------------------------------------------------------------------------------------------------------------------------------GCTGATGGATATTATGAATACGACATAGCTGAATGCTTCTACAGCACCAGTGATTACAGCGATATGGTGTATCTTCGTTCATATTCATTTAATAAAGTTGTGGATGTACAGTTCAACAGCACTGTGGGGAAGTTTGTGGGGTACACTGAGGAAGGAGTGATGTATGCAAAGAACTGGAACAACGACCCGGCCGTCTTGCAGCAGAACAAAGCTGAGGTGGACAGATTCTGCAGACATAATGCTCAGCTCTTTGACTCAGATGTCCGT---GATAAAGCA------------------------------------------------------------------------------------------------------------------------------------------------------------------------------------------------------------------------------------------------------------------------------------------------------------------------------------------------------------------------------------------------GTGAAACCGAAGGTTAAGCTCAATTCAGTGACACGTGCTGATGGCAGACATCCAGCTGTTCTGATGTGCAGCGCATATGAATTCTACCCCAAAAAAATCCAAGTGTCTTGGCTGAGAGATGGTACACCGATGACCTCAGAAGTGACCTCGACAATGGAGATGGCTGATGGGAATTGGTTCTACCAGATTCACTCTGAGCTGGAATACACTCCTAAATCTGGGGAGAAGATCTCCTGTGTGGTGGAGCACGCCAGCTTCAATAAACCCATGATTTATGACTGG-----------------------------------------------------------------------------------------------------------------------------------------------------------------------AATCCCGCGATCTCTGAGTCTGACAGGAATAAAATCGCCATCGGGGCGTCTGGTCTGGCGCTGGGGATCATCATAGCAGCTGCTGGACTCATTTATTATAAGAAGAAATCAACAGGGAGGATCCTGGTACCAAATC

>Rhsi-DAB3*04:02

CTGATGCTGTCTGCTTTCACTGGAACA-----------------------------------------------------------------------------------------------------------------------------------------------------------------------------------------------------------------------GCTGATGGATATTATGAATACGACATAGCTGAATGCTTCTACAGCACCAGTGGTTACAGCGATATGGTGTATCTTCGTTCATATTCATTTAATAAAGTTGTGGATGTACAGTTCAACAGCACTGTGGGGAAGTTTGTGGGGTACACTGAGGAAGGAGTGATGTATGCAAAGAACTGGAACAACGACCCGGCCGTCTTGCAGCAGAACAAAGCTGAGGTGGACAGATTCTGCAGACATAATGCTCAGCTCTTTGACTCAGATGTCCGT---GATAAAGCA------------------------------------------------------------------------------------------------------------------------------------------------------------------------------------------------------------------------------------------------------------------------------------------------------------------------------------------------------------------------------------------------GTGAAACCGAAGGTTAAGCTCAATTCAGTGACACGTGCTGATGGCAGACATCCAGCTGTTCTGATGTGCAGCGCATATGAATTCTACCCCAAAAAAATCCAAGTGTCTTGGCTGAGAGATGGTACACCGATGACCTCAGAAGTGACCTCGACAATGGAGATGGCTGATGGGAATTGGTTCTACCAGATTCACTCTGAGCTGGAATACACTCCTAAATCTGGGGAGAAGATCTCCTGTGTGGTGGAGCACGCCAGCTTCAATAAACCCATGATTTATGACTGG-----------------------------------------------------------------------------------------------------------------------------------------------------------------------AATCCCGCGATCTCTGAGTCTGACAGGAATAAAATCGCCATCGGGGCGTCTGGTCTGGTGCTGGGGATCATCATAGCAGCTGCTGGACTCATTTATTATAAGAAGAAATCAACAGGGAGGATCCTGGTACCAAATC

>Rhsi-DAB3*04:03

CTGATGCTGTCGGCTTTTACTGGAACA-----------------------------------------------------------------------------------------------------------------------------------------------------------------------------------------------------------------------GCTGATGGATATTATGAATACGACATAGCTGAATGCTTCTACAGCACCAGTGATTACAGCGATATGGTGTATCTTCGTTCATATTCATTTAATAAAGTTGTGGATGTACAGTTCAACAGCACTGTGGGGAAGTTTGTGGGGTACACTGAGGAAGGAGTGATGTATGCAAAGAACTGGAACAACGACCCGGCCGTCTTGCAGCAGAACAAAGCTGAGGTGGACAGATTCTGCAGACATAATGCTCAGCTCTTTGACCCAGATGTCCGT---GATAAAGCA------------------------------------------------------------------------------------------------------------------------------------------------------------------------------------------------------------------------------------------------------------------------------------------------------------------------------------------------------------------------------------------------GTGAAACCGAAGGTTAAGCTCAATTCAGTGACACGTGCTGATGGCAGACATCCAGCTGTTCTGATGTGCAGCGCATATGAATTCTACCCCAAAAAAATCCAAGTGTCTTGGCTGAGAGATGGTACACCGATGACCTCAGAAGTGACCTCGACAATGGAGATGGCTGATGGGAATTGGTTCTACCAGATTCACTCTGAGCTGGAATACACTCCTAGATCTGGGGAGAAGATCTCCTGTGTGGTGGAGCACGCCAGCTTCAATAAACCCATGATTTATGACTGG-----------------------------------------------------------------------------------------------------------------------------------------------------------------------AATCCCGCGATCTCTGAGTCTGACAGGAATAAAATCGCCATCGGGGCGTCTGGTCTGGTGCTGGGGA

>Rhsi-DAB3*04:04

CTGATGCTGTCTGCTTTCACCGGAACA-----------------------------------------------------------------------------------------------------------------------------------------------------------------------------------------------------------------------GCTGATGGATATTATGAATACGACATAGCTGAATGCTTCTACAGCACCAGTGATTACAGCGATATGGTGTATCTTCGTTCATATTCATTTAATAAAGTTGTGGGTGTACAGTTCAACAGCACTGTGGGGAAGTTTGTGGGGTACACTGAGGAAGGAGTGATGTATGCAAAGAACTGGAACAACGACCCGGCCGTCTTGCAGCAGAACAAAGCTGAGGTGGACAGATTCTGCAGACATAATGCTCAGCTCTTTGACTCAGATGTCCGT---GATAAAGCA------------------------------------------------------------------------------------------------------------------------------------------------------------------------------------------------------------------------------------------------------------------------------------------------------------------------------------------------------------------------------------------------GTGAAACCGAAGGTTAAGCTCAATTCAGTGACACGTGCTGATGGCAGACATCCAGCTGTTCTGATGTGCAGCGCATATGAATTCTACCCCAAAAAAATCCAAGTGTCTTGGCTGAGAGATGGTACACCGATGACCTCAGAAGTGACCTCGACAATGGAGATGGCTGATGGGAATTGGTTCTACCAGATTCACTCTGAGCTGGAATACACTCCTAAATCTGGGGAGAAGATCTCCTGTGTGGTGGAGCACGCCAGCTTCAATAAACCCATGATTTATGACTGG-----------------------------------------------------------------------------------------------------------------------------------------------------------------------AATCCCGCGATCTCTGAGTCTGACAGGAATAAAATCGCCATCGGGGCGTCTGGTCTGGTGCTGGGGATCATCATAGCAGCTGCTGGACTCATTTATTATAAGAAGAAATCAACAGGGAGGATCCTGGTACCAAATC

>Rhsi-DAB3*05:01

CTGATGCTGTCTGCTTTTACTGGAACA-----------------------------------------------------------------------------------------------------------------------------------------------------------------------------------------------------------------------GCTGATGGATATTATGAATACGACATATCTGAATGCTTCTACAGCACCAGTGATTACAGCGATATGGTGTATCTTCATTCATATTCATTCAATAAAGTTGTGGATGTACAGTTCAACAGCTCTGTGGGGAAGTATGTGGGCTACACTGAGGAAGGAGTGATGTATGCAAAGAACTGGAACAACAACCCGGCCGTCATGCAGCAGGAGAAAGCTATGGTGGACACATTCTGCAGAAATAATGCTCAGATCTCTGACTCAGCTGTCCGT---GATAAAGCA------------------------------------------------------------------------------------------------------------------------------------------------------------------------------------------------------------------------------------------------------------------------------------------------------------------------------------------------------------------------------------------------GTGAAACCGAAGGTTAAGCTCAATTCAGTGACACGTGCTGATGGCAGACATCCAGCTGTCCTGATGTGCAGCGCATATGAATTCTACCCCTCAAAAATCAAAGTGTCATGGCTGAGAGATGGTACACCGATGACCTCAGAAGTGACCTCCACAATGGAGATGGCTGATGGGAATTGGTTCTACCAGATTCACTCTGAGCTGGAATGCACTCCTAAATCTGGGGAGAAGATCTCCTGTGTGGTGGAGCACGCCAGCTTCAATAAACCCATGATTTATGACTGG-----------------------------------------------------------------------------------------------------------------------------------------------------------------------AATCCCGCGATCTCTGAGTCTGACAGGAATAAAATCGCCATCGGGGCGTCTGGTCTGGTGCTGGGGATCATCATAGCAGCTGCTGGACTCATTTATTATAAGAAGAAATCAACAGGGAGGATCCTGGTACCAAA

>Rhsi-DAB3*05:02

CTGATGCTGTCGGCATTTACTGGAACA-----------------------------------------------------------------------------------------------------------------------------------------------------------------------------------------------------------------------GCTGATGGATATTATGAATACGACATATCTGAATGCTTCTACAGCACCAGTGATTACAGCGATATGGTGTATCTTCGTTCATATTCATTCAATAAAGTTGTGGATGTACAGTTCAACAGCTCTGTGGGGAAGTATGTGGGCTACACTGAGGAAGGAGTGATGTATGCAAAGAACTGGAACAACGACCCGGTCGGCTTGCAGCAGGATAAAGCTCAGGTGGACACATTCTGCAGACATAATGCTCAGATCTCTGACTCAGCTGTCCGT---GATAAAGCA------------------------------------------------------------------------------------------------------------------------------------------------------------------------------------------------------------------------------------------------------------------------------------------------------------------------------------------------------------------------------------------------GTGAAACCGAAGGTTAAGCTCGATTCAGTGACACGTGCTGATGGCAGACATCCAGCTGTTCTGATGTGCAGCGCATATGAATTCTACCCCCCAAAAATCCAAGTGTCTTGGCTGAGAGATGGTACACCGGTGACCTCAGAAGTGACCTCCACAATGGAGATGGCTGATGGGAATTGGTTCTACCAAATTCACTCTGAGCTGGAATACACTCCTAAATCTGGGGAGAAGATCTCCTGTGTGGTGGAGCACGCCAGCTTCAATAAACCCATGATTTATGACTGG-----------------------------------------------------------------------------------------------------------------------------------------------------------------------GATCCCGCGATCTCTGAGTCTGACAGGAATAAAATCGCCATCGGGGCGTCTGGTCTGGTCCTGGGGATCATCATAGCAGCTGCTGGACTCATTTATTATAAGAAGAAATCAACAGGGAGGATCCTGGTACCAAATC

>Rhsi-DAB3*05:03

CTGATGCTGTCTGCTTTCACTGGAACA-----------------------------------------------------------------------------------------------------------------------------------------------------------------------------------------------------------------------GCTGATGGATATTATGAATACGACATATCTGAATGCTTCTACAGCACCAGTGATTACAGCGATATGGTGTATCTTCGTTCATATTCATTCAATAAAGTTGTGGATGTACAGTTCAACAGCTCTGTGGGGTAGTATGTGGGCTACACTGAGGAAGGAGTGATGTATGCAAAGAACTGGAACAACGACCCGGTCGGCTTGCAGCAGGGTAAAGCTCAGGTGGACACATTCTGCAGACATAATGCTCAGATCTCTGACTCAGCTGTCCGT---GATAAAGCA------------------------------------------------------------------------------------------------------------------------------------------------------------------------------------------------------------------------------------------------------------------------------------------------------------------------------------------------------------------------------------------------GTGAAACCGAAGGTTAAGCTCAATTCAGTGACACGTGCTGATGGCAGACATCCAGCTGTTCTGATGTGCAGCGCATATGAATTCTACCCCCCAAAAATCCAAGTGTCTTGGCTGAGAGATGGTACACCGATGACCTCAGAAGTGACCTCCACAATGGAGATGGCTGATGGGAATTGGTTCTACCAAATTCACTCTGAGCTGGAATACACTCCTAAATCTGGGGAGAAGATCTCCTGTGTGGTGGAGCACGCCAGCTTCAATAAACCCATGATTTATGACTGG-----------------------------------------------------------------------------------------------------------------------------------------------------------------------GATCCCGCGATCTCTGAGTCTGACAGGAATAAAATCGCCATCGGGGCGTCTGGTCTGGTCCTGGGGATCATCATAGCAGCTGCTGGACTCATTTATTATAAGAAGAAATCAACAGGGAGGATCCTGGTACCAAATC

>Rhsi-DAB3*06:01

CTGATGCTGTCTGCTTTTACCGGAACA-----------------------------------------------------------------------------------------------------------------------------------------------------------------------------------------------------------------------GCTGATGGATATTATGAATACCAGATGAGTGAATGCGTCTACAGCACCAGTGATTACAGTGATATGGTGTATCTTATTTCATTTTCATTTAATAAAGTTGTGGATGTACAGTTCAACAGCTCTCTGGGGAAGTGTGTGGGCTACACTGAGGAAGGAGTGAAATATGCAGAGAACTGGAACAACAACCCGTCCGTCTTGCAGCAGATGAAAGCTGAGGTGGAATCA---TGCAGACATAATGCTCAGAACATTGACTCCGCTATCCTT---GATAAAGCA------------------------------------------------------------------------------------------------------------------------------------------------------------------------------------------------------------------------------------------------------------------------------------------------------------------------------------------------------------------------------------------------GTGAAACCAAAGGTTAAGATCAGTCCAGTGAAGCAGGCTGGTGGCAGACATCCAGCTGAGTTGATGTGCAGCGCATACGAATTCTACCCCAAAAAAATCCAAATGTATTGGCTGAGAGACGGTAACAAGGTGACCACAGAAGTGACTTCCACAATGGAGATGGCTAATGGGAACTGGTTCTACCAGATTCACTCTGAGCTGGAATACACTCCTAAATCTGGGGAGAAGATCTCCTGTGTGGTGGAGCATGCCAGCTTCAATAAACCCATGTTTTATGACTGG-----------------------------------------------------------------------------------------------------------------------------------------------------------------------GATCCTGCAATCTCTGAGTCTGACAGGAATAAAATCGCCATCGGGGCATCTGGTCTGGTGCTGGGGATCATCATAGCAGCTGCTGGACTCATTTACTACAAGAAGAAATCAACAGGGAGGATCCTGGTACCAAA

>Rhsi-DAB3*06:02

CTGATGCTGTCTGCTTTTACTGGAACA-----------------------------------------------------------------------------------------------------------------------------------------------------------------------------------------------------------------------GCTGATGGATATTATGAATACCAGATGAGTGAATGCGTCTACAGCACCAGTGATTACAGTGATATGGTGTATCTTATTTCATTTTCATTTAATAAAGTTGTGGATGTACAGTTCAACAGCTCTCTGGGGAAGTGTGTGGGCTACACTGAGGAAGGAGTGAAATATGCAGAGAACTGGAACAACAACCCGTCCGTCTTGCGGCAGATGAAAGCTGAGGTGGAATCA---TGCAGACATAATGCTCAGAACATTGACTCCGCTATCCTT---GATAAAGCA------------------------------------------------------------------------------------------------------------------------------------------------------------------------------------------------------------------------------------------------------------------------------------------------------------------------------------------------------------------------------------------------GTGAAACCAAAGGTTAAGATCAGTCCAGTGAAGCAGGCTGGTGGCAGACATCCAGCTGAGTTGATGTGCAGCGCATATGAATTCTACCCCAAAAAAATCCAAATGTATTGGCTGAGAGACGGTAACAAGGTGACCACAGAAGTGACTTCCACAATGGAGATGGCTAATGGGAACTGGTTCTACCAGATTCACTCTGAGCTGGAATACACTCCTAAATCTGGGGAGAAGATCTCCTGTGTGGTGGAGCATGCCAGCTTCAATAAACCCATGTTTTATGACTGG-----------------------------------------------------------------------------------------------------------------------------------------------------------------------GATCCTGCAATCTCTGAGTCTGACAGGAATAAAATCGCCATCGGGGCATCTGGTCTGGTGCTGGGGATCATCATAGCAGCTGCTGGACTCATTTACTACAAGAAGAAATCAACAGGGAGGATCCTGGTACCAAA

>Rhsi-DAB3*06:03

CTGATGCTGTCGGCTTTCACTGGAACA-----------------------------------------------------------------------------------------------------------------------------------------------------------------------------------------------------------------------GCTGATGGATATTATGGATACCAGATGAGTGAATGCGTCTACAGCACCAGTGATTACAGTGATATGGTGTATCTTATTTCATTTTCATTTAATAAAGTTGTGGATGTACAGTTCAACAGCTCTCTGGGGAAGTGTGTGGGCTACACTGAGGAAGGAGTGAAATATGCAGAGAACTGGAACAACAACCCGTCCGTCTTGCAGCAGATGAAAGCTGAGGTGGAATCA---TGCAGACATAATGCTCAGAACATTGACTCCGCTATCCTT---GATAAAGCA------------------------------------------------------------------------------------------------------------------------------------------------------------------------------------------------------------------------------------------------------------------------------------------------------------------------------------------------------------------------------------------------GTGAAACCAAAGGTTAAGATCAGTCCAGTGAAGCAGGCTGGTGGCAGACATCCAGCTGAGTTGATGTGCAGCGCATATGAATTCTACCCCAAAAAAATCCAAATGTATTGGCTGAGAGACGGTAACAAGGTGACCACAGAAGTGACTTCCACAATGGAGATGGCTAATGGGAACTGGTTCTACCAGATTCACTCTGAGCTGGAATACACTCCTAAATCTGGGGAGAAGATCTCCTGTGTGGTGGAGCATGCCAGCTTCAATAAACCCATGTTTTATGACTGG-----------------------------------------------------------------------------------------------------------------------------------------------------------------------GATCCTGCAATCTCTGAGTCTGACAGGAATAAAATCGCCATCGGGGCATCTGGTCTGGTGCTGGGGATCATCATAGCAGCTGCTGGACTCATTTACTACAAGAAGAAATCAACAGGGAGGATCCTGGTACCAAA

>Rhsi-DAB3*07:01

CTGATGCTGTCGGCTTTTACTGGAACA-----------------------------------------------------------------------------------------------------------------------------------------------------------------------------------------------------------------------GCTGATGGATATTATCAATACGACATAGCTGAATGCTTCTACAGCACCAGTGATTACAGCGATATGGTGTATCTACGTTCATTTTCATTCAATAAAGTTGTGGATGTACAGTTCAACAGCACTTTGGGGAAGTGTGTGGGCTACACTGAACTTGGAGTAAGTAGTGCACGGAATTGGAACAGCGATCCCAACCGTCTGCAGCAAGAGAGAGCTGAGTTGGAGAGATACTGCAAACATAATGCTGAAATCAGACAGGCATCTATCGCT---GATAAAACA------------------------------------------------------------------------------------------------------------------------------------------------------------------------------------------------------------------------------------------------------------------------------------------------------------------------------------------------------------------------------------------------GTGGCACCAAAGGTCAAGCTCAGTTCAGTGACGCAGGCCGGCGGCAGACATCCTGCTGTACTGATGTGCAGCGCTTACCGCTTCTACCCGCACTGGATCAAAGTGTCCTGGATGAGAGACGGTACAGTTGTGAAGACTGATGTGACCTCAACTGAGGAGATGCCTAACGGAGACTGGTACTACCAGATTCACTCGCACCTGGAGTACACTCCCAAATCTGGAGAGAAGATCTCCTGTGTGGTGGATCACGCCGGCTTAACTAAATCCATCATCGTAGACTGG-----------------------------------------------------------------------------------------------------------------------------------------------------------------------GATCCCGCTATGCCTGAGTCTGACAGGAATAAAATCGCCATCGGGGCGTCTGGTCTGGTGCTGGGGATCATCATAGCAGCTGCTGGACTCATTTACTACAAGAAGAAGTCAACAGGGAGGATCCTGGTACCAAATC
